# Dissimilarity in the Chemical Behavior of Osmaoxazolium Salts and Osmaoxazoles: Two Different Aromatic Metalladiheterocycles

**DOI:** 10.1021/acs.organomet.1c00621

**Published:** 2021-12-14

**Authors:** María L. Buil, Miguel A. Esteruelas, Enrique Oñate, Nieves R. Picazo

**Affiliations:** Departamento de Química Inorgánica, Instituto de Síntesis Química y Catálisis Homogénea (ISQCH), Centro de Innovación en Química Avanzada (ORFEO-CINQA), Universidad de Zaragoza-CSIC, 50009 Zaragoza, Spain

## Abstract

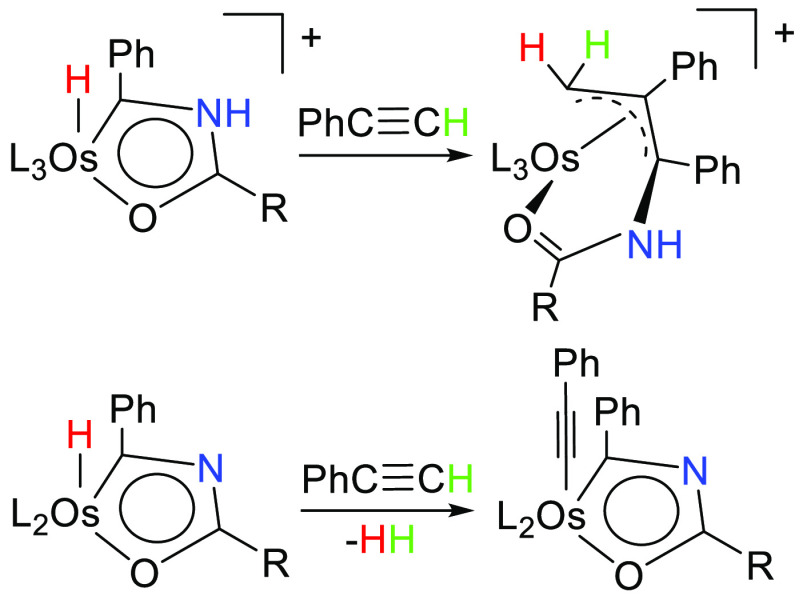

The preparation of aromatic hydride-osmaoxazolium and hydride-oxazole compounds is reported and their reactivity toward phenylacetylene investigated. Complex [OsH(OH)(≡CPh)(IPr)(P^i^Pr_3_)]OTf (**1**; IPr = 1,3-bis(2,6-diisopropylphenyl)imidazolylidene, OTf = CF_3_SO_3_) reacts with acetonitrile and benzonitrile to give [OsH{κ^2^-*C,O*-[C(Ph)NHC(R)O]}(NCR)(IPr)(P^i^Pr_3_)]OTf (R = Me (**2**), Ph (**3**)) via amidate intermediates, which are generated by addition of the hydroxide ligand to the nitrile. In agreement with this, the addition of 2-phenylacetamide to acetonitrile solutions of **1** gives [OsH{κ^2^-*C,O*-[C(Ph)NHC(CH_2_Ph)O]}(NCCH_3_)(IPr)(P^i^Pr_3_)]OTf (**4**). The deprotonation of the osmaoxazolium ring of **2** and **4** leads to the oxazole derivatives OsH{κ^2^-*C,O*-[C(Ph)NC(R)O]}(IPr)(P^i^Pr_3_) (R = Me (**5**), CH_2_Ph (**6**)). Complexes **2** and **4** add their Os–H and Os–C bonds to the C–C triple bond of phenylacetylene to afford [Os{η^3^-*C*_3_*,*κ^1^-*O*-[CH_2_C(Ph)C(Ph)NHC(R)O]}(NCCH_3_)_2_(IPr)]OTf (R = Me (**7**), CH_2_Ph (**8**)), bearing a tridentate amide-N-functionalized allyl ligand, while complexes **5** and **6** undergo a vicarious nucleophilic substitution of the hydride at the metal center with the alkyne, via the compressed dihydride adduct intermediates OsH_2_(C≡CPh){κ^2^-*C,O*-[C(Ph)NC(R)O]}(IPr)(P^i^Pr_3_) (R = Me (**9**), CH_2_Ph (**10**)), which reductively eliminate H_2_ to yield the acetylide-osmaoxazoles Os(C≡CPh){κ^2^-*C,O*-[C(Ph)NC(R)O]}(IPr)(P^i^Pr_3_) (R = Me (**11**), CH_2_Ph (**12**)).

## Introduction

**Chart 1:**
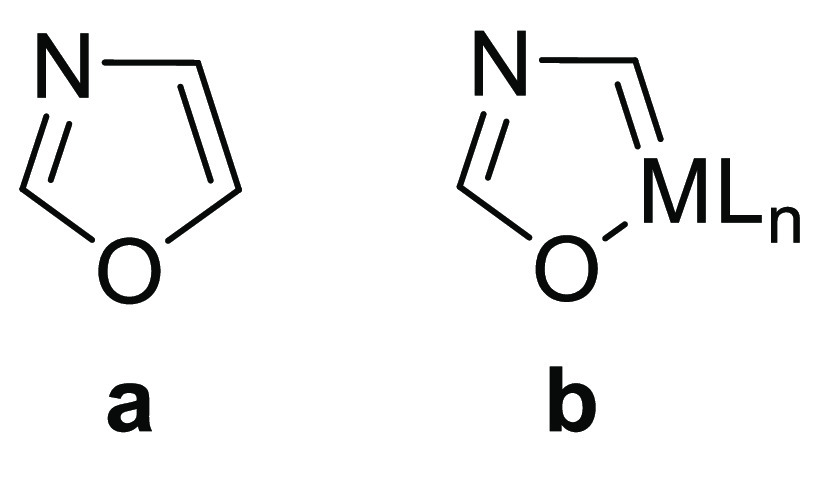
Oxazole and Metallaoxazole Rings

Oxazole is a five-membered aromatic heteromonocycle with oxygen and nitrogen at the 1- and 3-positions (**a** in Chart 1),^1^ which is present in a wide range of natural products^2^ and is gaining attention in recent times because of the relevance of the oxazole core in medical chemistry, since it is a fundamental part of several peptides displaying potential antibiotic and antitumor activity.^3^ As consequence of the successful therapeutic response to the treatment of a wide range of diseases, the synthesis of oxazole compounds has become a relevant objective of current chemistry and especially of pharmacology.^4^

**Chart 2:**
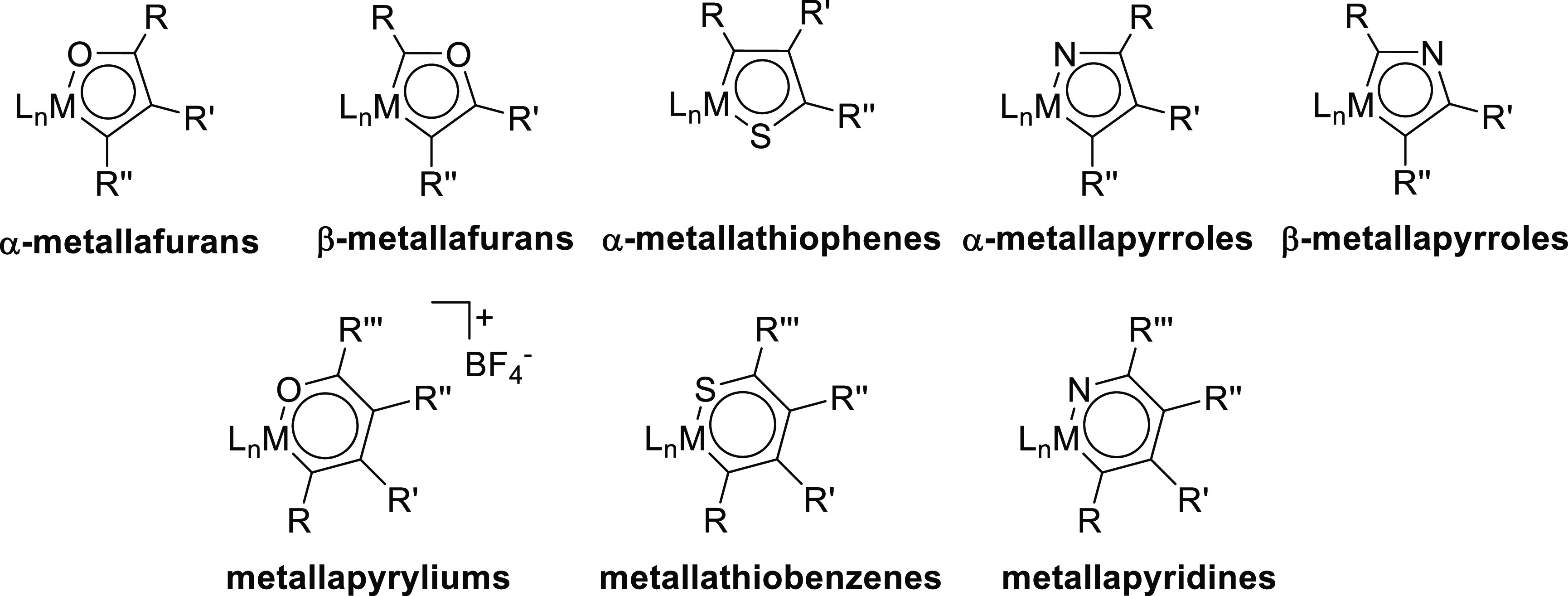
Known Organometallic Monocyclic Metallaheteroaromatic Rings

There is a class of aromatic organometallic compounds resulting from the formal replacement of a CH unit at an aromatic organic cycle by an isolobal metal fragment, formed by a transition metal and its associated ligands.^5^ Such a formal process achieved on an oxazole should afford a metallaoxazole (**b** in Chart 1). This class of metallaaromatic compounds should add organometallic reactivity to the reactions of the starting aromatic organic molecules. Since the prediction of the metallabenzenes by Thorn and Hoffmann in 1979^6^ and the preparation of the first osmabenzene by Roper and co-workers in 1982,^7^ the chemistry of these types of compounds has experienced a tremendous development, mainly from a conceptual point of view.^8^ Most of the effort has been centered on the metal counterparts of hydrocarbons: i.e., metallabenzenes,^9^ metallabenzynes,^10^ metallanaphthalenes,^11^ metallaanthracenes,^12^ metalloles,^13^ and some condensed species bearing the metal bonded to four carbons such as carbolongs^14^ and spiro metalloles.^15^ In contrast, organometallic metallaheteroaromatic compounds have received little attention.^16^ Although the number of known heteroaromatic, organically pure molecules is extremely large,^17^ only the existence of α-^18^ and β-metallafurans,^19^ α-metallathiophenes,^20^ α-^21^ and β-metallapyrroles,^22^ metallapyryliums,^23^ metallathiobenzenes,^24^ and metallapyridines^25^ has been demonstrated (Chart 2). In addition, several polycycle-type derivatives containing main-group heteroatoms have also been reported.^26^ In this context, it should be pointed out that monocyclic organometallic metallaheteroaromatic compounds bearing two main-group heteroatoms in the ring are unknown: i.e., a metallaoxazole is a class of five-membered monocyclic aromatic metalladiheteroring that has not been reported as far. A reason that would explain the lack of metalladiheteromonocycles could be the need to develop synthetic procedures of a more sophisticated nature, to introduce two main-group heteroatoms into the aromatic ring rather than to introduce only one. In this respect, the development of organometallic synthetic procedures involving the assembly of several chemical moieties on the metal coordination sphere^27^ (multicomponent organometallic synthesis) is challenging and should be addressed with greater effort.

Transition-metal hydroxide complexes are a group of weak hydroxo acids with underdeveloped organometallic chemistry.^28^ However, in spite of the small number of transformations carried out with these compounds, some of them have proved to display catalytic ability to promote relevant organic transformations^29^ and to perform reactions of interest in connection with materials science.^30^ Hydroxide compounds of platinum-group metals have especially been scarcely studied; the number of known osmium species is particularly small.^31^ Among the reported complexes, hydride-osmium-hydroxy derivatives are the most surprising and fascinating,^32^ since the reductive elimination of water is a reaction generally favored from a thermodynamic point of view.^33^ In 2015, we isolated the five-coordinate hydride-osmium-hydroxy complex [OsH(OH)(≡CPh)(IPr)(P^i^Pr_3_)]OTf (IPr = 1,3-bis(2,6-diisopropylphenyl)imidazolylidene, OTf = CF_3_SO_3_), as a result of a chloride by hydroxide replacement.^34^ This asymmetrical unsaturated species, supported on an unusual NHC-Os-P^i^Pr_3_ skeleton,^35^ possesses three potential reactive points in addition to the metal center: the hydride ligand, the hydroxide group, and the alkylidyne unit. As expected for a hydroxo acid, the hydroxide ligand expresses its duality in the respective nucleophilicity and electrophilicity of the oxygen and hydrogen atoms. Thus, it reacts with aldehydes to give carboxylate derivatives and molecular hydrogen.^36^ For its part, the osmium–alkylidyne bond undergoes hydroboration and hydrogenation reactions^34,37^ as well as insertion into the Os–H bond.^36^ The presence of three positions with organometallic reactivity, one of them carrying an oxygen atom, makes the complex an excellent candidate to attempt multicomponent reactions directed toward the synthesis of metallaoxazoles.

This paper reports organometallic multicomponent reactions on the cation [OsH(OH)(≡CPh)(IPr)(P^i^Pr_3_)]^+^. These reactions, which involve the coupling of a nitrile molecule, a hydroxide group, and an alkylidyne ligand at the metal coordination sphere, afford osmaoxazolium salts and subsequently osmaoxazoles. In addition, there is a surprising difference in reactivity, toward a terminal alkyne such as phenylacetylene, between the oxazolium and oxazole derivatives.

## Results and Discussion

### Osmaoxazolium Salts

**Scheme 1:**
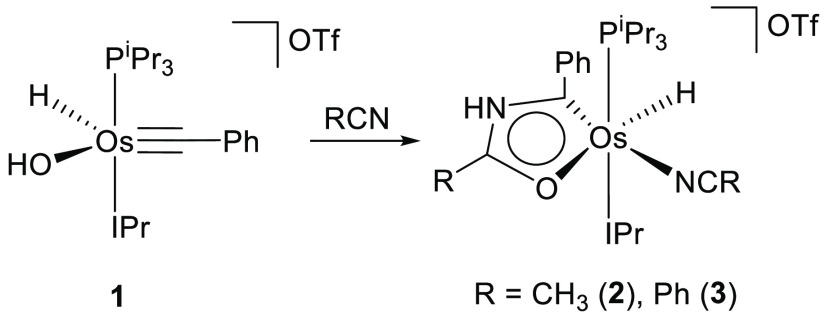
Preparation of **2** and **3**

The cation [OsH(OH)(≡CPh)(IPr)(P^i^Pr_3_)]^+^ (**1**) is unstable in nitrile solutions. Thus, stirring its OTf salt in acetonitrile and benzonitrile leads to the respective salts [OsH{κ^2^-*C,O*-[C(Ph)NHC(R)O]}(NCR)(IPr)(P^i^Pr_3_)]OTf (R = Me (**2**), Ph (**3**)). The new cations bear a five-membered osmaoxazolium metallacycle resulting from the coupling of a solvent molecule, the hydroxide group, and the alkylidyne ligand. The metal center prevents its electronic deficiency by means of the coordination of a second solvent molecule (Scheme 1).

**Figure 1:**
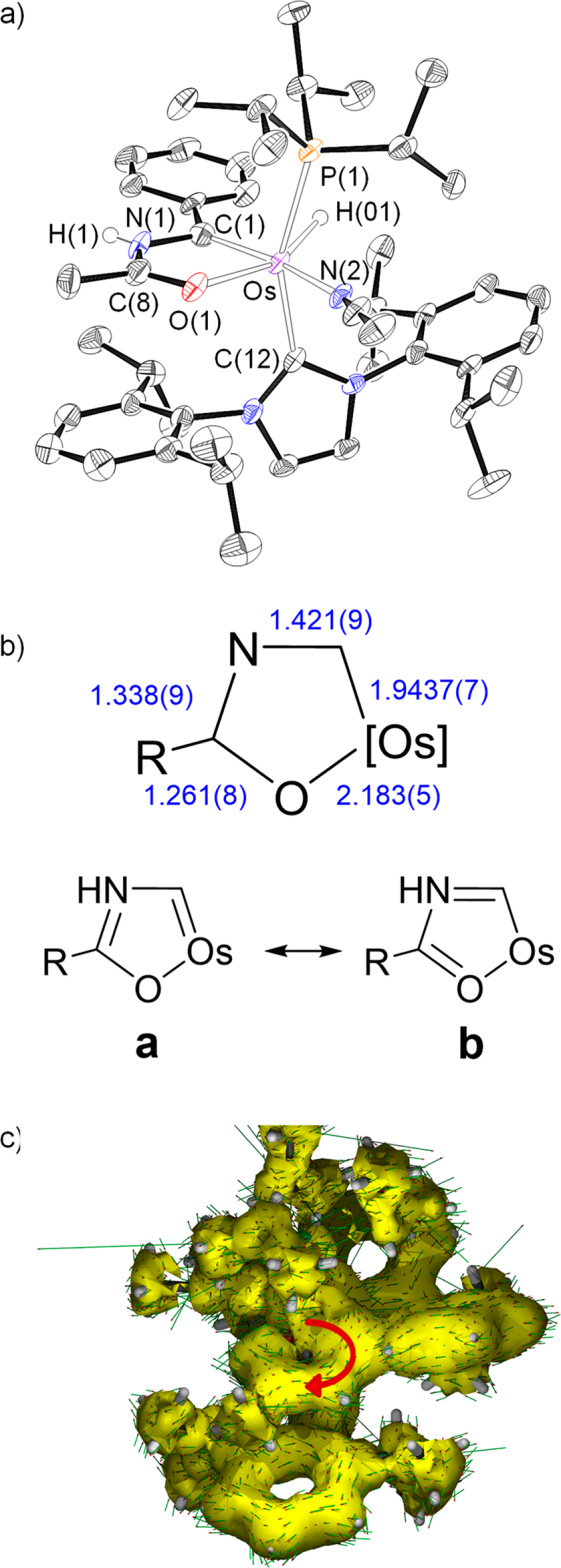
(a) X-ray structure of the cation of **2** (ellipsoids shown at 50% probability). All hydrogen atoms (except OsH and NH) are omitted for clarity. Selected bond distances (Å) and angles (deg): Os–P(1) = 2.3770(17), Os–C(1) = 1.937(7), Os–C(12) = 2.124(6), Os–O(1) = 2.183(5), Os–H(01) = 1.573(10), P(1)–Os–C(12) = 156.15(18), C(1)–Os–N(2) = 159.7(2), O(1)–Os–H(01) = 156(2), C(1)–Os–C(12) = 102.2(3), C(1)–Os–P(1) = 95.64(18). (b) Bond lengths and canonical forms describing the metallacycle bonding situation. (c) AICD plot with an isosurface value of 0.03. The red arrow indicates the direction of induced current.

Complexes **2** and **3** were isolated as purple and green solids in 74% and 62% yields, respectively. The formation of the metalladiheteromonocycle was confirmed by means of the X-ray structure of the acetonitrile derivative **2** (Figure 1a). An ideal coordination polyhedron around the osmium center can be described as a distorted octahedron with the bulky ligands phosphine and NHC situated mutually *trans* (P(1)–Os–C(12) = 156.15(18)°). The metallacycle is disposed perpendicular to an ideal P(1)–Os–C(1) direction with the C(1) atom located *trans* to the nitrile molecule (C(1)–Os–N(2) = 159.7(2)°), whereas the oxygen atom lies *trans* to the hydride ligand (O(1)–Os–H(01) = 156(2)°). The monocycle is planar. The maximum deviation from the best plane through the atoms Os, C(1), N(1), C(8), and O(1) is 0.014 Å and involves C(1) and N(1). Although the separation between the atoms is consistent with the existence of bonds intermediate between single and double, in agreement with an aromatic system, the bond length values reveal that from the two resonance forms contributing to the structure (Figure 1b), form a is more relevant than form b. Thus, for instance, the N(1)–C(8) bond length is about 0.08 Å shorter than the N(1)–C(1) distance (1.338(9) versus 1.421(9) Å). The aromaticity of the metallacycle is also supported by the negative values of the nuclear independent chemical shift (NICS) computed at the center of the ring and out of plane at 1 Å above and below the ring center; −5.1, −5.2, and −7.5 ppm, respectively. Furthermore, the anisotropy of the induced current density (ACID) method clearly shows the occurrence of a diatropic (clockwise vectors) ring current within the five-membered metalladiheteromonocycle (Figure 1c).

The NMR spectra of **2** and **3**, in acetonitrile-*d*_3_, at room temperature are consistent with the structure shown in Figure 1a and the aromatic character of the metallacycle. According to the presence of the hydride ligand in the complexes, the ^1^H spectra contain a doublet (^2^*J*_H–P_ ≈ 24.7 Hz) at −18.07 ppm for **2** and −16.87 ppm for **3**, whereas the NH resonance is observed as a singlet at 10.58 ppm for **2** and at 11.00 ppm for **3**. In the ^13^C{^1^H} spectra the OsC carbon atom gives rise to a doublet (^2^*J*_C–P_ ≈ 5.1 Hz) at 214.4 ppm for **2** and 213.2 ppm for **3**, whereas the signal corresponding to the NCO carbon atom appears as a singlet at 179.3 ppm for **2** and 175.7 ppm for **3.** A singlet at about 22 ppm in the ^31^P{^1^H} spectra is also characteristic of these compounds.

**Scheme 2:**
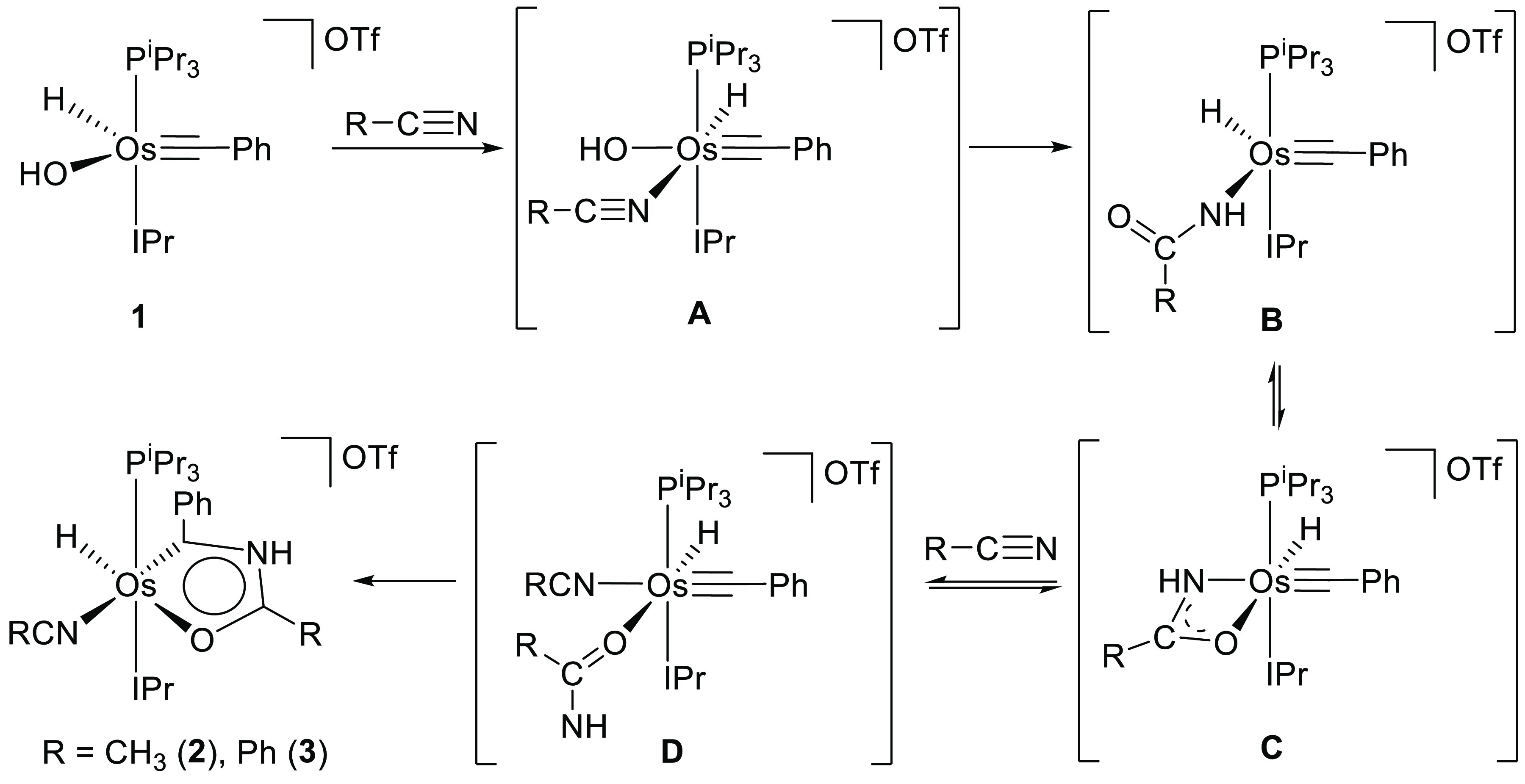
Proposal for the Formation of **2** and **3**

The formation of **2** and **3** can be rationalized according to Scheme 2. It has been recently demonstrated that hydroxide species are key players in the osmium-promoted catalytic hydration of nitriles. The coordination of the nitrile to the metal center enhances the electrophilicity of its C(sp) atom, which makes it more susceptible to undergo an intra- or intermolecular nucleophilic attack of the hydroxide group. The attack leads to metal-κ^1^-*N*-amidate derivatives, which are the true catalysts of the hydration.^38^ According to this, it seems reasonable to think that the first step in the formation of **2** and **3** is the coordination of a nitrile molecule to the metal center of the unsaturated cation **1** to afford the six-coordinate intermediate **A**. Thus, the nucleophilic attack of the hydroxide group to the coordinated nitrile could give the κ^1^-*N*-amidate intermediate **B** in equilibrium with the κ^2^*-N,O* and κ^1^-*O* counterpart**s C** and **D**. Subsequently, the electrophilic alkylidyne ligand would trap the free NH arm of **D** to yield **2** and **3**.

**Scheme 3:**
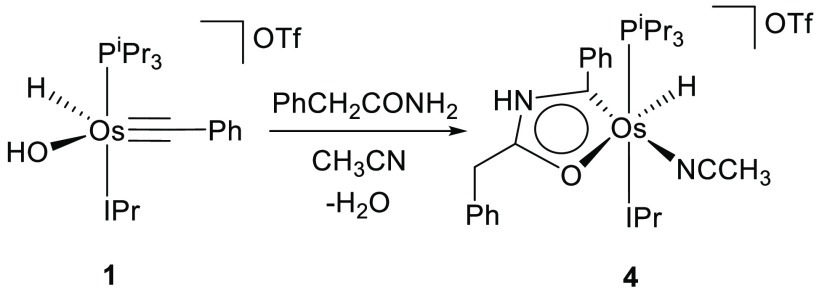
Formation of **4** by N–H Bond Activation of 2-Phenylacetamide

We reasoned that the formation of the amidate intermediate should also occur by the osmium-promoted N–H bond activation of an amide, where the hydroxide group would act as an internal base.^39^ In order to prove our hypothesis and to reinforce the proposal summarized in Scheme 2, we treated the OTf salt of **1** with 1.0 equiv of 2-phenylacetamide, in acetonitrile, at room temperature. As expected, the quantitative formation of the osmaoxazolium derivative [OsH{κ^2^-*C,O*-[C(Ph)NHC(CH_2_Ph)O]}(NCCH_3_)(IPr)(P^i^Pr_3_)]OTf (**4**) took place after 48 h (Scheme 3). Complex **4** was isolated as a purple solid in 64% yield. In agreement with **2** and **3**, its ^1^H NMR spectrum, in acetonitrile-*d*_3_, at room temperature displays a doublet (^2^*J*_H–P_ = 25.9 Hz) at −17.95 ppm due to the hydride ligand and a singlet at 10.76 ppm corresponding to the osmaoxazolium NH hydrogen atom. In the ^13^C{^1^H} NMR spectrum, the resonance corresponding to the OsC carbon atom of the metallacycle appears as a doublet (^2^*J*_C–P_ = 4.6 Hz) at 213.1 ppm, whereas the signal due to the NCO carbon atom is observed as a singlet at 180.1 ppm. The ^31^P{^1^H} NMR spectrum contains a singlet at 24.7 ppm.

### Osmaoxazole Derivatives

**Scheme 4:**
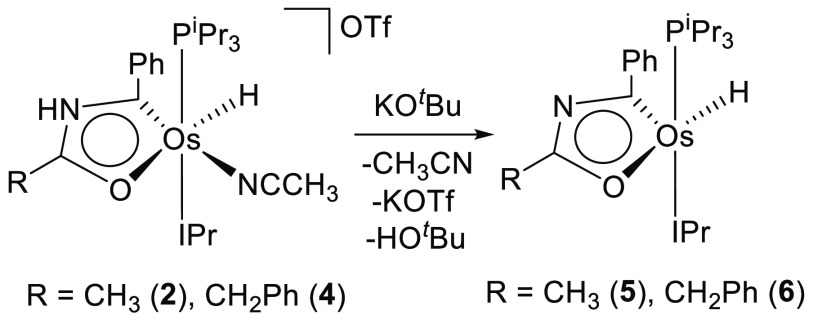
Transformation of **2** and **4** into **5** and **6**

In principle, these osmaoxazolium cations have two centers susceptible to deprotonation, the NH group and the MH position. However, the NH group displays stronger acidity in comparison to the MH position. Thus, the treatment of the tetrahydrofuran solutions of **2** and **4** with 1.0 equiv of potassium *tert*-butoxide, at room temperature, selectively produces the instantaneous abstraction of the NH hydrogen atom. The deprotonation appears to cause an adjustment of the electron density of the five-membered ring, which gives rise to the dissociation of the acetonitrile molecule from the metal center. The resulting five-coordinate osmaoxazole molecules OsH{κ^2^-*C,O*-[C(Ph)NC(R)O]}(IPr)(P^i^Pr_3_) (R = Me (**5**), CH_2_Ph (**6**)) were isolated as brown solids in about 80% yield (Scheme 4).

**Figure 2:**
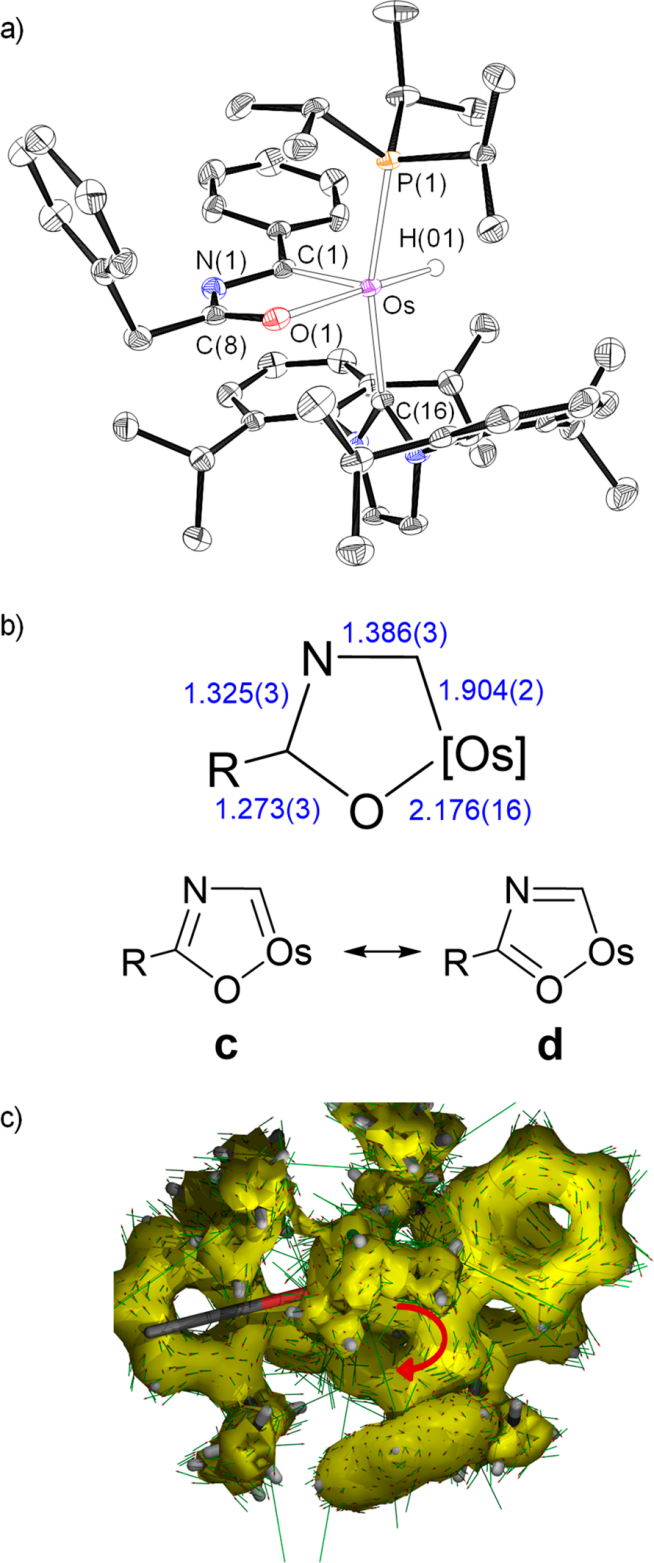
(a) X-ray structure of complex **6** (ellipsoids shown at 50% probability). All hydrogen atoms (except the hydride) are omitted for clarity. Selected bond distances (Å) and angles (deg): Os–C(1) = 1.904(2), Os–C(16) = 2.077(2), Os–O(1) = 2.1766(16), Os–P(1) = 2.3387(6), Os–H(01) = 1.576 (10), O(1)–Os–H(01) = 178.4(9), C(16)–Os–P(1) = 164.43(6), O(1)–Os–P(1) = 100.54(5), C(16)–Os–O(1) = 86.33(7). (b) Bond lengths and canonical forms describing the metallacycle bonding situation. (c) AICD plots of complex **6** with an isosurface value of 0.03. The red arrow indicates the direction of induced current.

The formation of **5** and **6** was confirmed by means of the X-ray structure of **6** (Figure 2a). The coordination polyhedron around the osmium atom can be idealized as a square pyramid with the C(1) atom of the five-membered ring at the apical position, whereas the base is formed by the oxygen atom O(1) disposed *trans* to the hydride ligand (O(1)–Os–H(01) = 178.4(9)°) and the phosphine and NHC ligands that are also situated mutually *trans* (C(16)–Os–P(1) = 164.43(6)°). As in **2**, the metallacycle is planar. In this case, the maximum deviation from the best plane through the atoms of the ring is 0.0518(12) Å and involves C(1). The deprotonation produces a slight shortening of the bond lengths within the ring. The distances between atoms suggest that from the two resonance forms participating in the monocycle structure, c and d (Figure 2b), the contribution of the former (analogous to a in Figure 1b) is greater than the contribution of the latter. The NICS values computed at the center of the ring and out of plane at 1 Å above and below the ring center are also negative, −2.8, −3.4, and −6.5 ppm, respectively, although they are slightly higher than those of **2**. As in the latter, the ACID method displays the expected diatropic ring current, in agreement with the aromatic nature of the metalladiheterocycle (Figure 2c).

The NMR spectra of **5** and **6** in toluene-*d*_8_ are consistent with the structure shown in Figure 2a. In the ^1^H spectra, at 253 K, the most noticeable features are the absence of any NH resonance and the presence of a doublet (^2^*J*_H–P_ ≈ 20 Hz) at −12.73 ppm for **5** and −15.35 ppm for **6**, assigned to the hydride ligand. The ^13^C{^1^H} spectra show the resonance corresponding to the OsC carbon atom of the metallacycle at 236.9 ppm for **5** and at 230.8 ppm for **6**, shifted by about 20 ppm toward lower field with regard to the osmaoxazolium counterpart, in a manner consistent with the shortening of the Os–C bond of the five-membered ring as a consequence of its deprotonation, whereas the signal due to the NCO carbon atom appears at about 189 ppm. The ^31^P{^1^H} spectra contain a singlet at 40.8 ppm for **5** and at 43.8 ppm for **6**.

### Reactions with Phenylacetylene

The transformation observed in the metal coordination sphere, as a consequence of the deprotonation of the metalladiheterocycle, drew our attention because it pointed out that the contribution of the free pair of the nitrogen atom to the π electronic cloud of the monocycle is relevant enough to significantly modify the chemical reactivity of the system. To confirm this perception, we decided to study the behavior of both osmaoxazolium cations and osmaoxazole molecules toward a terminal alkyne such as phenylacetylene.

**Scheme 5:**
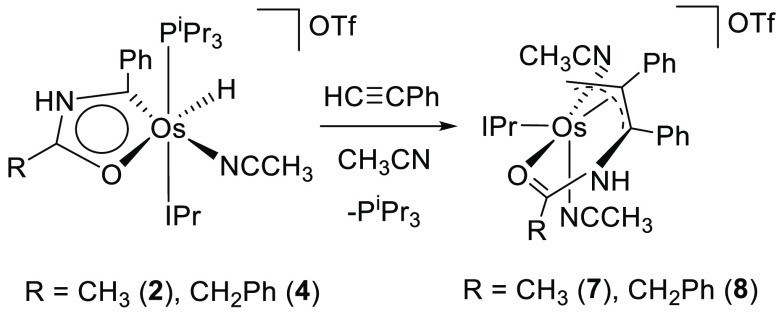
Reactions of **2** and **4** with Phenylacetylene

Osmaoxazolium cations undergo a novel 3 + 2 condensation with the alkyne, involving the Os–H and Os–C bonds of the aromatic system and the C–C triple bond of phenylacetylene. The addition, which should be favored by the resonance form a, is regioselective, leads to an allyl ligand N-functionalized with an amide and causes the displacement of the bulky phosphine ligand. The position of the latter is subsequently occupied by an acetonitrile molecule, which is the reaction solvent (Scheme 5). The transformation takes place at room temperature, using stoichiometric amounts of reagents. The reaction rate shows a marked dependence of the R substituent of the five-membered ring, methyl or benzyl. In this context, it should be mentioned that, while the quantitative formation of the salt [Os{η^3^-*C*_3_*,*κ^1^*-O-*[CH_2_C(Ph)C(Ph)NHC(Me)O]}(NCCH_3_)_2_(IPr)]OTf (**7**) requires 5 days, the salt [Os{η^3^-*C*_3_,κ^1^*-O-*[CH_2_C(Ph)C(Ph)NHC(CH_2_Ph)O]}(NCCH_3_)_2_(IPr)]OTf (**8**) is isolated in 96% yield after 24 h. Both salts are yellow solids.

**Figure 3:**
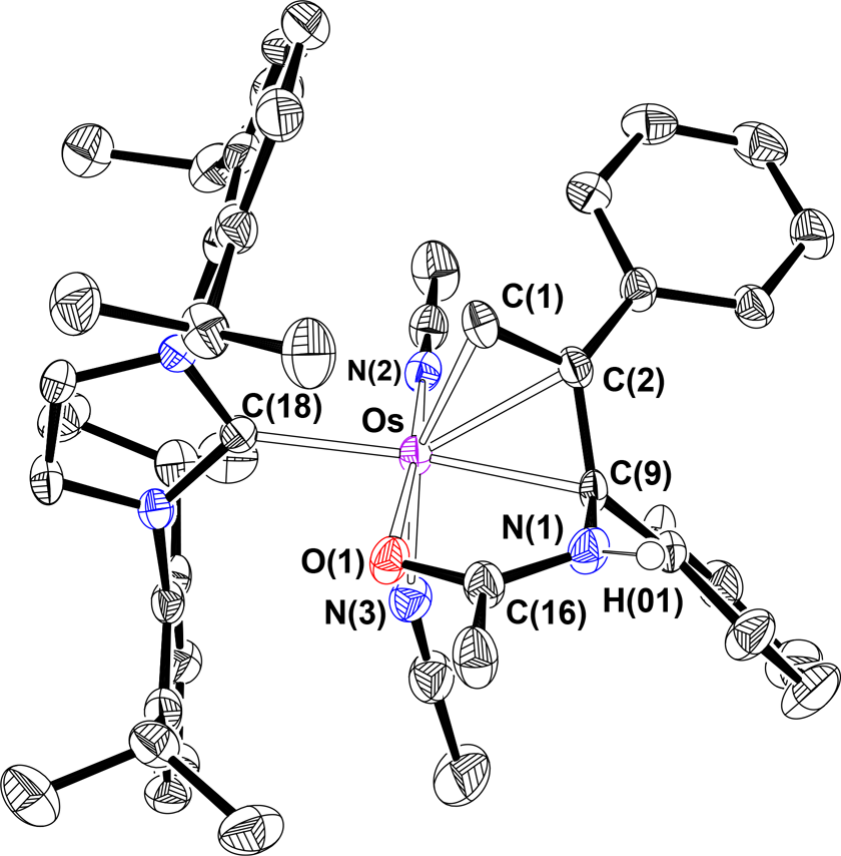
X-ray structure of complex **7** (ellipsoids shown at 50% probability). All hydrogen atoms are omitted for clarity (except NH). Selected bond distances (Å) and angles (deg): Os–C(1) = 2.154(6), Os–C(2) = 2.182(6), Os–C(9) = 2.172(5), Os–O(1) = 2.089(4), Os–C(18) = 2.087(6), Os–N(2) = 1.974(5), Os–N(3) = 2.087(6), C(1)–C(2) = 1.446(9), C(2)–C(9) = 1.447(8), N(2)–Os–O(1) = 173.5(2), N(3)–Os–C(1) = 159.4(2), C(18)–Os–C(9) = 159.4(2), C(1)–C(2)–C(9) = 115.3(5).

The regioselective formation of the functionalized allyl ligand is supported by the X-ray structure of the salt **7**, which was isolated in 88% yield. Figure 3 gives a view of the cation. The geometry around the metal center can be idealized as a distorted octahedron, where the functionalized allyl acts as a tridentate group occupying a face. At the other face, one of the acetonitrile molecules occupies a *trans* position with regard to the coordinated oxygen atom of the amide function (N(2)–Os–O(1) = 173.5(2)°), whereas the other acetonitrile molecule lies *trans* to the terminal C(1) atom of the allyl (N(3)–Os–C(1) = 159.4(2)°). The IPr ligand is situated *trans* to the N-functionalized C(9) atom (C(18)–Os–C(9) = 159.4(2)°). The allyl moiety coordinates in an asymmetrical fashion, with Os–C(1), Os–C(2), and Os–C(9) distances of 2.154(6), 2.182(6), and 2.172(5) Å, respectively. The allylic angle C(1)–C(2)–C(9) of 115.3(5)° as well as the C(1)–C(2) and C(2)–C(9) bond lengths of 1.446(9) and 1.447(8) Å are in accordance with the values reported for other osmium π-allyl compounds.^40^ The NMR spectra of **7** and **8**, in acetonitrile-*d*_3_, at room temperature are consistent with the presence of the amide-allyl ligand in the cations. Noticeable features of this group in the ^1^H spectra are a singlet at 8.50 ppm for **7** and at 7.64 ppm for **8**, corresponding to the NH hydrogen atom, and two doublets (^2^*J*_H–H_ = 5.8 Hz) at about 1.8 and 1.0 ppm due to the allylic CH_2_ moiety. In the ^13^C{^1^H} spectra the resonances assigned to the amide-allyl ligand are observed at around 182 (NCO), 93 (NCPh), 76 (OsCPh), and 30 (CH_2_) ppm.

The formation of the amide-allyl ligand of **7** and **8** merits some additional comment, since it is a multicomponent coupling on the coordination sphere of a transition metal rarely observed in organometallic chemistry. It involves the coupling of two organic molecules (the alkyne and the nitrile) and three ligands of the starting cation (the hydride, the hydroxide, and the alkylidyne). As viewed, the coupling occurs in two separate stages in a sequential manner. During the first stage the external nitrile and the hydroxide form an amidate on the osmium coordination sphere, which is subsequently trapped by the alkylidyne to afford an aromatic osmaoxazolium ring. In the second stage, the external alkyne is added to the Os–H and Os–C bonds of the generated organometallic system. In connection with the addition of the alkyne to the hydride-osmaoxazolium moiety, we note that Paneque, Poveda, and co-workers have previously studied the addition of olefins and alkynes to hydride-iridafuran compounds. In contrast to the coupling shown in Scheme 5, they observed a 1,2-hydride shift from the metal to the metalated carbon atom followed by the 1,3-addition of the external unsaturated bond to the resulting metallacycle, to form a bicyclic system.^41^ Iridabicycle compounds have been also generated by 1,2- and 1,3-additions of acetone^42^ and alkynes to iridapyrilium^23^ and iridathiobenzene^43^ complexes. In addition, Xia and co-workers have reported the preparation of 9- and 10-membered osmacycles by metathesis between alkynes and osmafurans^44^ and by reaction of osmapyridinium and propargyl alcohols,^45^ respectively.

**Scheme 6:**
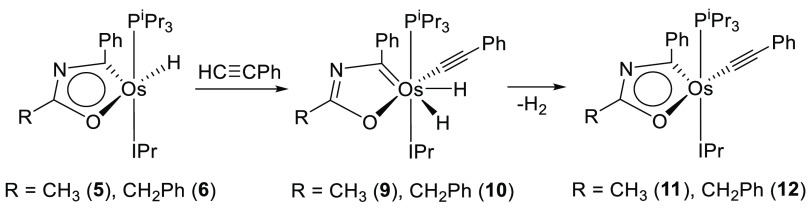
Reactions of **5** and **6** with Phenylacetylene

There is certainly a very marked difference in behavior toward phenylacetylene between the salts **2** and **4** and the respective counterpart molecules **5** and **6**. In contrast to **2** and **4**, the metal center of **5** and **6** undergoes an oxidative addition of the C(sp)–H bond of the alkyne, to form the compressed-dihydride^46^ derivatives OsH_2_(C≡CPh){κ^2^-*C,O*-[C(Ph)NC(R)O]}(IPr)(P^i^Pr_3_) (R = Me (**9**), CH_2_Ph (**10**)). The addition occurs at room temperature and is fast and quantitative, although the new yellow osmium(IV) species were isolated in moderate yields (∼50%) as a consequence of their moderate solubility in the usual organic solvents (Scheme 6). The presence of the compressed dihydrides in the complexes was inferred from the ^1^H NMR spectra in toluene-*d*_8_. These spectra show a doublet (^2^*J*_H–P_ ≈ 11.7 Hz) at about −8.9 ppm, which exhibits a 300 MHz *T*_1_(min) value of 44 ± 4 ms, whereas the H–D coupling constant in the partially deuterated species is 5 Hz. These values allow calculating a separation between the hydrides of about 1.34 Å.^47^ The oxidation of the metal center and its coordinative saturation produces an important increase in the contribution of the resonance form c to the metalladiheteromonocycle structure. This is strongly supported by the ^13^C{^1^H} NMR spectra, which contain a doublet (^2^*J*_C–P_ ≈ 2.5 Hz) generated by the OsC carbon atom of the ring at about 274 ppm: i.e., shifted nearly 40 ppm toward lower field with regard to those of **5** and **6**. In addition, the spectra show the resonance due to the NCO carbon atom at about 193 ppm and the characteristic resonances corresponding to the alkynyl ligand, which are observed around 117 and 109 ppm as doublets with C–P coupling constants of 1 and 15 Hz, respectively. The ^31^P{^1^H} NMR spectra display a singlet at around 7 ppm.

**Figure 4:**
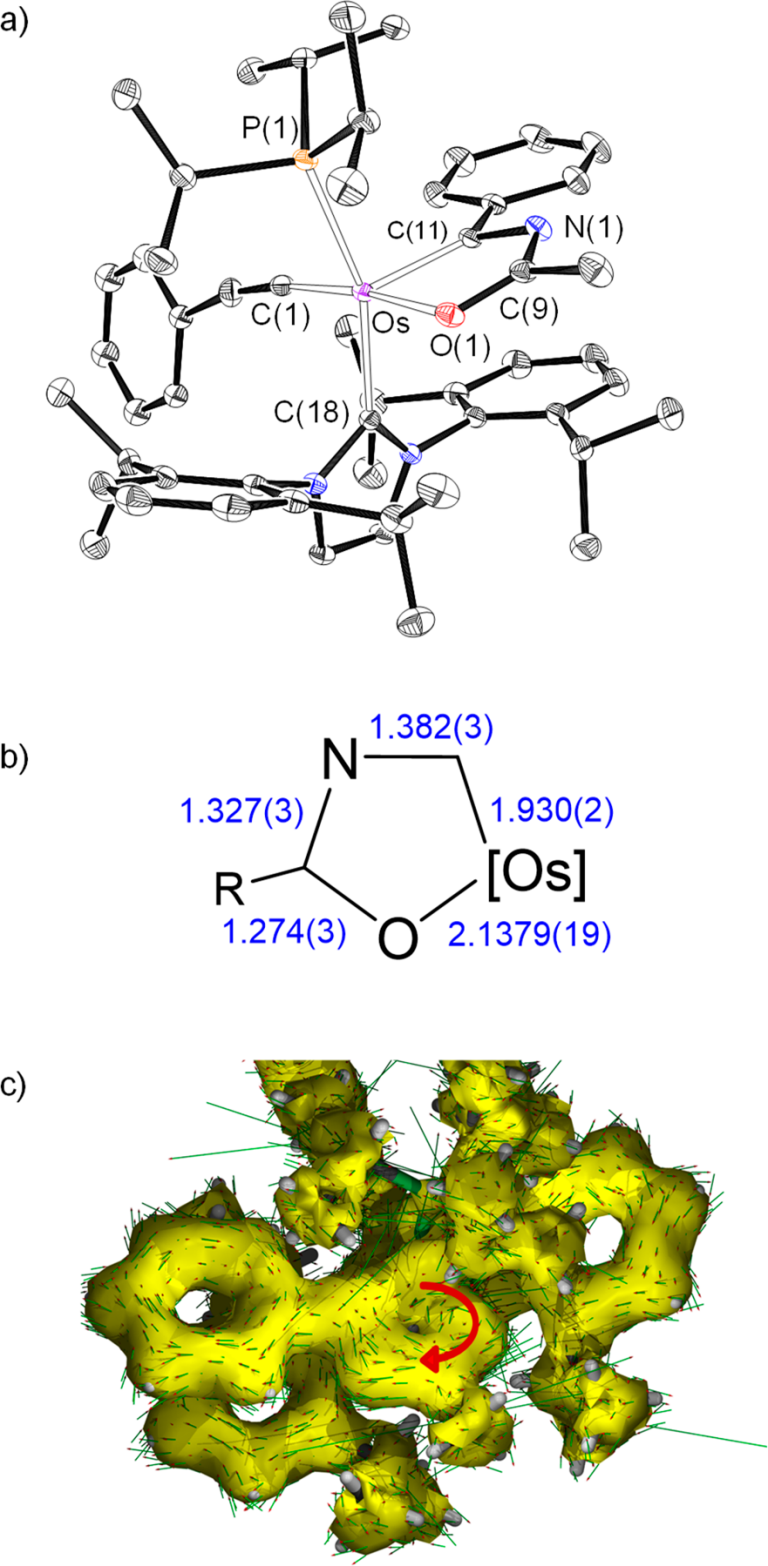
(a) X-ray structure of complex **11** (ellipsoids shown at 50% probability). All hydrogen atoms are omitted for clarity. Selected bond distances (Å) and angles (deg): Os–C(11) = 1.930(2), Os–C(1) = 1.988(3), Os–C(18) = 2.094(2), Os–O(1) = 2.1379(19), Os–P(1) = 2.3649(7), C(1)–Os–O(1) = 172.96(9), C(18)–Os–O(1) = 93.02(8), C(18)–Os–P(1) = 159.87(7), O(1)–Os–P(1) = 92.22(5). (b) Bond lengths in the metallacycle. (c) AICD plot of complex **11** with an isosurface value of 0.03. The red arrow indicates the direction of induced current.

Complexes **9** and **10** are intermediate species in the substitution process of hydride by acetylide in **5** and **6**. Thus, they lose molecular hydrogen, in toluene, at 70 °C to give the respective acetylide-osmaoxazole derivatives Os(C≡CPh){κ^2^-*C,O*-[C(Ph)NC(R)O]}(IPr)(P^i^Pr_3_) (R = Me (**11**), CH_2_Ph (**12**)). The substitution products were isolated as purple solids in about 65% yield. The replacement was confirmed by the X-ray structure of **11** (Figure 4a). The ring bond lengths compare well with those found in **6** (Figure 4b), whereas the coordination polyhedron around the osmium atom resembles that of the latter with the acetylide at the hydride position and angles at the pyramid base of 172.96(9)° (C(1)–Os–O(1)) and 159.87(7)° (C(18)–Os–P(1)). The release of the hydrogen molecule reestablishes the electronic situation in the ring, which becomes similar to that of **5** and **6**. Thus, the NICS values computed at the center of the ring and out of plane at 1 Å above and below the ring center, −3.7, −5.3, and −5.5 ppm, compare well with those of **6**. As for the latter, the ACID method shows a diatropic ring current in accordance with the aromatic character of the metallacycle (Figure 4c). The rebalancing of the electronic situation in the ring is also done, as is evident in the chemical shift of the resonances corresponding to the carbon atoms of metalladiheteromonocycle in the ^13^C{^1^H} NMR spectra, in toluene-*d*_8_, which are similar to those observed in the spectra of **5** and **6**. Thus, the resonance corresponding to the OsC carbon atom appears at about 243 ppm, whereas the signal due to the NCO carbon atom is observed near 191 ppm. The spectra furthermore contain the characteristic signals due to the C(sp) carbon atoms of the alkynyl ligand at about 126 and 118 ppm. The spectra of ^31^P{^1^H} show a singlet at around 27 ppm.

Nucleophiles bearing leaving groups at the nucleophile center displace hydrogen from the electron-deficient positions of aromatic compounds. The reaction is known in organic chemistry as “vicarious nucleophilic substitution of hydrogen” and represents a general, direct method to introduce C, O, and N substituents in electron-deficient aromatic rings. Initially, the NuX nucleophile adds to the aromatic compound to afford an intermediate σ^H^ adduct, which subsequently undergoes a base-promoted HX abstraction.^48^ The hydride by acetylide substitution shown in Scheme 6 is an original example of this class of reaction: as far as we know, the first case observed on an aromatic metallacycle. The addition is facilitated by the unsaturation of the metal center and the marked polarity of the C(sp)–H bond of the alkyne, whereas the easy diffusion of the generated gas acts as a driving force for the product formation. In contrast to the classical organic reaction, a base is not necessary to reach the final products, since the addition adducts (complexes **10** and **11**) are able to eliminate the byproduct of the substitution (H_2_) by reductive elimination. From a mechanistic point of view, it should be pointed out that the elimination of the byproduct is the rate-determining step of the substitution, as occurs in the organic reaction for low base concentrations.^49^

Once the difference in chemical behavior between the salts **2** and **4** and the molecules **5** and **6** was confirmed, we analyzed the frontier orbitals of the cation **2** and complex **6** (B3LYP-D3//SDD(f)/6-31G**) to gain information about the motive of such differences. The HOMO of **2** is mainly centered on the metal (76%), whereas the LUMO spreads over the metalladiheteromonocycle (38%), the phenyl substituent (29%), and the metal center (20%). In complex **6**, the situation is similar. The HOMO is almost exclusively centered on the osmium atom (90%), whereas the LUMO is delocalized through the metalladiheteromonocycle (39%), the phenyl substituent (33%), the osmium atom (15%), and the IPr ligand (10%). Because the distributions of the frontier orbitals in both types of species were very similar and the insignificant discrepancies in the orbital distribution did not justify the difference in chemical behavior observed, we subsequently analyzed the NBO charges on the hydride ligand, the metal center, and the OsC carbon atom of the metalladiheteromonocycle. In contrast to the frontier orbitals, in this case there are some notable variations. While the charge on the hydride ligand of the cation **2** is slightly positive (0.055), the hydride of **6** displays a strongly basic character with a negative charge of −0.129. In agreement with the HOMO concentration on the metal center, the charge of the latter is strongly negative in both species, fitting a more strongly basic metal center for a more acidic hydride ligand. Thus, the value of the negative charge on the osmium atom on **2** is higher than that on **6** (−0.605 versus −0.256). In contrast to the metal center, the OsC carbon atom bears a positive charge in both compounds: 0.182 for **2** and 0.255 for **6**. Although these results do not give a complete picture of the reason cations **2** and **4** react with phenylacetylene in a manner different from that of complexes **5** and **6**, they suggest that the difference in behavior is related to the charges on the atoms involved, in particular the charge on the hydride ligand, while the dissimilarity does not depend upon the frontier orbitals of the complexes. A slightly positive hydride ligand and a positive metalated carbon atom at the metallacyle favor the addition of the C–C triple bond of the alkyne to the Os–H and Os–C bonds of the hydride-osmaoxazolium unit. However, a strongly basic hydride promotes a hydride by acetylide nucleophilic substitution at the metal center.

## Concluding Remarks

This study reveals that it is possible to prepare hydride-osmaoxazolium salts by means of the reaction of a nitrile and a cationic hydride-hydroxy-osmium(II)-alkylidyne complex. The formation of the monocycle takes place via an amidate intermediate, which is generated by the addition of the hydroxide group of the starting cation to the nitrile. Once the amidate is generated, it cyclizes with the alkylidyne ligand to form the five-membered ring. Its deprotonation affords osmaoxazole molecules, which display reactivity toward phenylacetylene surprisingly different from the reactivity observed for the oxazolium salts. While the Os–H and Os–C bonds of the hydride-osmaoxazolium moiety of the cations of these salts are added to the C–C triple bond of the alkyne, to generate a tridentate amide-N-functionalized allyl ligand, the hydride-osmaoxazole molecules undergo a vicarious nucleophilic substitution of hydride at the metal center, with the alkyne, via a dihydride-osmium(IV)-acetylide adduct intermediate.

In conclusion, two monocyclic aromatic metalladiheterocycles have been generated on the metal center of an osmium complex, through an organometallic multicomponent coupling reaction involving an external nitrile molecule and two ligands of the metal coordination sphere. In addition, the chemical behaviors of the rings toward a terminal alkyne have been analyzed.

## Experimental Section

### General Information

The reactions were carried out under argon using dry solvents. Instrumental methods are given in the Supporting Information. Chemical shifts (ppm) in the NMR spectra (Figures S1–S36) are referenced to residual solvent peaks; coupling constants are given in Hz. Signals were assigned through two-dimensional experiments (^1^H–^1^H COSY, ^1^H–^13^C{^1^H} HMBC, and ^1^H–^13^C{^1^H} HSQC). The starting complex **1** was prepared by a method previously reported.^35d^

### Preparation of [OsH{κ^2^-*C,O*-[C(Ph)NHC(CH_3_)O]}(NCCH_3_)(IPr)(P^i^Pr_3_)]OTf (**2**)

A yellow solution of [OsH(OH)(≡CPh)(IPr)(P^i^Pr_3_)]OTf (**1**; 100 mg, 0.12 mmol) in acetonitrile (5 mL) was heated at 70 °C for 18 h. The resulting mixture was filtered over Celite and was concentrated almost to dryness. The addition of diethyl ether gave rise to a purple solid, which was washed with diethyl ether (3 × 3 mL) and was dried *in vacuo*. Yield: 83 mg (74%). Crystals of **2** suitable for an X-ray diffraction analysis were obtained by diffusion of a tetrahydrofuran–pentane solution of the precipitate at 4 °C in a drybox. Anal. Calcd for C_48_H_70_F_3_N_4_O_4_OsPS: C, 53.51; H, 6.55; N, 5.20; S, 2.98. Found: C, 53.19; H, 6.74; N, 5.49; S, 3.10. MS (electrospray, *m*/*z*): C_45_H_66_N_3_OOsP [M – H – CH_3_CN], 887.4552; found, 887.4577. IR (cm^–1^): ν(N–H) 3258 (w); ν(Os–H) 2112 (w). ^1^H NMR (400 MHz, CD_3_CN, 298 K): δ 10.58 (s, 1H, N*H*), 7.92 (d, 2H, ^3^*J*_H–H_ = 7.3, *o*-Ph), 7.48–7.27 (m, 11H, Ph + CPh + *H*-IPr), 1.99 and 1.98 (both s, 3H each, NCC*H*_3_, O = CC*H*_3_), 1.78 (m 3H, PC*H*CH_3_), 1.71 (d, 6H, ^3^*J*_H–H_ = 6.8, CHC*H*_3_), 1.14 (m, 4H, C*H*CH_3_), 1.13 (d, 6H, ^3^*J*_H–H_ = 6.8, CHC*H*_3_), 0.84 (dd, 9H, ^2^*J*_H–P_ = 13.1, ^3^*J*_H–H_ = 7.2, PCHC*H*_3_ + 3H, CHC*H*_3_), 0.40 (dd, 9H, ^2^*J*_H–P_ = 13.2, ^3^*J*_H–H_ = 7.2, PCHC*H*_3_ + 3H, CHC*H*_3_), −18.07 (d, 1H, ^2^*J*_H–P_ = 24.7, Os-*H*). ^1^H NMR (400 MHz, CD_3_CN, 263 K, unobserved signals in aliphatic region at room temperature): δ 1.39 (d, 3H, ^3^*J*_H–H_ = 6.3, CHC*H*_3_), 1.14 (d, 3H, ^3^*J*_H–H_ = 6.5, CHC*H*_3_). ^31^P{^1^H} NMR (121 MHz, CD_3_CN, 298 K): δ 22.3 (s). ^13^C{^1^H} NMR plus HMBC and HSQC (75 MHz, CD_3_CN, 298 K): δ 214.4 (d, ^2^*J*_C–P_ = 5.1, Os*C*), 179.3 (s, N*C*O), 168.8 (d, ^2^*J*_C–P_ = 58.8, N*C*N), 152.6 (s, C_*ipso*_-Ph) 145.8 (s, *C*_*ipso*_-CPh + *C*_*o*_-CPh), 129.8, 129.3, 128.7, 128.2, 125.8, and 123.6 (all s, Ph + CPh + *C*-IPr), 29.3 and 28.4 (both s, *C*HCH_3_), 26.1 (d, ^3^*J*_C–P_ = 25.7, P*C*HCH_3_), 25.1, 21.8, and 18.7 (all s, CH*C*H_3_), 18.1 and 17.9 (both s, PCH*C*H_3_).

### Preparation of [OsH{κ^2^-*C,O*-[C(Ph)NHC(Ph)O]}(NCPh)(IPr)(P^i^Pr_3_)]OTf (**3**)

A yellow solution of [OsH(OH)(≡CPh)(IPr)(P^i^Pr_3_)]OTf (**1**; 100 mg, 0.12 mmol) in benzonitrile (5 mL) was stirred at room temperature. After 2 days, the dark green solution was filtered over Celite and was concentrated almost to dryness. The addition of pentane gave rise to the precipitation of a green solid, which was washed with pentane (3 × 3 mL) and was dried *in vacuo*. Yield: 78 mg (62%). Anal. Calcd for C_58_H_74_F_3_N_4_O_4_OsPS: C, 57.98; H, 6.21; N, 4.66; S, 2.67. Found: C, 58.32; H, 6.52; N, 5.03; S, 3.05. MS (electrospray, *m*/*z*): C_50_H_68_N_3_OOsP [M – H – PhCN], 949.4709; found, 949.4699. IR (cm^–1^): ν(Os–H) 2194 (w). ^1^H NMR (300 MHz, CD_3_CN, 298 K): δ 11.00 (s, 1H, N*H*), 7.97 (d, 2H, ^3^*J*_H–H_ = 7.1, *o*-Ph), 7.87 (d, 2H, ^3^*J*_H–H_ = 7.2, *o*-Ph), 7.73–7.26 (m, 19H, Ph + CPh + *H*-IPr), 1.85 (m, 3H, PC*H*CH_3_), 1.65 (d, 3H, ^3^*J*_H–H_ = 6.7, CHC*H*_3_), 1.19–1.02 (br, 22H, C*H*CH_3_ + CHC*H*_3_), 0.87 (dd, 9H, ^2^*J*_H–P_ = 13.2, ^3^*J*_H–H_ = 7.4, PCHC*H*_3_ + 3H CHC*H*_3_), 0.35 (dd, 9H, ^2^*J*_H–P_ = 13.3, ^3^*J*_H–H_ = 7.2, PCHC*H*_3_), −16.87 (d, 1H, ^2^*J*_H–P_ = 24.8, Os-*H*). ^31^P{^1^H} NMR (121 MHz, CD_3_CN, 298 K): δ 21.6 (s). ^13^C{^1^H} NMR plus HMBC and HSQC (75 MHz, CD_3_CN, 298 K): δ 213.2 (d, ^2^*J*_C–P_ = 5.2, Os*C*), 175.7 (s, N*C*O), 167.0 (d, ^2^*J*_C–P_ = 57.8, N*C*N), 152.7 and 152.6 (both s, *C*_*ipso*_-Ph), 146.1, 145.7, and 145.5 (all s, *C*_*ipso*_-CPh + C_*o*_-CPh), 132.9 132.1, 131.9, 127.8, 125.9, 123.2, and 122.9 (all s, Ph + CPh + *C*-IPr), 29.1, 28.7, and 28.3 (all s, *C*HCH_3_), 26.1 (d, ^3^*J*_C–P_ = 25.8, P*C*HCH_3_), 25.1, 24.0, 23.0, and 19.4 (all s, CH*C*H_3_), 18.1 and 17.8 (both s, PCH*C*H_3_).

### Preparation of [OsH{κ^2^-*C,O*-[C(Ph)NHC(CH_2_Ph)O]}(NCCH_3_)(IPr)(P^i^Pr_3_)]OTf (**4**)

A yellow solution of [OsH(OH)(≡CPh)(IPr)(P^i^Pr_3_)]OTf (**1**; 100 mg, 0.12 mmol) in acetonitrile (5 mL) was treated with 2-phenylacetamide (15 mg, 0.11 mmol). The resulting mixture was stirred for 48 h at room temperature. After this time, the resulting mixture was filtered over Celite and was concentrated almost to dryness. Addition of diethyl ether caused the formation of a purple solid that was washed with diethyl ether (3 × 3 mL) and was dried *in vacuo*. Yield: 76 mg (64%). Anal. Calcd for C_54_H_74_F_3_N_4_O_4_OsPS: C, 56.23; H, 6.47; N, 4.86; S, 2.78. Found: C, 56.25; H, 6.86; N, 4.47; S, 3.00. MS (electrospray, *m*/*z*): C_51_H_71_N_3_OOsP [M – CH_3_CN], 964.4955; found, 964.4955. IR (cm^–1^): ν(Os–H) 2963 (w). ^1^H NMR (400 MHz, CD_3_CN, 273 K): δ 10.76 (s, 1H, NH), 7.87 (d, 2H, ^3^*J*_H–P_ = 6.9, *o*-Ph), 7.50–7.13 (m, 14H, Ph + CPh), 6.97 (s, 2H, *H*-IPr), 3.58 and 3.22 (both d, 1H each, ^2^*J*_H–H_ = 13.5, C*H*_2_), 2.86 (sept, 2H, ^3^*J*_H–H_ = 6.6, C*H*CH_3_), 2.56 (sept, 2H, ^3^*J*_H–H_ = 6.7, C*H*CH_3_), 1.96 (s, 3H, N≡CC*H*_3_), 1.79 (d, 3H, ^3^*J*_H–H_ = 6.5, CHC*H*_3_), 1.70 (d, 3H, ^3^*J*_H–H_ = 6.4, CHC*H*_3_), 1.50 (m, 3H, PC*H*CH_3_), 1.38 (d, 3H, ^3^*J*_H–H_ = 6.4, CHC*H*_3_), 1.18 (d, 3H, ^3^*J*_H–H_ = 6.7, CHC*H*_3_), 1.16 (d, 3H, ^3^*J*_H–H_ = 6.7, CHC*H*_3_), 1.06 (d, 3H, ^3^*J*_H–H_ = 6.5, CHC*H*_3_), 0.81 (d, 3H, ^3^*J*_H–H_ = 6.5, CHC*H*_3_), 0.56 (q, 9H, ^2^*J*_H–P_ = 7.1, ^3^*J*_H–H_ = 6.0, PCHC*H*_3_), 0.50 (d, 3H, ^3^*J*_H–H_ = 6.5, CHC*H*_3_), 0.01 (br, 9H, PCHC*H*_3_), −17.95 (d, 1H, ^2^*J*_H–P_ = 25.9, Os-*H*). ^31^P{^1^H} NMR (121.4 MHz, CD_3_CN, 298 K): δ 24.7 (s). ^13^C{^1^H} NMR plus HMBC and HSQC (101 MHz, CD_3_CN, 273 K): δ 213.1 (d, ^2^*J*_C–P_ = 4.6, Os*C*), 180.1 (s, N*C*O), 169.2 (d, ^2^*J*_C–P_ = 57.5, N*C*N), 153.1 (s, C_*ipso*_-Ph), 147.6, 146.4, 146.3, 136.1, and 135.4 (all s, *C*_*ipso*_*-C*Ph + *C*_*o*_*-C*Ph), 139.6 (s, C_*ipso*_-CH_2_*Ph*), 130.9, 130.5, 129.4, and 128.9 (all s, Ph), 127.8, 126.8, and 126.4 (all s, CPh), 125.0 (s, *C*-IPr), 124.9, 124.1, and 123.6 (all s, Ph), 118.3 (s, N ≡ *C*-*C*H_3_), 39.1 (s, *C*H_2_Ph), 30.5, 29.7, 29.4, and 28.8 (all s, *C*HCH_3_), 26.4 (d, ^3^*J*_C–P_ = 26.1, P*C*HCH_3_), 26.0, 25.9, 25.4, 23.3, 23.0, 22.4, and 21.4 (all s, *C*HCH_3_), 18.4 and 18.5 (both s, PCH*C*H_3_).

### Preparation of OsH{κ^2^-*C,O*-[C(Ph)NC(CH_3_)O]}(IPr)(P^i^Pr_3_) (**5**)

The complex [OsH{κ^2^-*C,O*-[C(Ph)NHC(CH_3_)O]}(NCCH_3_)(IPr)(P^i^Pr_3_)]OTf (**2**; 100 mg, 0.09 mmol) was treated with potassium *tert*-butoxide (11 mg, 0.10 mmol) in THF (5 mL). After 5 min of stirring at room temperature, the brown solution was evaporated to dryness. The residue was treated with toluene. The resulting mixture was filtered over Celite. The dark solution was concentrated under reduced pressure. The addition of acetonitrile afforded a brown solid, which was decanted and washed with more acetonitrile (3 × 3 mL). Finally, the solid was dried *in vacuo*. Yield: 62 mg (78%). Anal. Calcd for C_45_H_66_N_3_OOsP: C, 60.99; H, 7.51; N, 4.74. Found: C, 61.23; H, 7.69; N, 4.95. MS (electrospray, *m*/*z*): C_45_H_67_N_3_OOsP [M + H], 888.4631; found, 888.4623. IR (cm^–1^): ν(Os–H) 2210 (m). ^1^H NMR (400 MHz, Tol-*d*_8_, 253 K): δ 8.54 (d, 2H, ^3^*J*_H–H_ = 7.4, *o*-Ph), 7.21–6.95 (m, 9H, Ph, CPh), 6.31 (s, 2H, H-IPr), 3.24 (br, 2H, C*H*CH_3_), 2.92 (m, 2H, C*H*CH_3_), 2.56 (s, 3H, O=CC*H*_3_), 1.77 (sept, 3H, ^3^*J*_H–H_ = 7.3, PC*H*CH_3_), 1.67 (d, 6H, ^3^*J*_H–H_ = 6.3, CHC*H*_3_), 1.26 (m, 6H, CHC*H*_3_), 1.13 (d, 6H, ^3^*J*_H–H_ = 6.6, CHC*H*_3_), 1.09 (d, 6H, ^3^*J*_H–H_ = 6.6, CHC*H*_3_), 0.79 (dd, 9H, ^2^*J*_H–P_ = 13.8, ^3^*J*_H–H_ = 7.1, PCHC*H*_3_), 0.56 (dd, 9H, ^2^*J*_H–P_ = 11.9, ^3^*J*_H–H_ = 7.1, PCHC*H*_3_), −12.73 (d, 1H, ^2^*J*_H–P_ = 20.0, Os-*H*). ^31^P{^1^H} NMR (121.4 MHz, Tol-*d*_*8*_, 298 K): δ 40.8 (s). ^13^C{^1^H} NMR plus HMBC and HSQC (101 MHz, Tol-*d*_8_, 253 K): δ 236.9 (Os*C*, inferred from the HMBC spectrum), 189.4 (br, N*C*O), 189.3 (br, *C*-IPr), 154.7 (s, C_*ipso*_-Ph), 146.5, 146.1, and 136.7 (all s, *C*_*ipso*_-CPh + *C*_*o*_-CPh), 129.4, 129.1, 128.2, 126.5, 123.8 and 123.7, (all s, Ph + CPh), 29.7, 28.8, 26.0, and 25.7 (all s, *C*HCH_3_), 25.3 (d, ^3^*J*_C–P_ = 22.9, P*C*HCH_3_), 23.3, 23.2, and 22.4 (all s, CH*C*H_3_), 18.6 and 18.5 (both s, PCH*C*H_3_).

### Preparation of OsH{κ^2^-*C,O*-[C(Ph)NC(CH_2_Ph)O]}(IPr)(P^i^Pr_3_) (**6**)

The complex [OsH{κ^2^-*C,O*-[C(Ph)NHC(CH_2_Ph)O]}(NCCH_3_)(IPr)(P^i^Pr_3_)]OTf (**4**; 100 mg, 0.08 mmol) was treated with potassium *tert*-butoxide (11 mg, 0.10 mmol) in THF (5 mL). After 5 min of stirring at room temperature, the brown solution was evaporated to dryness. The residue was treated with toluene. The resulting mixture was filtered over Celite. The dark solution was concentrated under reduced pressure. The addition of acetonitrile afforded a pale brown solid, which was decanted and washed with more acetonitrile (3 × 3 mL). Finally, the solid was dried *in vacuo*. Yield: 63 mg (78%). X-ray-quality crystals of **6** were formed by evaporation in pentane at 4 °C in a drybox. Anal. Calcd for C_51_H_70_N_3_OOsP: C, 63.65; H, 7.33; N, 4.37. Found: C, 63.64; H, 7.47; N, 4.48. MS (electrospray, *m*/*z*): C_51_H_71_N_3_OOsP [M + H], 964.4944; found, 964.4925. IR (cm^–1^): ν(Os–H) 2216 (w). ^1^H NMR (300 MHz, Tol-*d*_8_, 298 K): δ 9.02 (d, 2H, ^3^*J*_H–H_ = 7.2, *o*-Ph), 8.14 (d, 2H, ^3^*J*_H–H_ = 7.5, *o*-Ph), 7.74–7.43 (m, 12H, Ph, CPh), 6.96 (s, 2H, *H*-IPr), 4.83 and 4.51 (both d, 1H each, ^2^*J*_H–H_ = 13.0, C*H*_*2*_Ph), 3.88 (br, 2H, C*H*CH_3_), 3.39 (sept, 2H, ^3^*J*_H–H_ = 6.8, C*H*CH_3_), 2.10 (d, 6H, ^3^*J*_H–H_ = 6.7, CHC*H*_3_ + 3H, PC*H*CH_3_), 1.98 (br, 6H, CHC*H*_3_), 1.61 and 1.57 (both d, 12H, ^3^*J*_H–H_ = 6.1, CHC*H*_3_), 1.13 (dd, 9H, ^2^*J*_H–P_ = 13.8, ^3^*J*_H–H_ = 7.2, PCHC*H*_3_), 0.98 (dd, 9H, ^2^*J*_H–P_ = 12.2, ^3^*J*_H–H_ = 7.2, PCHC*H*_3_). ^1^H NMR (400 MHz, Tol-*d*_*8*_, 223 K, high field region): δ −15.35 (d, 1H, ^2^*J*_H–P_ = 23.9, Os-*H*). ^31^P{^1^H} NMR (121.4 MHz, Tol-*d*_8_, 298 K): δ 43.8 (s). ^13^C{^1^H} NMR plus HMBC and HSQC (75 MHz, Tol-*d*_8_, 298 K): 230.8 (br, Os*C*), 192.5 (d, ^2^*J*_C–P_ = 64.6, N*C*N), 188.7 (s, N*C*O), 154.3 (s, C_*ipso*_-Ph), 146.6 and 136.8 (s, C_*ipso*_-CPh + C_*o*_-CPh), 140.0 (s, C_*ipso*_-CH_2_*Ph*), 131.3, 130.2, 129.7, 128.0, 126.7, 126.5, 125.8, and 124.5 (all s, Ph + CPh), 124.1 and 124.0 (both s, *C*-IPr), 43.4 (s, *C*H_2_Ph), 29.0 and 28.9 (both s, *C*HCH_3_), 26.1, 25.9, 23.1, and 23.0 (all s, CH*C*H_3_), 25.0 (d, ^3^*J*_C–P_ = 22.5, P*C*HCH_3_), 18.9 and 18.8 (both s, PCH*C*H_3_).

### Preparation of [Os{η^3^-*C*_3_,κ^1^-*O*-[CH_2_C(Ph)C(Ph)NHC(CH_3_)O]}(NCCH_3_)_2_(IPr)]OTf (**7**)

Phenylacetylene (10.8 μL, 0.10 mmol) was added to a solution of [OsH{κ^2^-*C,O*-[C(Ph)NHC(CH_3_)O]}(NCCH_3_)(IPr)(P^i^Pr_3_)]OTf (**2**; 100 mg, 0.09 mmol) in 5 mL of acetonitrile. The purple mixture was stirred at room temperature for 5 days. After that, the resulting brown solution was filtered through Celite and the solvent was removed *in vacuo*. The addition of 5 mL of diethyl ether led to a yellow solid that was washed with more diethyl ether (3 × 3 mL) and dried *invacuo*. Yield: 84 mg (88%). X-ray-quality crystals were obtained from a dichloromethane–diethyl ether mixture by diffusion at 4 °C in a drybox. Anal. Calcd for C_49_H_58_F_3_N_5_O_4_OsS: C, 55.51; H, 5.51; N, 6.61; S, 3.02. Found: C, 55.22; H, 5.50; N, 6.47; S, 3.17. MS (electrospray, *m*/*z*): C_46_H_55_N_4_OOs [M – CH_3_CN], 871.3985; found, 871.3962. IR (cm^–1^): ν(N–H) 3229 (w); ν(NCO) 1594 (s). ^1^H NMR (300 MHz, CD_2_Cl_2_, 298 K): δ 8.50 (s, 1H, NH), 7.49–7.27 (6H, Ph + CPh), 7.21 (s, 2H, *H*-IPr), 7.12–6.94 (m, 6H, Ph + CPh), 6.85–6.72 (4H, Ph + CPh), 3.02 (sept, 2H, ^3^*J*_H–H_ = 16.8, C*H*CH_3_), 2.90 (sept, 2H, ^3^*J*_H–H_ = 6.8, C*H*CH_3_), 2.44 (s, 3H, N≡CC*H*_3_), 1.99 (s, 3H, O=CC*H*_3_), 1.77 (d, ^2^*J*_H–H_ = 5.9, 1H, Os-C*H*_*2*_), 1.35 (s, 3H, N≡CC*H*_3_), 1.28 (d, 6H, ^3^*J*_H–H_ = 6.8, CHC*H*_3_), 1.25 (d, 6H, ^3^*J*_H–H_ = 6.9, CHC*H*_3_), 1.18 (d, 6H, ^3^*J*_H–H_ = 6.8, CHC*H*_3_), 1.17 (d, 6H, ^3^*J*_H–H_ = 6.8, CHC*H*_3_), 1.06 (d, ^2^*J*_H–H_ = 5.9, 1H, Os-C*H*_*2*_). ^13^C{^1^H} NMR plus HMBC and HSQC (75 MHz, CD_2_Cl_2_, 298 K): δ 181.5 (s, N*C*O), 172.2 (s, N*C*N), 147.3, 146.8, 139.3, and 139.2 (all s, C_*ipso*_-CPh + C_*o*_-CPh, *C*_*ipso*_*-*Ph), 144.1 (s, *C*_*ipso*_*-C*Ph), 131.3, 130.6, 128.0, 127.4, 127.1, and 126.8 (all s, Ph, *C*Ph), 126.2 (s, *C*-IPr), 124.3, 124.1, and 124.0 (all s, Ph), 121.6 (s, N ≡ *C*CH_3_), 119.8 (s, N≡*C*CH_3_), 93.7 (s, N*C*Ph), 76.8 (s, Os*C*Ph), 30.8 (s, Os-*C*H_2_), 29.1 and 29.0 (both s, *C*HCH_3_), 26.4, 26.2, 23.1, and 23.0 (all s, CH*C*H_3_), 19.2 (s, O=CC*H*_3_), 5.8 (s, N≡C*C*H_3_), 2.9 (s, N≡C*C*H_3_).

### Preparation of [Os{η^3^-*C*_3_*,*κ^1^-*O*-[CH_2_C(Ph)C(Ph)NHC(CH_2_Ph)O]}(NCCH_3_)_2_(IPr)]OTf (**8**)

Phenylacetylene (10.1 μL, 0.09 mmol) was added to a solution of [OsH{κ^2^-*C,O*-[C(Ph)NHC(CH_2_Ph)O]}(NCCH_3_)(IPr)(P^i^Pr_3_)]OTf (**4**; 100 mg, 0.08 mmol) in 5 mL of acetonitrile. The purple mixture was stirred at room temperature for 24 h. After that, the resulting solution was filtered through Celite and the solvent was removed *in vacuo*. The addition of 5 mL of diethyl ether led to a yellow solid, which was washed with more diethyl ether (3 × 3 mL) and dried *invacuo*. Yield: 94 mg (96%). Anal. Calcd for C_55_H_62_F_3_N_5_O_4_OsS: C, 58.13; H, 5.50; N, 6.16; S, 2.82. Found: C, 57.81; H, 5.43; N, 6.36; S, 2.64. MS (electrospray, *m*/*z*): C_50_H_56_N_3_OOs [M – 2CH_3_CN], 906.4032; found, 906.4021. IR (cm^–1^): ν(NCO) 1594 (s). ^1^H NMR (300 MHz, CD_3_CN, 298 K): δ 7.64 (s, 1H, NH), 7.68–7.31 (11H, Ph + CPh + *H*-IPr), 7.17–6.72 (m, 10H, Ph + CPh), 6.47 (m, 2H, Ph), 3.55 (br, 2H, C*H*_*2*_Ph), 3.04 (sept, 2H, ^3^*J*_H–H_ = 6.7, C*H*CH_3_), 2.09 (sept, 2H, ^3^*J*_H–H_ = 6.8, C*H*CH_3_), 2.48 (s, 3H, N≡CC*H*_3_), 1.96 (s, 3H, N≡CC*H*_3_), 1.73 (d, ^3^*J*_H–H_ = 5.7, 1H, C*H*_*2*_), 1.30 (d, 6H, ^3^*J*_H–H_ = 6.8, CHC*H*_3_), 1.26 (d, 6H, ^3^*J*_H–H_ = 6.8, CHC*H*_3_), 1.18 (d, 6H, ^3^*J*_H–H_ = 6.8, CHC*H*_3_), 1.17 (d, 6H, ^3^*J*_H–H_ = 6.8, CHC*H*_3_), 1.10 (d, ^2^*J*_H–H_ = 5.7, 1H, Os-C*H*_*2*_). ^13^C{^1^H} NMR plus HMBC and HSQC (75 MHz, CD_3_CN, 298 K): δ 183.5 (s, N*C*O), 171.7 (s, N*C*N), 147.7 (s, *C*Ph), 147.1 (s, *C*Ph), 144.7, 139.8, 139.6, and 134.7 (all s, *C*_*ipso*_*-*Ph + *C*_*ipso*_*-*CH_2_*Ph*), 131.0, 130.77, 129.8, and 129.9 (all s, *C*Ph), 128.4, 127.94, 127.77, 127.04, and 126.82 (all s, Ph), 126.6 (*C*-IPr), 124.3, 124.2, and 124.0 (all s, *C*Ph), 121.4 (s, N ≡ *C*CH_3_), 118.0 (s, N≡CH_3_), 92.9 (s, N*C*Ph), 75.3 (s, OsCPh), 39.8 (s, *C*H_2_Ph), 30.5 (s, Os-*C*H_2_), 29.2 and 29.1 (both s, *C*HCH_3_), 26.1, 25.8, 22.9, and 22.8 (all s, CH*C*H_3_), 5.3 (s, N≡C*C*H_3_).

### Preparation of OsH_2_(C≡CPh){κ^2^-*C,O*-[C(Ph)NC(CH_3_)O]}(IPr)(P^i^Pr_3_) (**9**)

Phenylacetylene (14 μL, 0.12 mmol) was added to a solution of OsH{κ^2^-*C,O*-[C(Ph)NC(CH_3_)O]}(IPr)(P^i^Pr_3_) (**5**; 100 mg, 0.11 mmol) in 5 mL of toluene. The resulting orange solution was stirred for 5 min. After this time, the mixture was concentrated *in vacuo*. The subsequent addition of 3 mL of acetonitrile afforded an orange solid, which was washed with acetonitrile (3 × 3 mL) and was dried *in vacuo*. Yield: 64 mg (54%). Anal. Calcd for C_53_H_72_N_3_OOsP: C, 64.41; H, 7.34; N, 4.25. Found: C, 64.52; H, 7.73; N, 4.46. MS (electrospray, *m*/*z*): C_53_H_71_N_3_OOsP [M – H], 988.4944; found, 988.4922. IR (cm^–1^): ν(Os–H_2_) 2867 (w); ν(C≡C) 2100 (s). ^1^H NMR (300 MHz, Tol-*d*_8_, 298 K): δ 8.69 (d, 2H, ^3^*J*_H–H_ = 7.6, *o*-Ph), 7.41–6.98 (m, 14H, Ph + CPh), 6.58 (br, 1H, *H*-IPr), 6.13 (br, 1H, *H*-IPr), 4.10 (br, 2H, C*H*CH_3_), 2.09 (m, 3H, PC*H*CH_3_), 2.98 (br, 1H, C*H*CH_3_), 2.32 (br, 1H, C*H*CH_3_), 2.03 (s, 3H, C*H*_3_), 1.69–1.08 (24H, CHC*H*_3_), 0.90 (dd, 9H, ^2^*J*_H–P_ = 13.0, ^3^*J*_H–H_ = 7.2, PC*H*_3_), 0.77 (dd, 9H, ^2^*J*_H–P_ = 12.2, ^3^*J*_H–H_ = 7.2, PC*H*_3_), −8.90 (d, 2H, ^2^*J*_H–P_ = 11.6, Os-*H*_*2*_). *T*_1_(min) (ms, Os-H_2_, 300 MHz, toluene-*d*_8_, 253 K): 47 ± 4 (−8.89 ppm). ^31^P{^1^H} NMR (121.4 MHz, Tol-*d*_8_, 298 K): δ 5.4 (s). ^13^C{^1^H} NMR plus HMBC and HSQC (75 MHz, Tol-*d*_8_, 298 K): δ 273.7 (d, ^2^*J*_C–P_ = 0.1, Os*C*), 193.2 (s, N*C*O), 167.8 (d, ^2^*J*_C–P_ = 77.6, N*C*N), 154.0 (s, *C*_*ipso*_*-*Ph), 148.3, 146.2, and 146.0 (all s, *C*_*ipso*_*-C*Ph + C_*o*_-CPh), 131.5 (s, *C*_*ipso*_-C≡C-*Ph*), 132.6, 131.0, 129.7, 128.1, 127.5, and 126.8 (all s, Ph + CPh), 124.4 (s, *C*-IPr), 123.7, 123.5, and 123.1 (all s, *C*Ph), 116.8 (s, ≡*C*-Ph), 108.8 (d, ^2^*J*_C–P_ = 14.7, Os-*C*≡), 29.3 and 29.0 (both s, *C*HCH_3_), 26.4 (d, ^3^*J*_H–H_ = 24.1, P*C*HCH_3_), 24.4, 24.1, 23.3, and 21.7 (all s, CH*C*H_3_), 19.9 and 19.2 (both s, PCH*C*H_3_).

### Preparation of OsH_2_(C≡CPh){κ^2^-*C,O*-[C(Ph)NC(CH_2_Ph)O]}(IPr)(P^i^Pr_3_) (**10**)

Phenylacetylene (13 μL, 0.11 mmol) was added to a solution of OsH{κ^2^-*C,O*-[C(Ph)NC(CH_2_Ph)O]}(IPr)(P^i^Pr_3_) (**6**; 100 mg, 0.10 mmol) in 5 mL of toluene. After 5 min of stirring at room temperature, the resulting orange solution was concentrated *in vacuo*. The subsequent addition of 3 mL of acetonitrile afforded an orange solid, which was washed with acetonitrile (3 × 3 mL) and was dried *in vacuo*. Yield: 58 mg (49%). Anal. Calcd for C_59_H_76_N_3_OOsP: C, 66.57; H, 7.20 N, 3.95. Found: C, 66.78; H, 7.29; N, 4.07. MS (electrospray, *m*/*z*): C_59_H_75_N_3_OOsP [M – H], 1064.5257; found, 1064.5265. IR (cm^–1^): ν(C≡C) 1658 (w). ^1^H NMR (300 MHz, Tol-*d*_8_, 298 K): δ 8.71 (dd, 2H, ^2^*J*_H–P_ = 8.0, ^3^*J*_H–H_ = 2.2, *o*-Ph), 7.47 (d, 2H, ^3^*J*_H–H_ = 7.2, Ph), 7.44–6.91 (m, 17H, Ph + CPh), 6.59 (br, 1H, *H*-IPr), 6.50 (br, 1H, *H*-IPr), 4.24 (sept, 1H, ^3^*J*_H–H_ = 6.8, C*H*CH_3_), 4.10 (sept, 1H, ^3^*J*_H–H_ = 6.6, C*H*CH_3_), 3.73 (d, 1H, ^2^*J*_H–H_ = 12.7, C*H*_*2*_Ph), 3.12 (d, 1H, ^2^*J*_H–H_ = 12.8, C*H*_*2*_Ph), 2.91 (sept, 1H, ^3^*J*_H–H_ = 6.5, C*H*CH_3_), 2.34 (sept, 1H, ^3^*J*_H–H_ = 6.4, C*H*CH_3_), 1.89 (m, 3H, PC*H*CH_3_), 1.81 (d, 3H, *J*_H–H_ = 6.4, CHC*H*_3_), 1.43 (d, 3H, ^3^*J*_H–H_ = 6.1, CHC*H*_3_), 1.31 (d, 3H, ^3^*J*_H–H_ = 7.0, CHC*H*_3_), 1.28 (d, 3H, ^3^*J*_H–H_ = 7.0, CHC*H*_3_), 1.18 (d, 3H, ^3^*J*_H–H_ = 6.5, CHC*H*_3_), 1.14 (d, 3H, ^3^*J*_H–H_ = 6.8, CHC*H*_3_), 0.85 (d, 3H, ^3^*J*_H–H_ = 6.5, CHC*H*_3_), 0.70 (dd, 9H, ^2^*J*_H–P_ = 13.0, ^3^*J*_H–P_ = 7.2, PCHC*H*_3_ + 3H CHC*H*_3_), 0.56 (dd, 9H, ^2^*J*_H–P_ = 12.6, ^3^*J*_H–H_ = 7.3, PCHC*H*_3_), −8.97 (d, 2H, ^2^*J*_H–H_ = 11.8, Os-*H*_*2*_). *T*_1_(min) (ms, Os-H_2_, 300 MHz, toluene-*d*_8_, 273 K): 44 ± 4 (−8.97 ppm). ^31^P{^1^H} NMR (121.4 MHz, Tol-*d*_*8*_, 298 K): δ 6.7 (s). ^13^C{^1^H} NMR plus HMBC and HSQC (75 MHz, Tol-*d*_8_, 298 K): δ 274.8 (d, ^2^*J*_C–P_ = 5.0, Os*C*), 192.6 (s, N*C*O), 166.7 (d, ^2^*J*_C–P_ = 76.7, N*C*N), 154.0 (s, *C*_*ipso*_*-*Ph), 148.8, 148.6, 146.6, and 146.5 (all s, *C*_*ipso*_*-C*Ph + C_*o*_-CPh), 140.8 (s, *C*_*ipso*_*-*Ph), 138.2 (s, *C*_*ipso*_*-*CH_2_*Ph*), 131.8 (d, ^3^*J*_C–H_ = 1.2, *C*_*ipso*_-C≡C-*Ph*), 133.1, 131.5, 131,4, 131.0, 130.9, 130.3, 130.2, 130.1, 128.5, 128.4, 127.9, and 126.6 (all s, Ph), 125.4 (s, *C*-IPr), 125.2, 124.9, 123.9, and 123.6 (all s, CPh), 117.5 (d, ^3^*J*_C–P_ = 2.0, ≡*C*-Ph), 108.9 (d, ^2^*J*_C–P_ = 14.9, Os-*C*≡), 42.8 (s, *C*H_2_Ph), 29.8, 29.6, 29.3, and 28.9 (all s, *C*HCH_3_), 27.6, 24.1, 23.6, and 23.5 (all s, CH*C*H_3_), 26.4 (d, ^3^*J*_H–H_ = 24.7, P*C*HCH_3_), 20.3 and 19.5 (both s, PCH*C*H_3_).

### Preparation of Os(C≡CPh){κ^2^-*C,O*-[C(Ph)NC(CH_3_)O]}(IPr)(P^i^Pr_3_) (**11**)

Phenylacetylene (14 μL, 0.12 mmol) was added to a solution of OsH{κ^2^-*C,O*-[C(Ph)NC(CH_3_)O]}(IPr)(P^i^Pr_3_) (**5**; 100 mg, 0.11 mmol) in 5 mL of toluene. The mixture was heated at 70 °C and was stirred for 18 h. After that, the solvent was evaporated to dryness. The addition of 3 mL of acetonitrile led to a purple solid, which was washed with acetonitrile (3 × 3 mL) and was dried *in vacuo*. Yield: 69 mg (63%). X-ray-quality crystals of **11** were obtained by evaporation in pentane at 4 °C in a drybox. Anal. Calcd for C_53_H_70_N_3_OOsP: C, 64.54; H, 7.15 N, 4.26. Found: C, 64.74; H, 7.22; N, 4.38. MS (electrospray, *m*/*z*): C_53_H_71_N_3_OOsP [M + H], 988.4944; found, 988.4967. IR (cm^–1^): ν(C≡C) 2038 (s). ^1^H NMR (400 MHz, Tol-*d*_8_, 263 K): δ 7.40 (d, 2H, ^3^*J*_H–H_ = 7.4, *o*-Ph), 7.22–6.89 (m, 12H, Ph + CPh), 6.67–6.58 (m, 2H, Ph), 6.34 (s, 2H, *H*-IPr), 3.84 (br, 2H, C*H*CH_3_), 3.36 (br, 2H, C*H*CH_3_), 2.09 (m, 3H, PC*H*CH_3_), 2.65 (s, 3H, C*H*_3_), 1.68, 1.52, 1.45, 1.40, and 1.07 (all d, 3H each, ^3^*J*_H–H_ = 4.0, CHC*H*_3_), 1.00 (br, 6H, CHC*H*_3_), 0.76 (dd, 9H, PCHC*H*_3_ + 3H, CHC*H*_3_), 0.53 (dd, 9H, ^2^*J*_H–P_ = 12.7, ^3^*J*_H–H_ = 7.2, PCHC*H*_3_). ^31^P{^1^H} NMR (121.4 MHz, Tol-*d*_8_, 298 K): δ 27.2 (s). ^13^C{^1^H} NMR plus HMBC and HSQC (101 MHz, Tol-*d*_8_, 263 K): δ 243.5 (s, Os*C*), 191.0 (s, N*C*O), 186.8 (d, ^2^*J*_C–P_ = 71.3, N*C*N), 150.5 (s, *C*_*ipso*_*-*Ph), 147.6, 146.7, 145.2, 144.3, 137.0, and 135.7 (all s, *C*_*ipso*_*-C*Ph + C_*o*_-CPh), 130.6 (s, *C*_*ipso*_-C≡*C*-*Ph*), 132.5, 130.4, 129.5, 129.1, 128.3, and 127.8 (all s, Ph), 126.8 (s, ≡*C*-Ph), 126.7, 126.2, 124.7, 124.5, 124.2, and 123.6 (all s, *C*Ph), 123.1 (s, *C*-IPr), 118.8 (d, ^2^*J*_C–P_ = 11.2, Os-*C*≡), 29.0, 28.9, 28.8, and 28.7 (all s, *C*HCH_3_), 27.0, 26.8, and 26.4 (all s, CH*C*H_3_), 23.9 (d, ^3^*J*_H–H_ = 17.3, P*C*HCH_3_), 23.1, 22.4, and 22.2 (all s, PCH*C*H_3_), 19.7 (O=C-*C*H_3_).

### Preparation of Os(C≡CPh){κ^2^-*C,O*-[C(Ph)NC(CH_2_Ph)O]}(IPr)(P^i^Pr_3_) (**12**)

Phenylacetylene (13 μL, 0.11 mmol) was added to a solution of OsH{κ^2^-*C,O*-[C(Ph)NC(CH_2_Ph)O]}(IPr)(P^i^Pr_3_) (**6**; 100 mg, 0.10 mmol) in 5 mL of toluene. The mixture was heated at 70 °C and was stirred for 18 h. After that, the solvent was evaporated to dryness. The addition of 3 mL of acetonitrile led to a purple solid, which was washed with acetonitrile (3 × 3 mL) and was dried *in vacuo*. Yield: 68 mg (64%). Anal. Calcd for C_59_H_74_N_3_OOsP: C, 66.70; H, 7.02 N, 3.96. Found: C, 66.89; H, 7.09; N, 4.08. MS (electrospray, *m*/*z*): C_59_H_75_N_3_OOsP [M + H], 1064.5257; found, 1064.5206. IR (cm^–1^): ν(C≡C) 1590 (m). ^1^H NMR (300 MHz, Tol-*d*_8_, 263 K): δ 7.62 (d, 2H, ^3^*J*_H–H_ = 7.4, *o*-Ph), 7.48 (d, 2H, ^3^*J*_H–H_ = 7.9, *o*-Ph), 7.33–6.91 (m, 11H, Ph + CPh), 6.78–6.68 (m, 3H, Ph), 6.76 (t, 2H, ^3^*J*_H–H_ = 6.5, CPh), 6.69 (d, 1H, ^3^*J*_H–H_ = 7.5, CPh), 6.46 (d, 2H, ^3^*J*_H–H_ = 7.3, *H*-IPr), 4.73 (d, 1H, ^2^*J*_H–H_ = 11.5, C*H*_*2*_Ph), 4.04 (d, 1H, ^2^*J*_H–H_ = 11.5, C*H*_*2*_Ph), 3.91 (sept, 2H, ^3^*J*_H–H_ = 6.3, C*H*CH_3_), 3.57 (sept, 1H, ^3^*J*_H–H_ = 6.5, C*H*CH_3_), 2.25 (sept, 1H, ^3^*J*_H–H_ = 6.5, C*H*CH_3_), 1.90 (d, 3H, ^3^*J*_H–H_ = 6.3, CHC*H*_3_), 1.84 (m, 3H, PC*H*CH_3_), 1.61 (t, 6H, ^3^*J*_H–H_ = 6.8, CHC*H*_3_), 1.44 (d, 3H, ^3^*J*_H–H_ = 6.5, CHC*H*_3_), 1.19 (d, 3H, ^3^*J*_H–H_ = 6.8, CHC*H*_3_), 1.12 (d, 3H, ^3^*J*_H–H_ = 6.6, CHC*H*_3_), 1.11 (d, 3H, ^3^*J*_H–H_ = 6.6, CHC*H*_3_), 0.88 (d, 3H, ^3^*J*_H–H_ = 6.6, CHC*H*_3_), 0.70 (dd, 9H, ^2^*J*_H–P_ = 12.8, ^3^*J*_H–H_ = 7.2, PCHC*H*_3_), 0.40 (dd, 9H, ^2^*J*_H–P_ = 12.9, ^3^*J*_H–H_ = 7.2, PCHC*H*_3_). ^31^P{^1^H} NMR (121.4 MHz, Tol-*d*_*8*_, 298 K): δ 28.1 (s). ^13^C{^1^H} NMR plus HMBC and HSQC (75 MHz, Tol-*d*_8_, 263 K): δ 243.2 (s, Os*C*), 190.9 (s, N*C*O), 186.8 (d, ^2^*J*_C–P_ = 70.3, N*C*N), 150.5 (s, *C*_*ipso*_*-*Ph), 147.5, 146.8, 145.3, and 144.4 (all s, *C*_*ipso*_*-C*Ph + C_*o*_-CPh), 138.8 (s, *C*_*ipso*_*-*CH_2_*Ph*), 132.7 (s, *C*_*ipso*_-C≡*C*-*Ph*), 129.8, 128.3, 127.8, 126.7, and 126.4 (all s, Ph + CPh), 125.9 (s, ≡*C*-Ph), 124.3 (s, *C*-IPr), 123.7 and 123.1 (both s, Ph), 118.0 (d, ^2^*J*_C–P_ = 11.3, Os-*C*≡), 42.0 (s, *C*H_2_Ph), 29.0 and 28.9 (both s, *C*HCH_3_), 27.0 and 26.3 (both s, CH*C*H_3_), 23.3 (d, ^1^*J*_H–H_ = 23.0, P*C*HCH_3_), 19.7 and 19.6 (both s, PCH*C*H_3_).

## Supporting Information Available

General information, structural analysis of complexes **2**, **6**, **7**, and **11**, computational data, and NMR spectra (PDF)
Cartesian coordinates of computed complexes (XYZ)

## Supplementary Material

om1c00621_si_001.pdf

om1c00621_si_002.xyz

## Accession Codes

CCDC 2117089–2117092 contain the supplementary crystallographic data for this paper. These data can be obtained free of charge via www.ccdc.cam.ac.uk/data_request/cif, or by emailing data_request@ccdc.cam.ac.uk, or by contacting The Cambridge Crystallographic Data Centre, 12 Union Road, Cambridge CB2 1EZ, UK; fax: +44 1223 336033.
