# Supplementary material for: Dissimilarity in the Chemical Behavior of Osmaoxazolium Salts and Osmaoxazoles: Two Different Aromatic Metalladiheterocycles
Source: Organometallics. 2021 Dec 14;40(24):4150–62. doi: 10.1021/acs.organomet.1c00621 (PMC8895684; doi:10.1021/acs.organomet.1c00621)
Supplement: Supplementary file 1 — om1c00621_si_001.pdf [file om1c00621_si_001.pdf]

SUPPORTING INFORMATION

Dissimilarity in the Chemical Behavior of Osmaoxazolium Salts and Osmaoxazoles: Two Different Aromatic Metalladiheterocycles

María L. Buil, Miguel A. Esteruelas,* Enrique Oñate, and Nieves R. Picazo.

Departamento de Química Inorgánica, Instituto de Síntesis Química y Catálisis Homogénea (ISQCH), Centro de Innovación en Química Avanzada (ORFEO-CINQA), Universidad de Zaragoza-CSIC, 50009 Zaragoza, Spain

* email: maester@unizar.es

Contents:

- Experimental Details. **S1**

- Structural Analysis of Complexes 2, 6, 7 and 11. **S1**

- NMR Spectra. **S5**

- Computational Details. **S23**

- Energies of Optimized Structures. **S23**

- Calculated HOMO and LUMO of complexes 2 and 6. **S25**

- References. **S26**

- **Experimental details.**

General information: All reactions were carried out with exclusion of air using Schlenk-tube techniques or in a drybox. Diethyl ether, pentane, acetonitrile, dichloromethane and toluene were obtained oxygen- and water-free from an MBraun solvent purification apparatus, while tetrahydrofuran was dried and distilled under argon prior to use. ^{1}H, ^{13}C{^{1}H}, and ^{31}P{^{1}H} NMR spectra were recorded on Bruker 300 ARX, Bruker Advance 300 MHz or Bruker Advance 400MHz. Coupling constants *J* are given in hertz. Attenuated total reflection infrared spectra (ATR-IR) of solid samples were run on a PerkinElmer Spectrum 100 FT-IR spectrometer. Elemental analyses were carried out in a PerkinElmer 2400 CHNS/O analyzer. High-resolution electrospray mass spectra were acquired using a MicroTOF-Q hybrid quadrupole time-of-flight spectrometer (Bruker Daltonics, Bremen, Germany).

- **Structural Analysis of Complexes 2, 6, 7 and 11.**

X-ray data were collected for the complex on a Bruker APEX CCD (**6, 7**), DUO CCD (**2**) and D8 Venture (**11**) diffractometers (Mo radiation, λ = 0.71073 Å). The crystals were cooled with a nitrogen flow from Oxford Cryosystems systems. Data were corrected for absorption by using a multiscan method applied with the SADABS program.[1] The structures were solved by Patterson or direct methods and refined by full-matrix least squares on F^2 with SHELXL2016,[2] including isotropic and subsequently anisotropic displacement parameters. The hydrogen atoms were observed in the last Fourier Maps or calculated, and refined freely or using a restricted riding model. The hydride ligands were located in the last Fourier Maps and refined with restrained osmium-hydride distance (1.59 Å).

Crystal data for **2**: $C_{47}H_{70}N_4OOsP$ x CF_3O_3S x C_4H_8O, M_W 1149.41, red, irregular block (0.300 x 0.136 x 0.049 mm^3), monoclinic, space group $P2_1/c$, *a*: 12.714(2) Å, *b*: 20.553(3) Å, *c*: 20.600(3) Å, *β*: 95.016(2)°, V = 5362.2(14) $Å^3$, Z = 4, Z' = 1, D_{calc}: 1.424 g cm^{-3}, F(000): 2368, T = 120(2) K, μ 2.505 mm^{-1}. 41927 measured reflections (2θ: 3-57°, ω scans 0.3°), 10545 unique (R_{int} = 0.0829); min./max. transm. Factors 0.649/0.862. Final agreement factors were R^1 = 0.0479 (10545 observed reflections, I > 2σ(I)) and wR^2 = 0.1277; data/restraints/parameters 10545/1/626; GoF = 1.014. Largest peak and hole 4.853 (close to osmiun atoms) and -2.022 e/ $Å^3$.

Crystal data for **6**: $C_{51}H_{70}N_3OOsP$, M_W 962.27, red, irregular block (0.228 x 0.191 x 0.133 mm^3), triclinic, space group P-1, *a*: 11.7494(6) Å, *b*: 12.2666(6) Å, *c*: 16.2636(8) Å, *α*: 93.4050(10) °, *β*: 91.7590(10) °, *γ*: 98.0260(10) °, V = 2315.1(2) $Å^3$, Z = 2, Z' = 1, D_{calc}: 1.380 g cm^{-3}, F(000): 992, T = 100(2) K, μ 2.828 mm^{-1}. 27754 measured reflections (2θ: 3-57°, ω scans 0.3°), 10060 unique (R_{int} = 0.0255); min./max. transm. Factors 0.725/0.862. Final agreement factors were R^1 = 0.0247 (10060 observed reflections, I > 2σ(I)) and wR^2 = 0.0616; data/restraints/parameters 10937/1/ 531; GoF = 1.049. Largest peak and hole 2.291 (close to osmiun atoms) and -1.271 e/ $Å^3$.

Crystal data for **7**: $C_{48}H_{58}N_5OOs$ x CF_3O_3S x 1.5(C_4H_8O), C_5H_{12}, M_W 1204.49, yellow, irregular block (0.294 x 0.174 x 0.038 mm^3), orthorhombic, space group $P2_12_12_1$, *a*: 11.7953(10) Å, *b*: 18.1189(16) Å, *c*: 27.818(2) Å, V = 5945.2(9) $Å^3$, Z = 4, Z' = 1, D_{calc}: 1.346 g cm^{-3}, F(000): 2476, T = 100(2) K, μ 2.239 mm^{-1}. 69890 measured reflections (2θ: 3-57°, ω scans 0.3°), 14460 unique (R_{int} = 0.0561); min./max. transm. Factors 0.615/0.862. Final agreement factors were R^1 = 0.0370 (12785 observed reflections, I > 2σ(I)) and wR^2 = 0.0839; data/restraints/parameters 14460/ 37/ 650; GoF = 1.091. Largest peak and hole 0.858 (close to osmiun atoms) and -1.304 e/ $Å^3$.

Crystal data for **11**: $C_{53}H_{70}N_3OOsP$ x C_5H_{12}, M_W 1022.36, yellow, irregular block (0.185 x 0.033 x 0.030 mm^3), monoclinic, space group $P2_1/c$, *a*: 11.7850(4) Å, *b*: 19.1164(7) Å, *c*: 23.5826(8) Å, *β*: 97.2686(15) °, V = 5270.2(3) $Å^3$, Z = 4, Z' = 1, D_{calc}: 1.289 g cm^{-3}, F(000): 2116, T = 100(2) K, μ 2.489 mm^{-1}. 390157 measured reflections (2θ: 3-57°, ω scans 0.3°), 16109 unique (R_{int} = 0.0613); min./max. transm. Factors 0.779/0.862. Final agreement factors were R^1 = 0.0345 (15026 observed reflections, I > 2σ(I)) and wR^2 = 0.0898; data/restraints/parameters 16109/2/565; GoF = 1.126. Largest peak and hole 2.827 (close to osmium atoms) and -3.058 e/ $Å^3$.

- **NMR Spectra.**

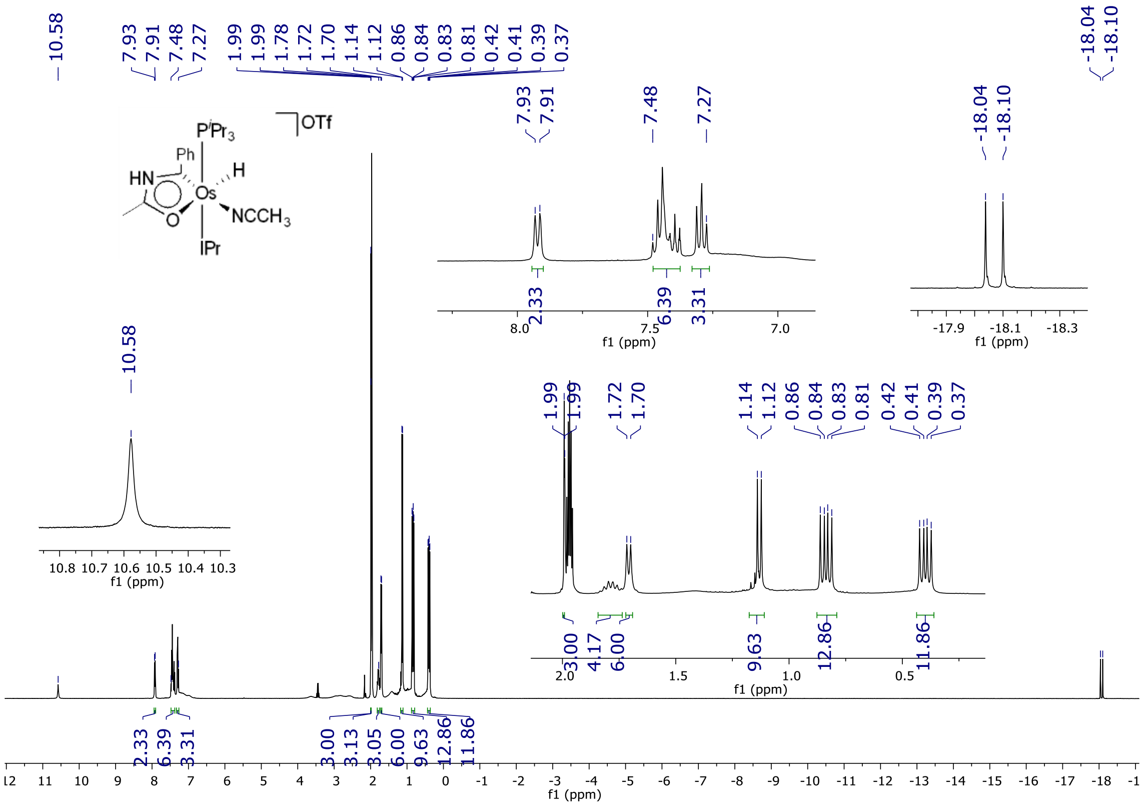

Figure S1. ^{1}H NMR spectrum (400 MHz, CD_3CN, 298 K) of compound **2**.

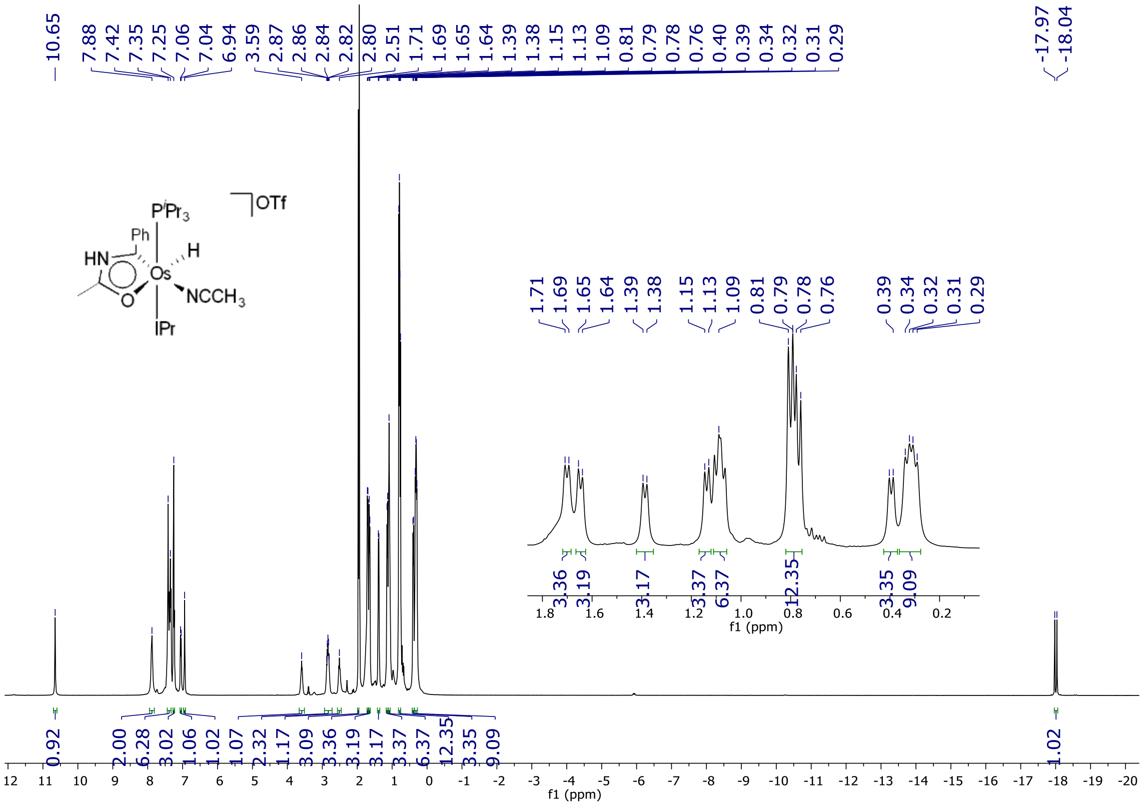

Figure S2. ^{1}H NMR spectrum (400 MHz, CD_3CN, 263 K) of compound **2**.

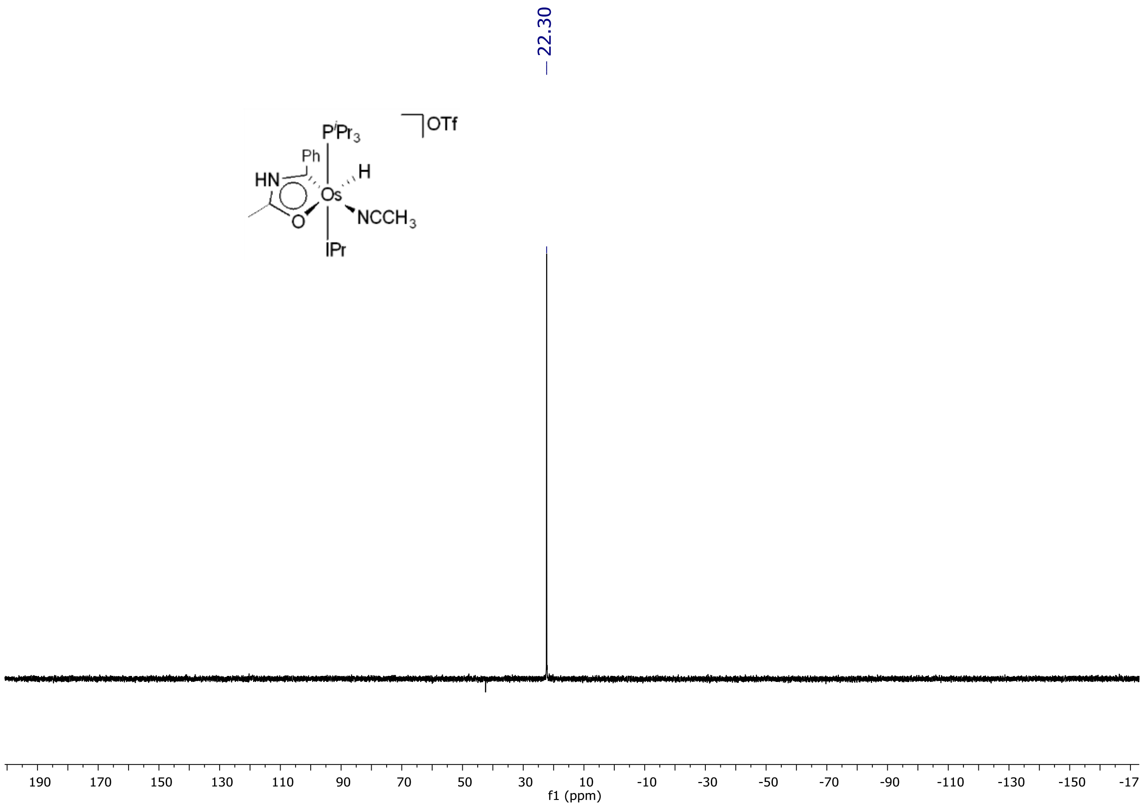

Figure S3. $^{31}P\{^1H\}$ spectrum (121 MHz, CD_3CN, 298 K) of compound **2**.

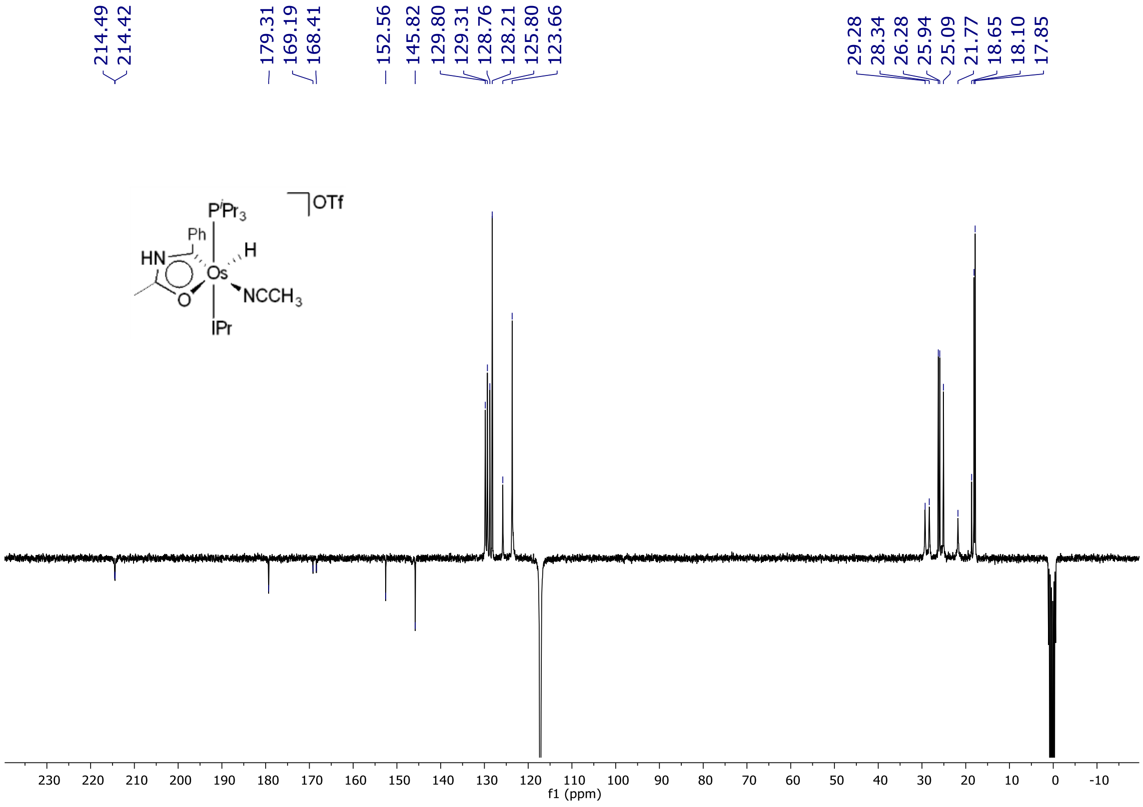

Figure S4. $^{13}C\{^1H\}$ spectrum (75 MHz, CD_3CN, 298 K) of compound **2**.

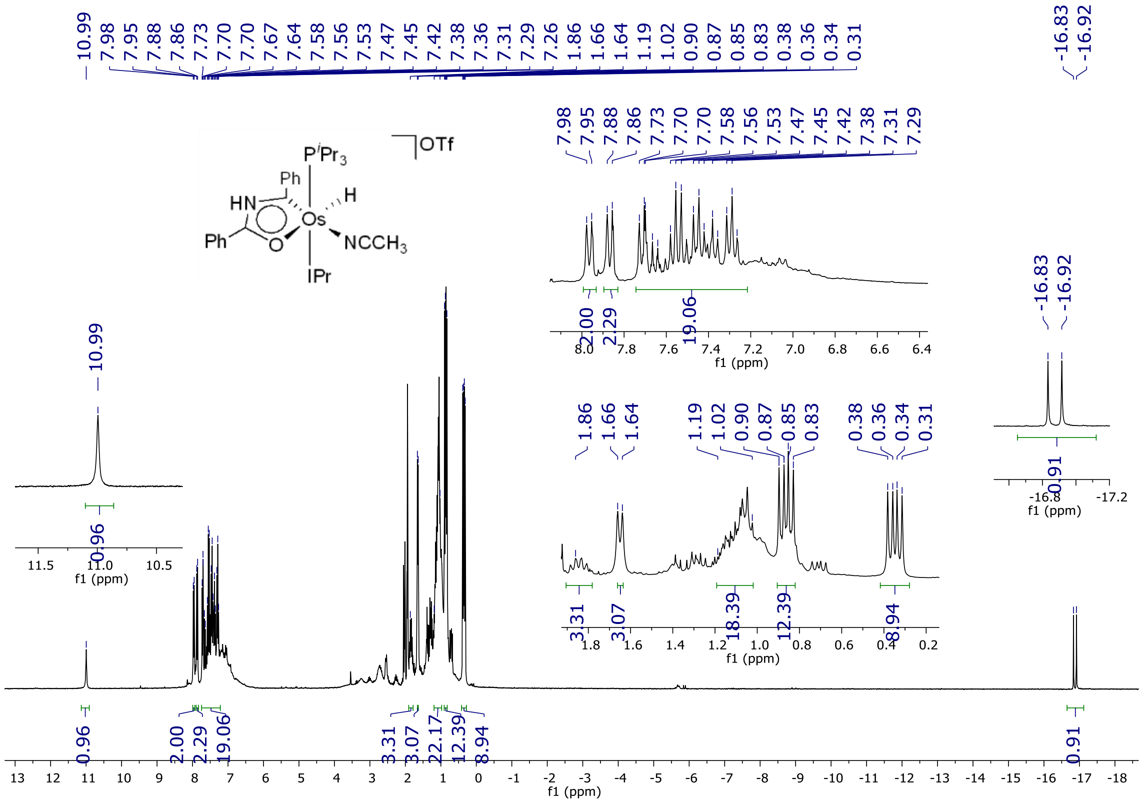

Figure S5. ^{1}H NMR spectrum (300 MHz, CD_3CN, 298 K) of compound **3**.

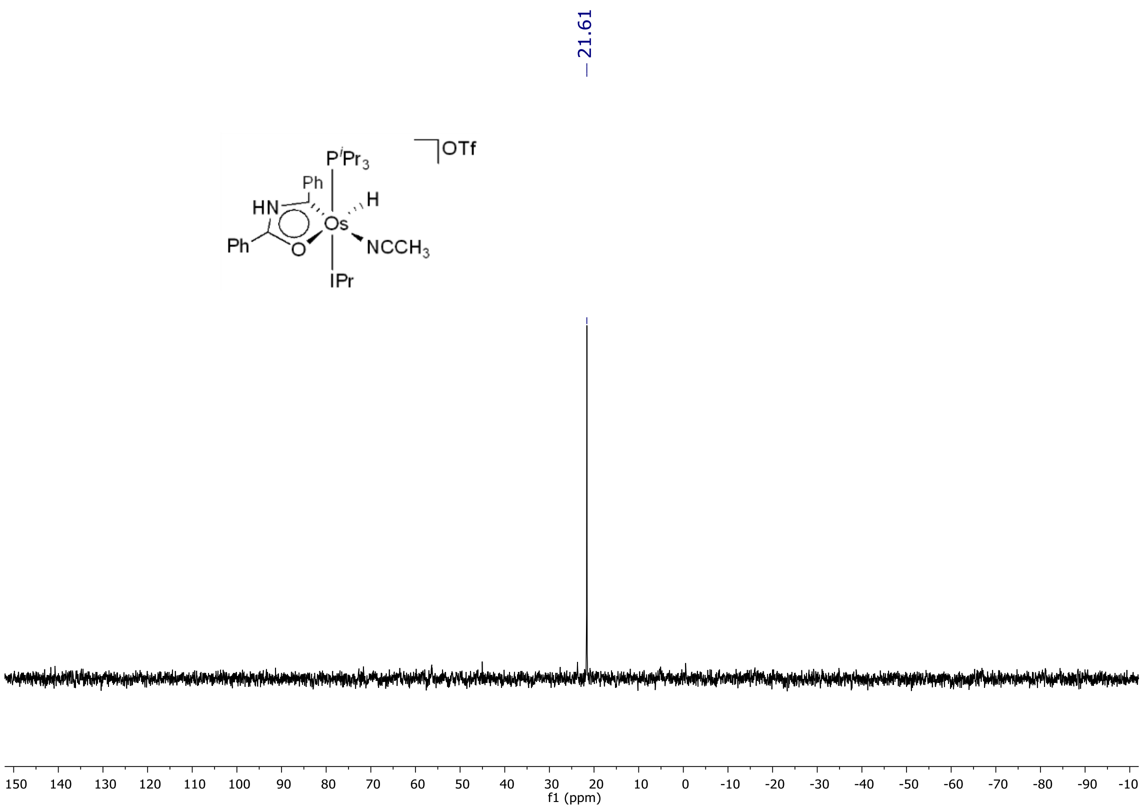

Figure S6. $^{31}P\{^{1}H\}$ spectrum (121 MHz, CD_3CN, 298 K) of compound **3**.

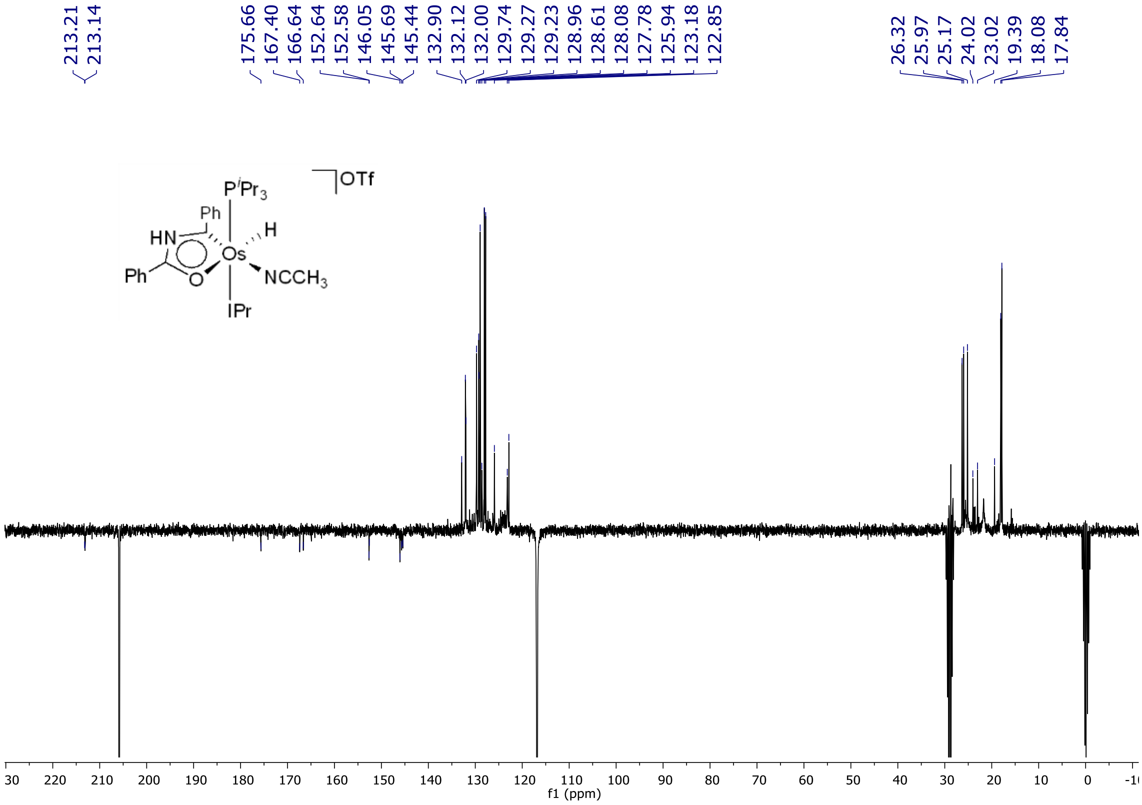

Figure S7. $^{13}C\{^1H\}$ spectrum (75 MHz, CD_3CN, 298 K) of compound **3**.

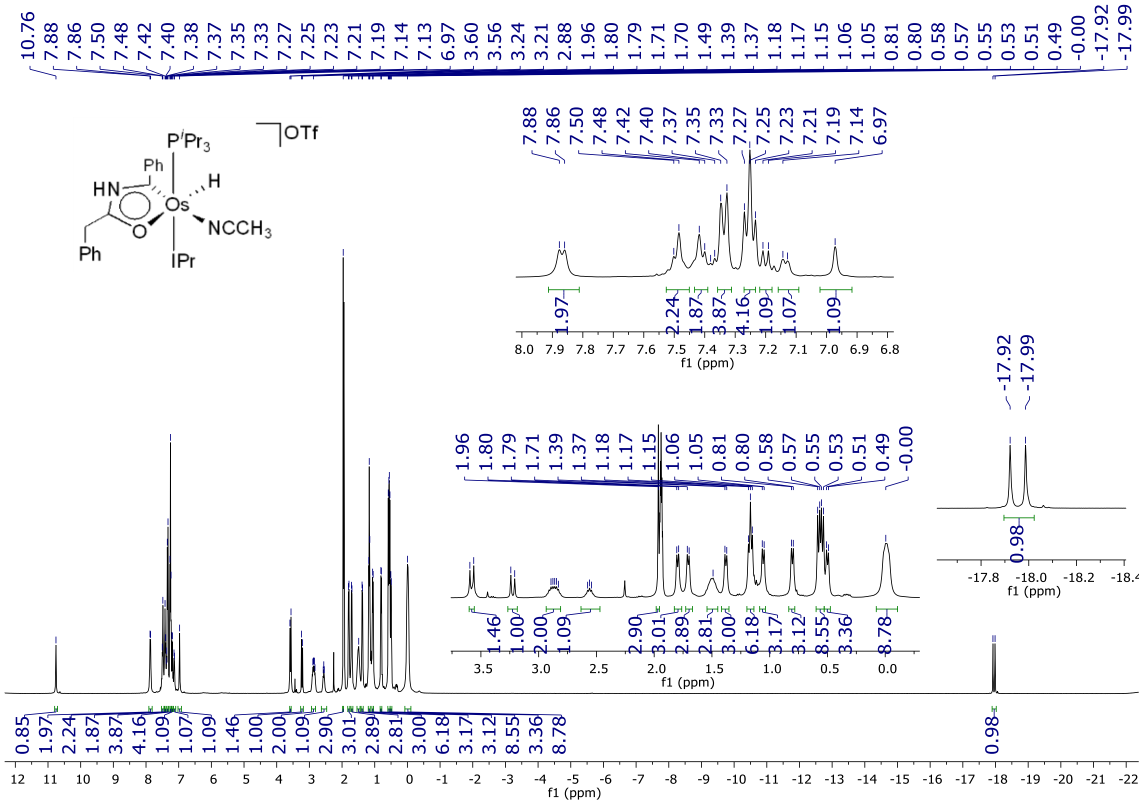

Figure S8. ^1H NMR spectrum (400 MHz, CD_3CN, 273 K) of compound **4**.

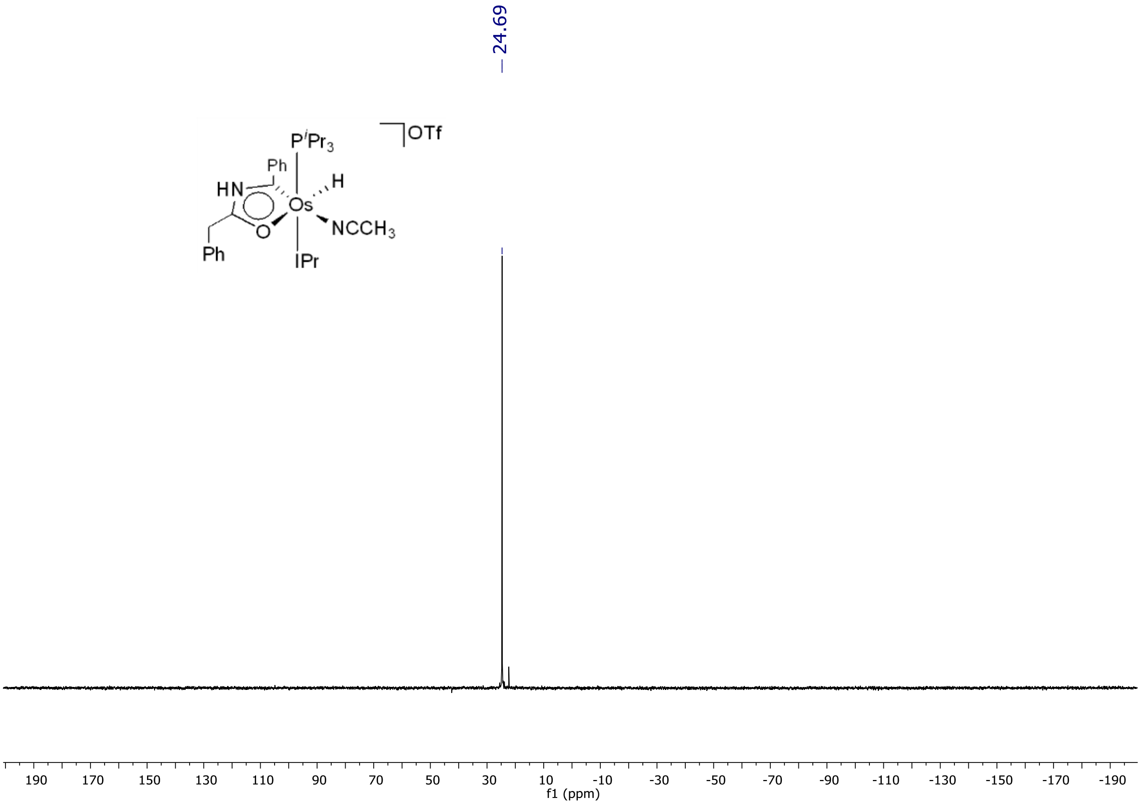

Figure S9. $^{31}P\{^{1}H\}$ spectrum (121 MHz, CD_3CN, 298 K) of compound **4**.

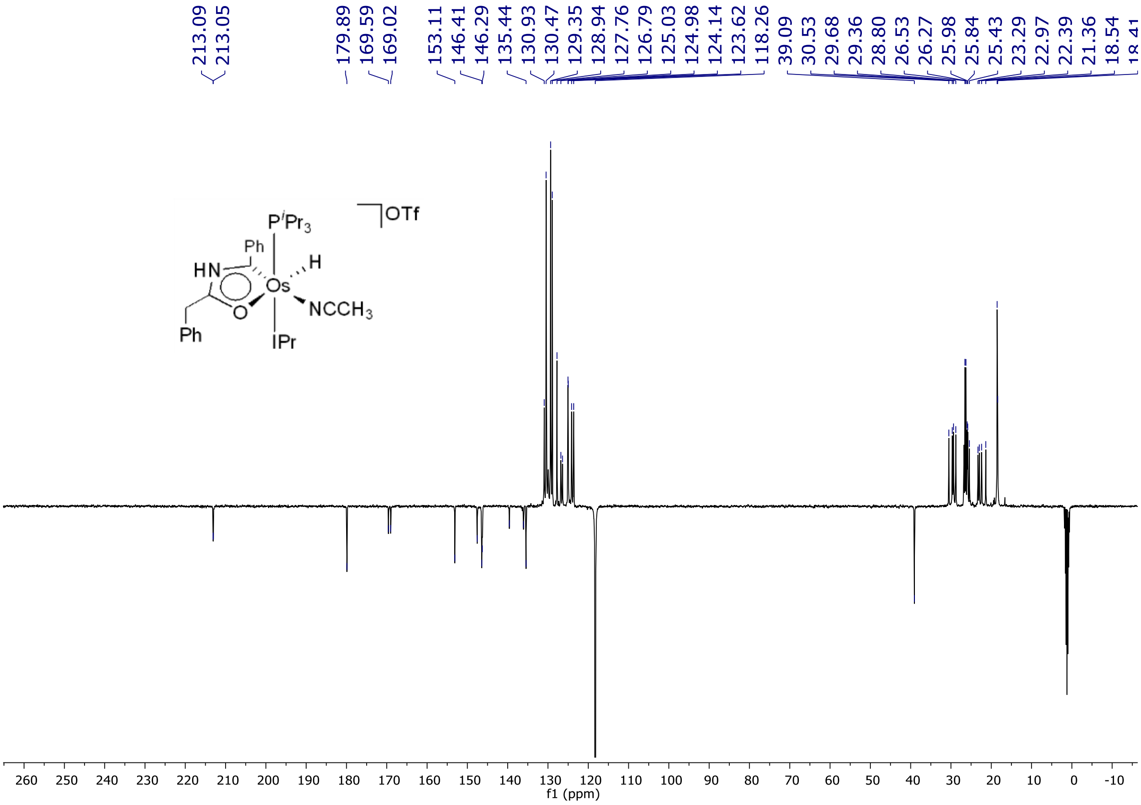

Figure S10. $^{13}C\{^{1}H\}$ spectrum (101 MHz, CD_3CN, 273 K) of compound **4**.

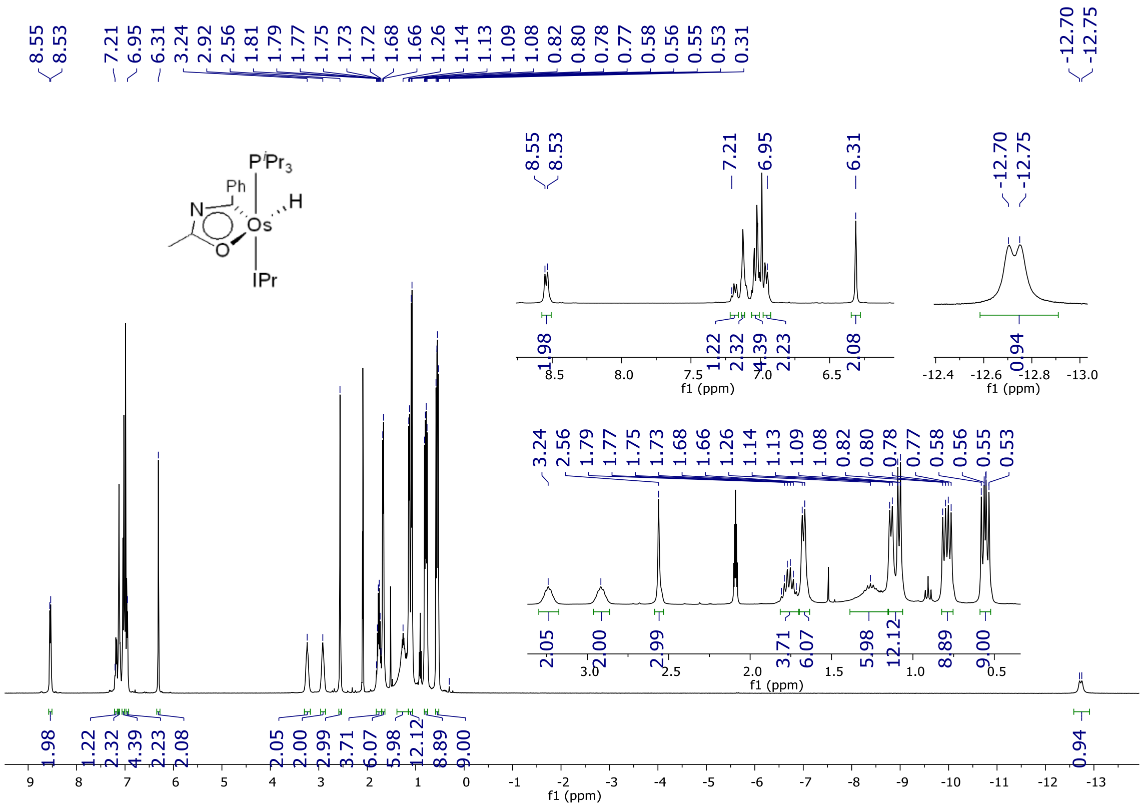

Figure S11. ^{1}H NMR spectrum (400 MHz, toluene-d_8, 253 K) of compound **5**.

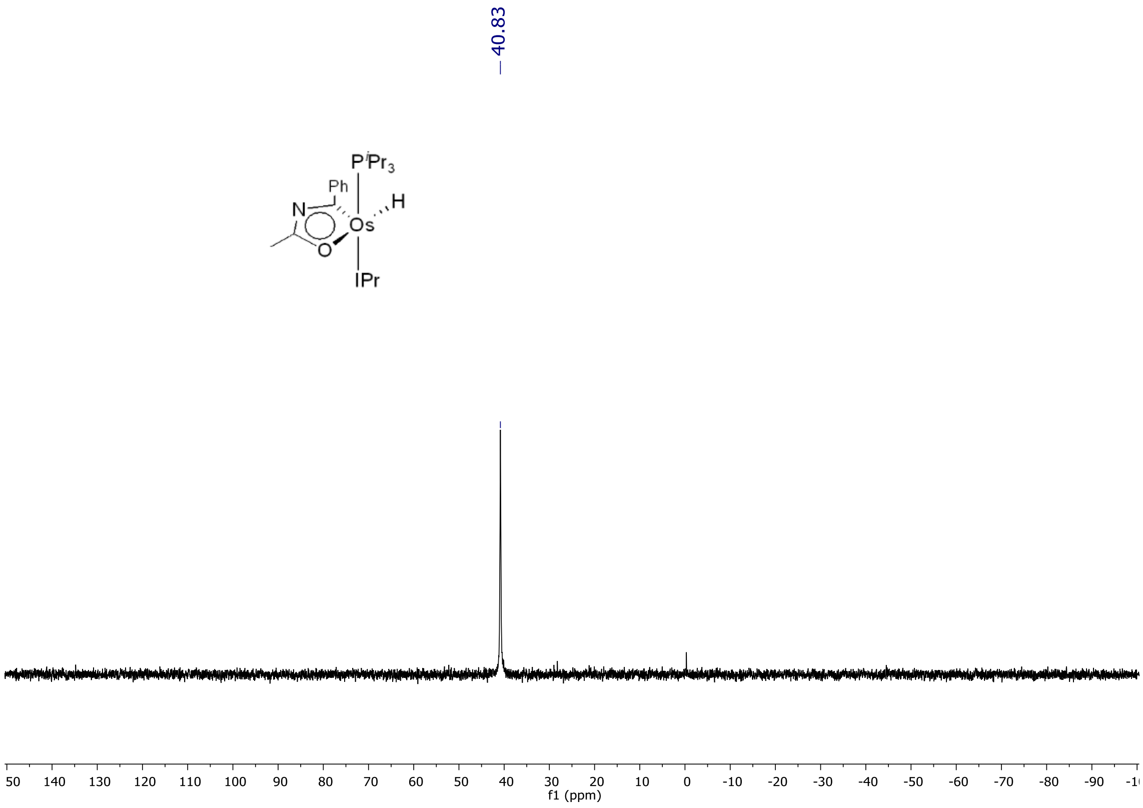

Figure S12. ^{31}P{^{1}H} spectrum (121 MHz, toluene-d_8, 298 K) of compound **5**.

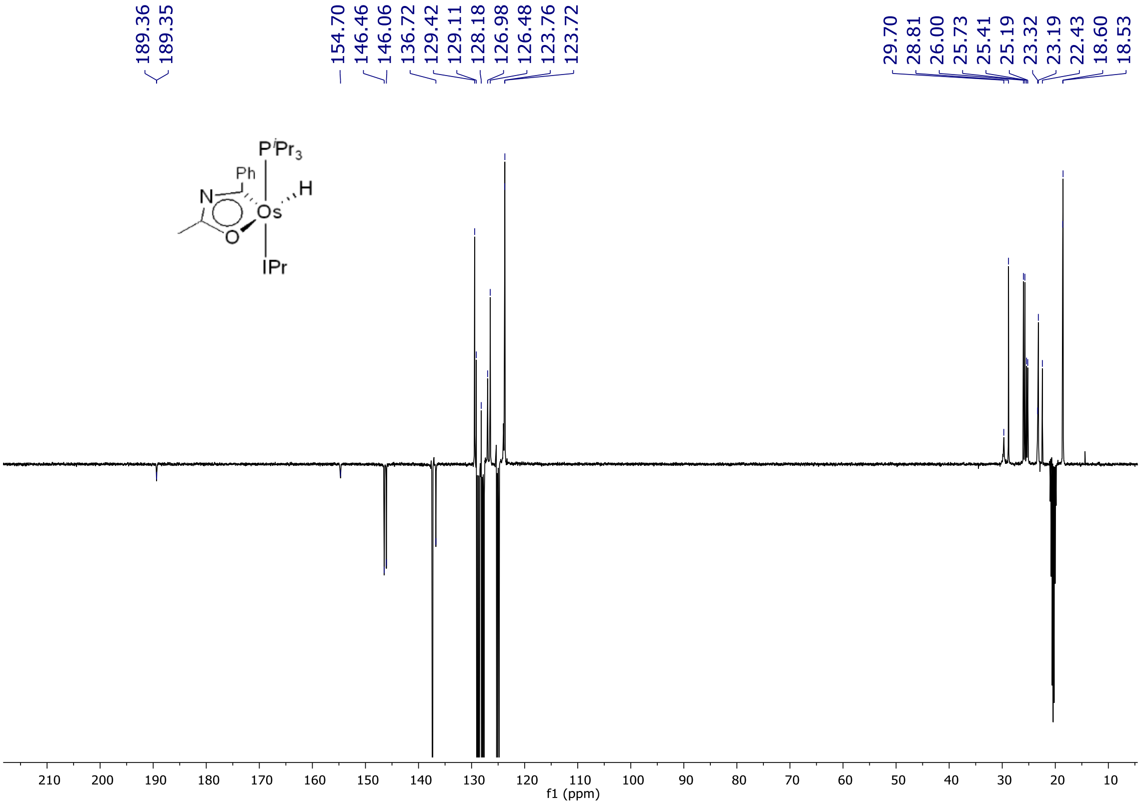

Figure S13. $^{13}C\{^{1}H\}$ spectrum (101 MHz, toluene-d_8, 253 K) of compound **5**.

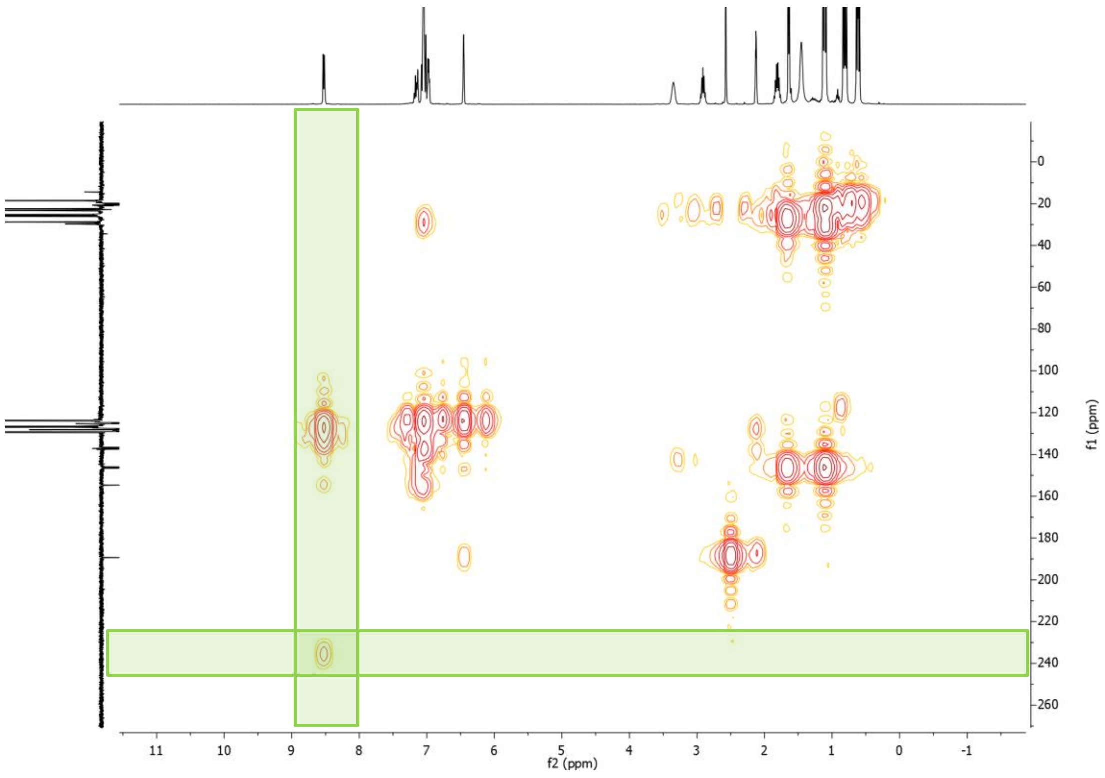

Figure S14. HMBC ^{1}H-$^{13}C\{^{1}H\}$ NRM spectrum (101 MHz, toluene-d_8, 253 K) of compound **5**.

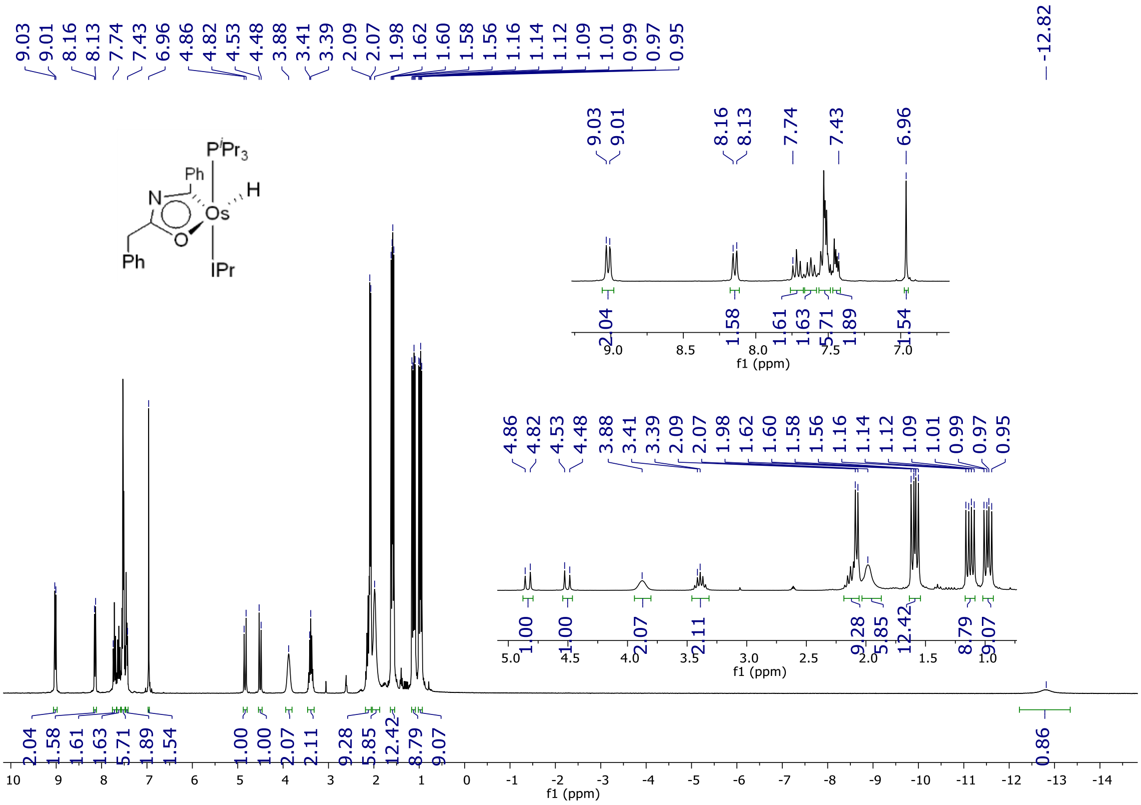

Figure S15. ^{1}H NMR spectrum (300 MHz, toluene-d_8, 298 K) of compound **6**.

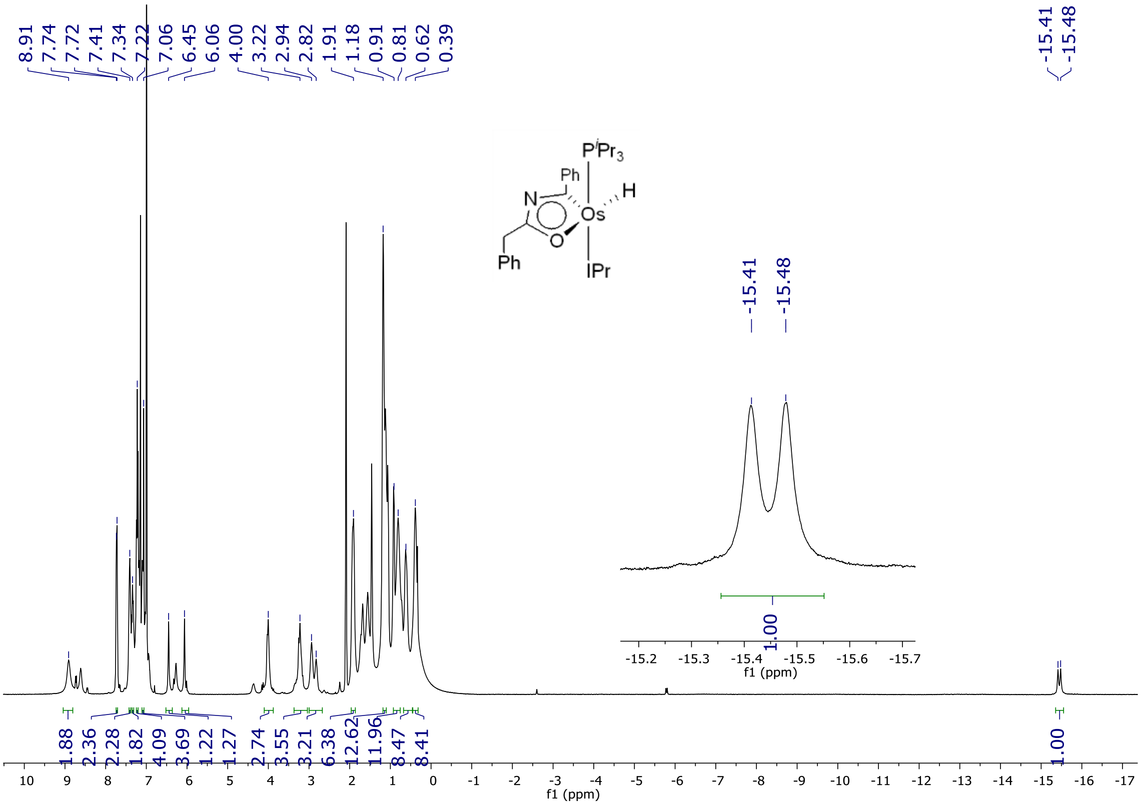

Figure S16. ^{1}H NMR spectra (400 MHz, toluene-d_8, 223K) of compound **6**.

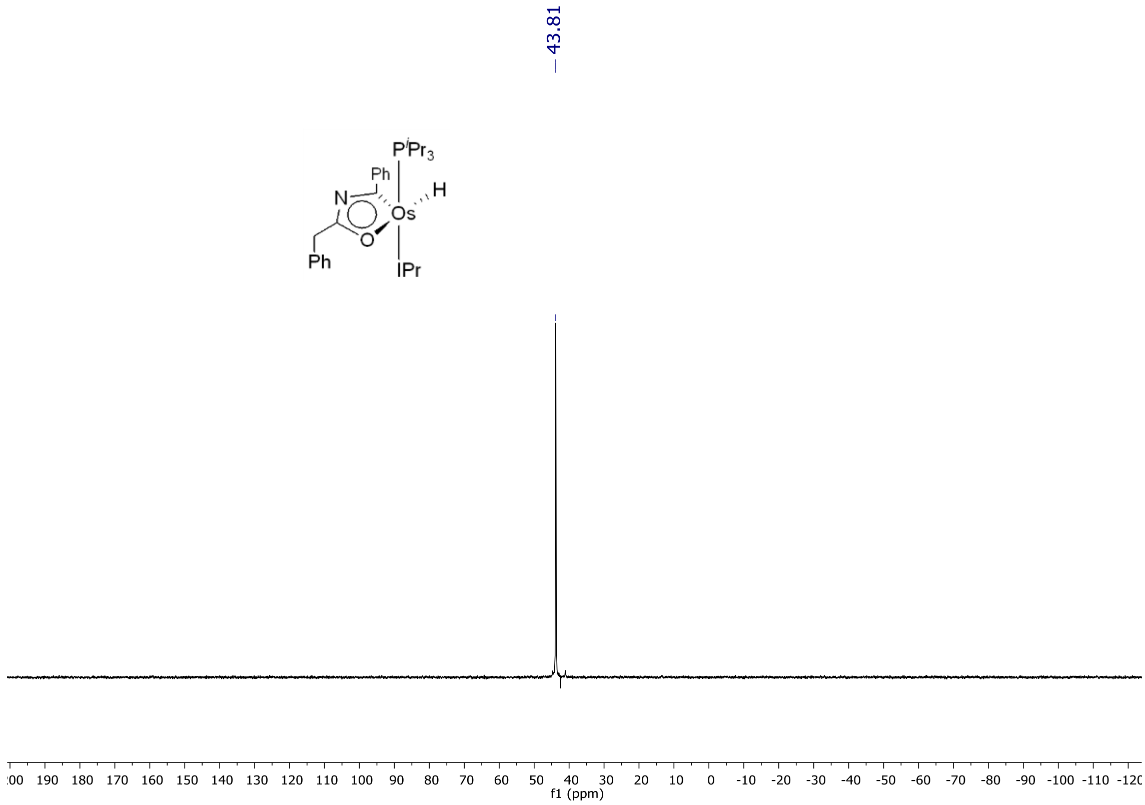

Figure S17. $^{31}P\{^1H\}$ spectrum (121 MHz, toluene-d_8, 298 K) of compound **6**.

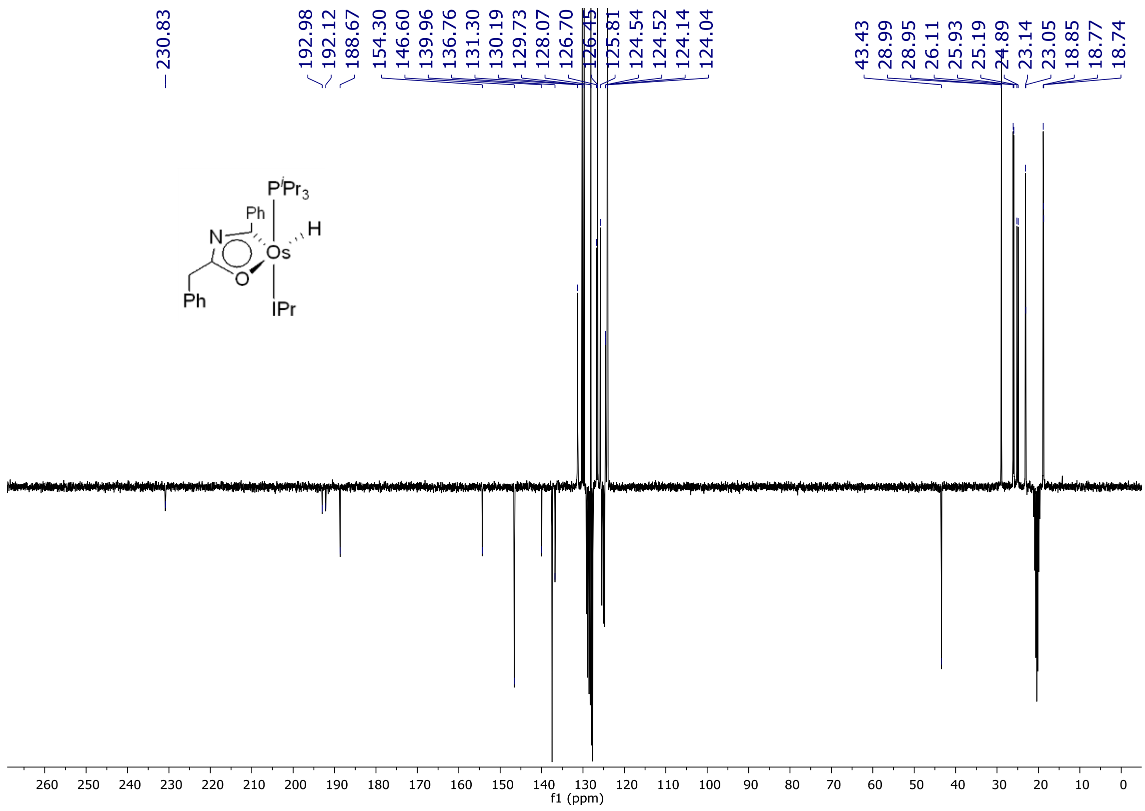

Figure S18. $^{13}C\{^1H\}$ spectrum (75 MHz, toluene-d_8, 298 K) of compound **6**.

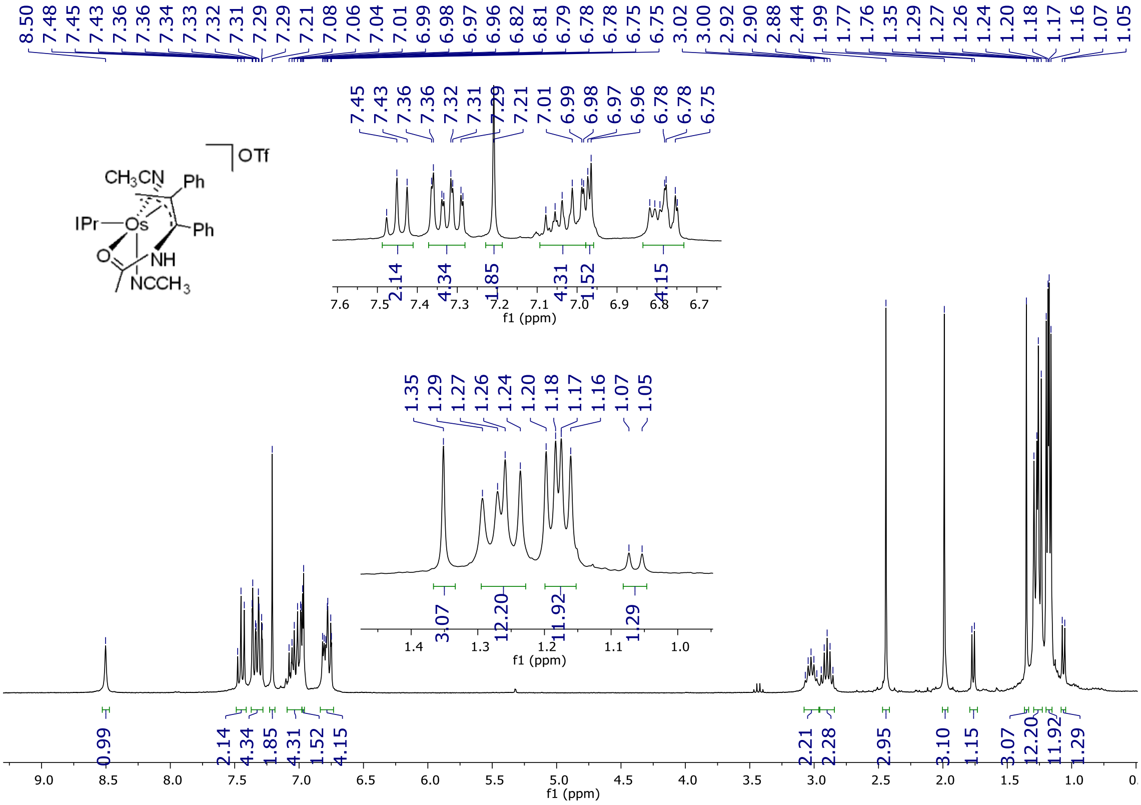

Figure S19. ^{1}H NMR spectrum (300 MHz, CD_2Cl_2, 298 K) of compound **7**.

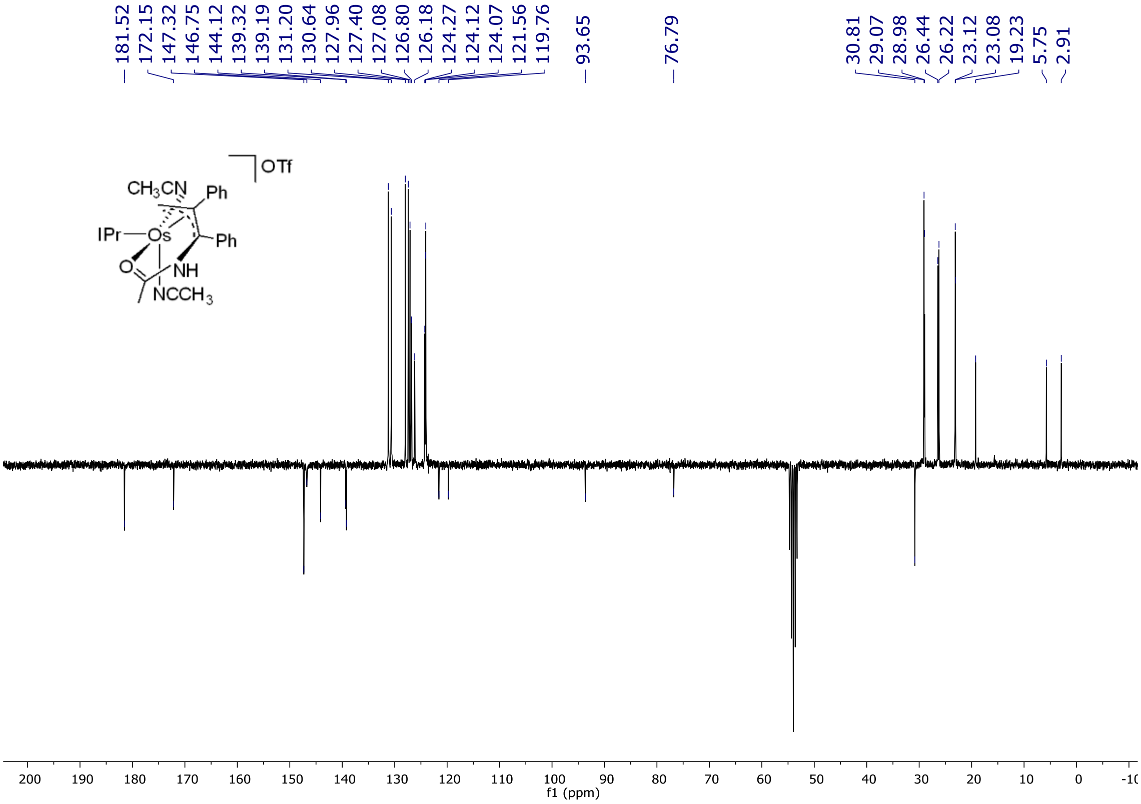

Figure S20. $^{13}C\{^{1}H\}$ spectrum (75 MHz, CD_2Cl_2, 298 K) of compound **7**.

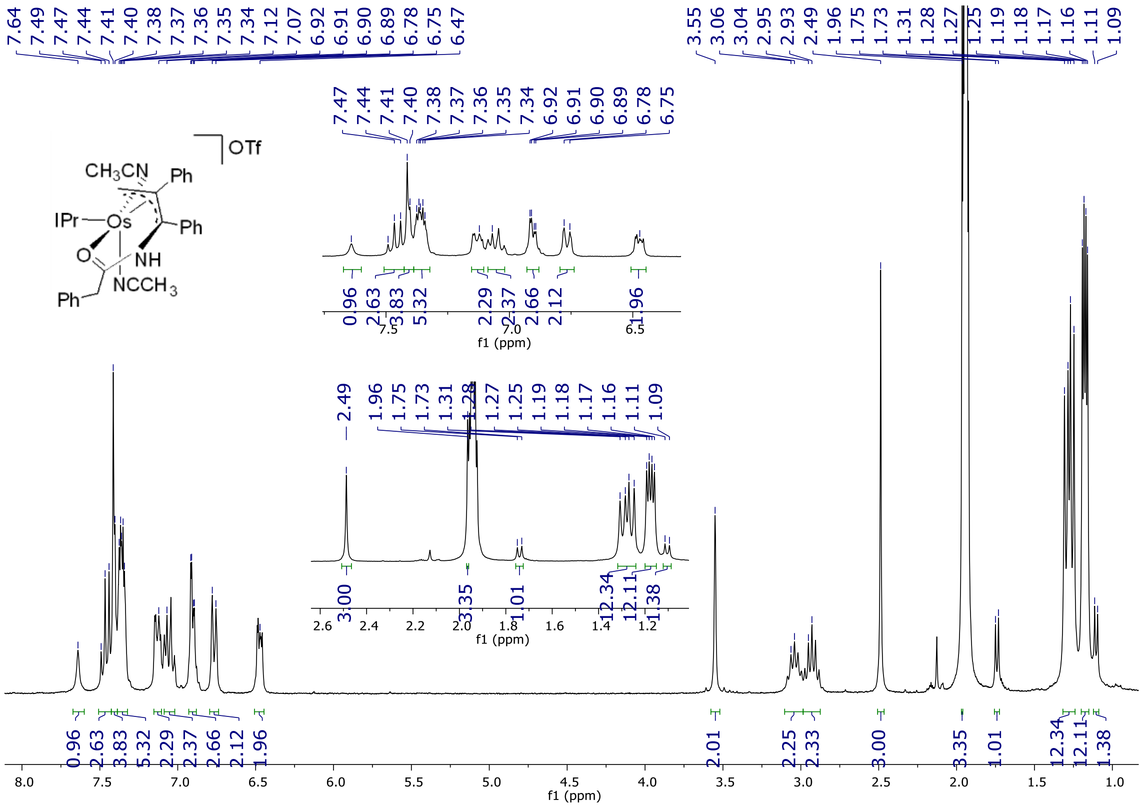

Figure S21. ^{1}H NMR spectrum (300 MHz, CD_3CN, 298 K) of compound **8**.

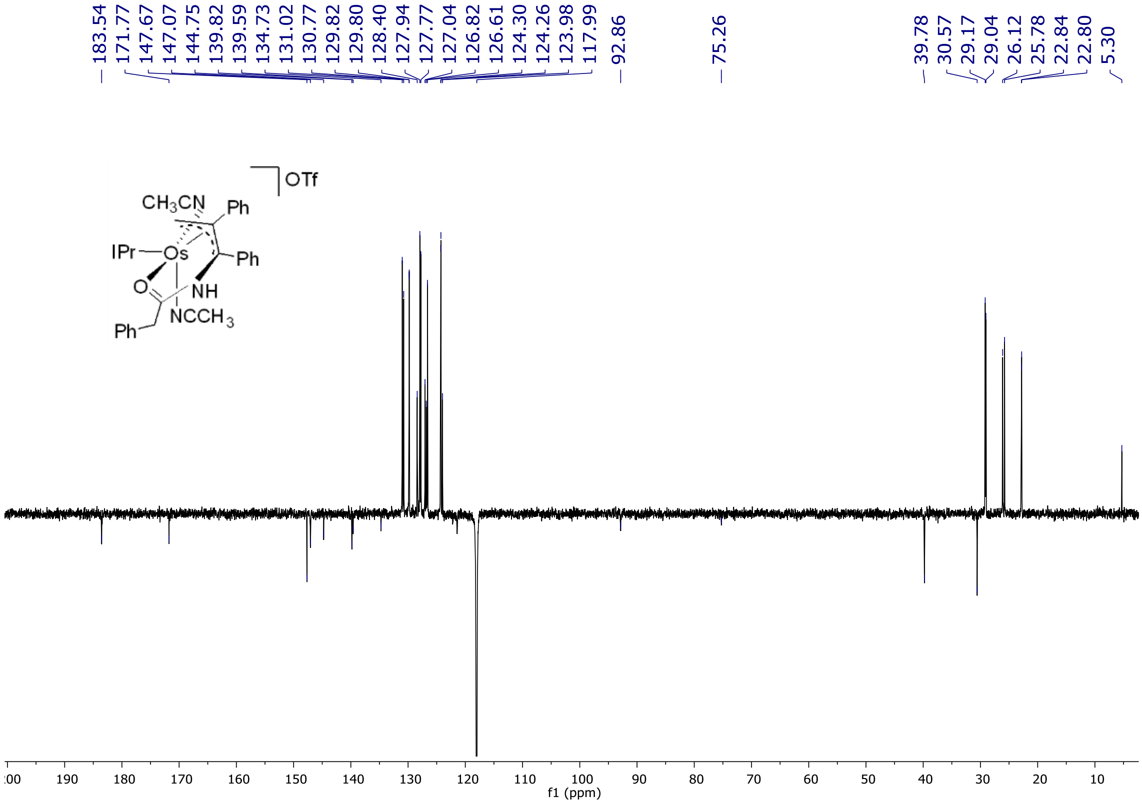

Figure S22. $^{13}C\{^1H\}$ spectrum (75 MHz, CD_3CN, 298 K) of compound **8**.

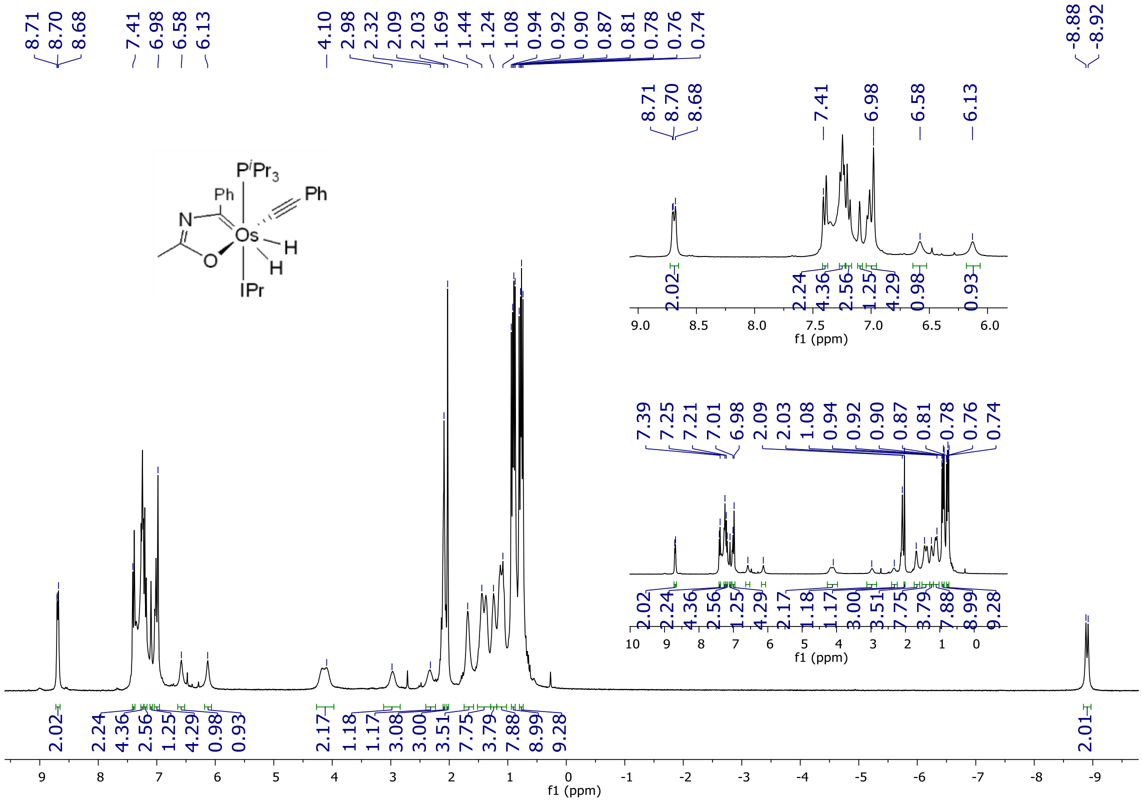

Figure S23. ^{1}H NMR spectrum (300 MHz, toluene-d_8, 298 K) of compound **9**.

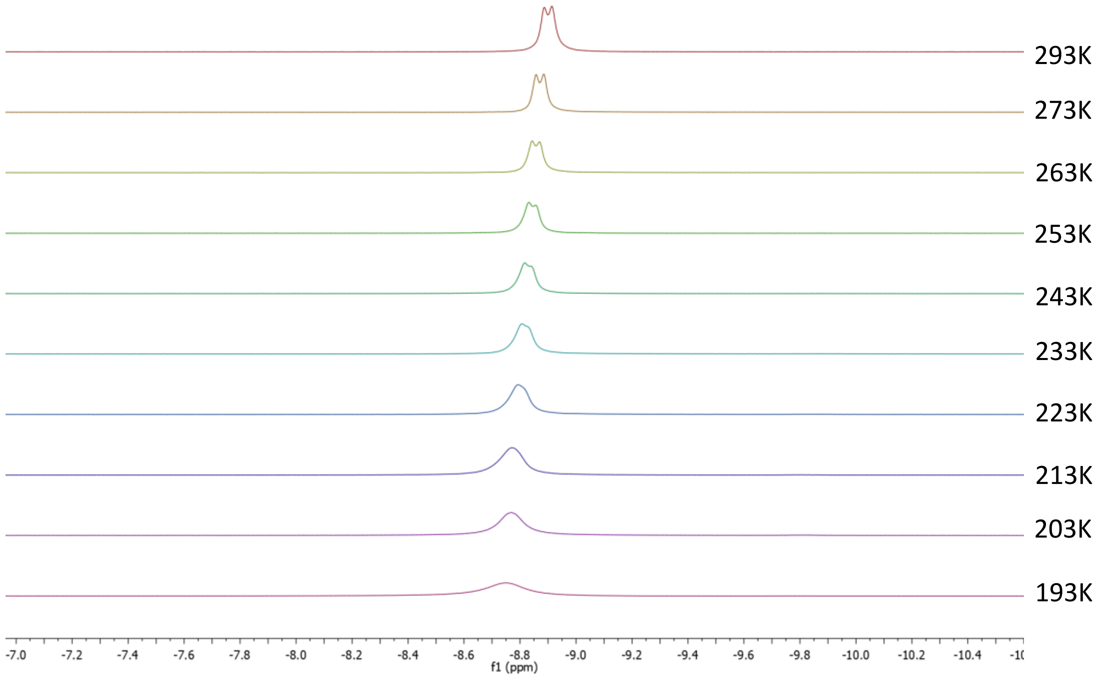

Figure S24. High field region of the ^{1}H NMR spectra (300 MHz, toluene-d_8) of compound **9** as a function of temperature.

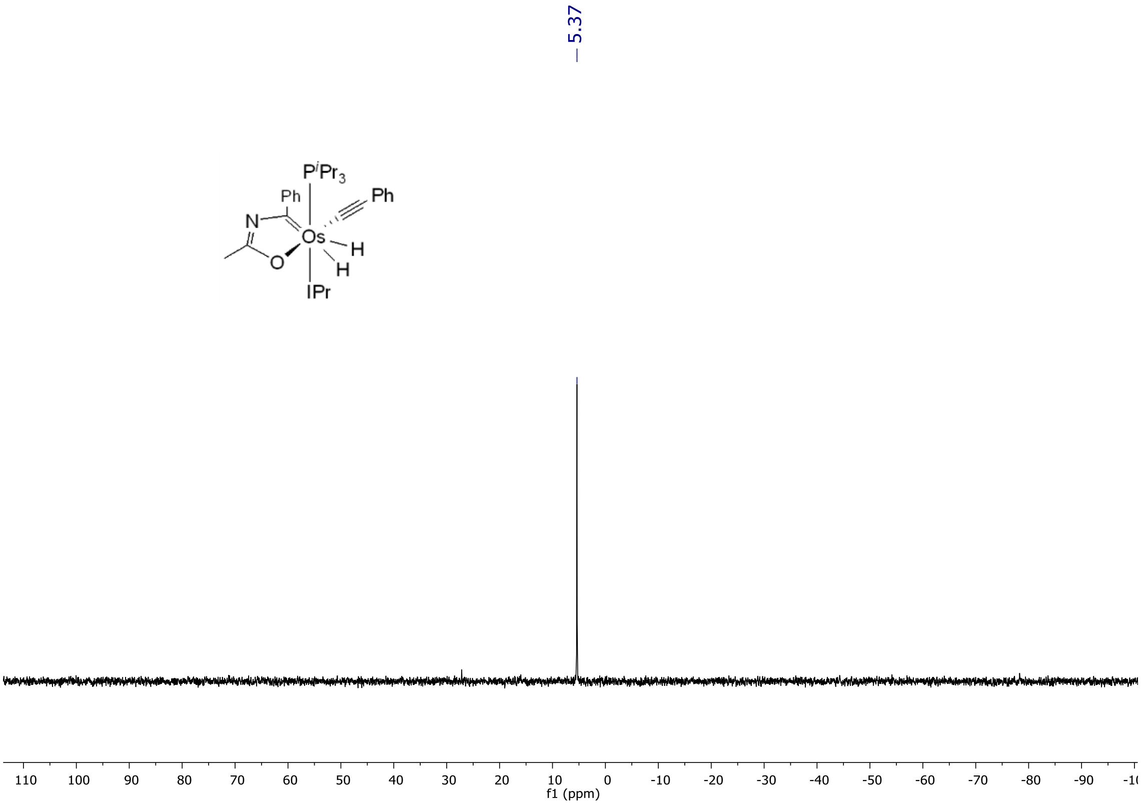

Figure S25. ^{31}P{^{1}H} spectrum (121 MHz, toluene-d_8, 298 K) of compound **9**.

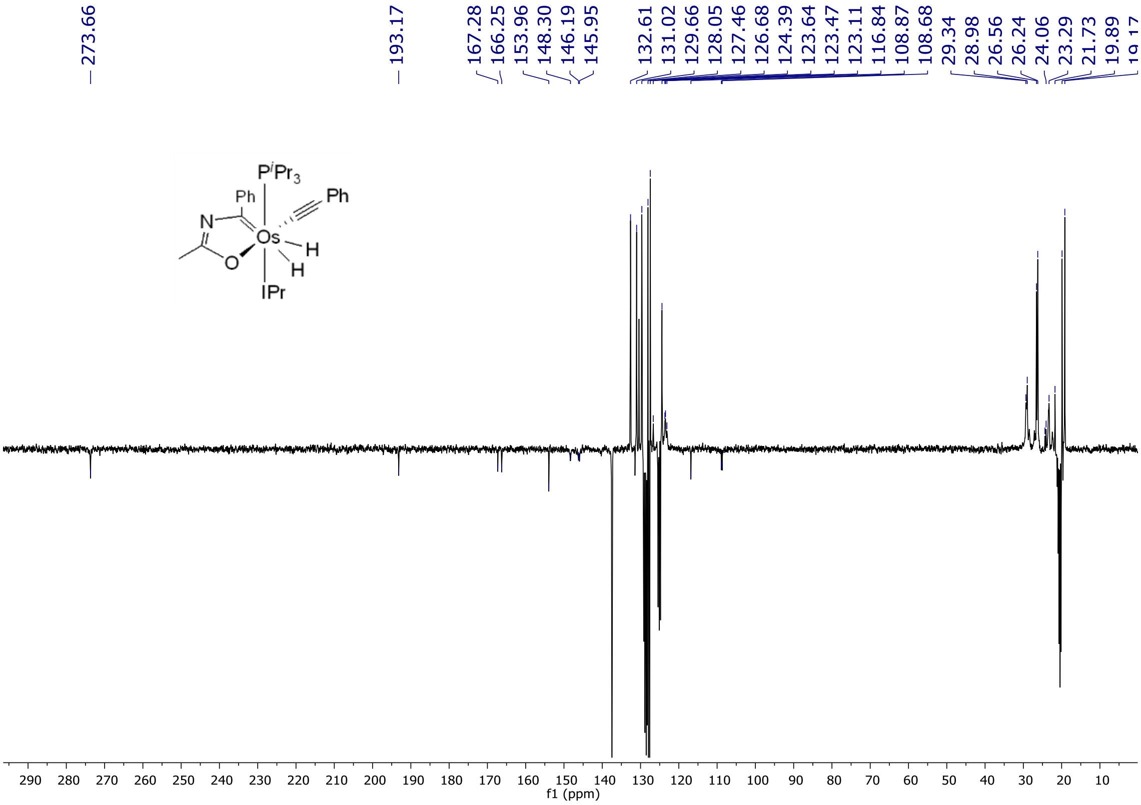

Figure S26. ^{13}C{^{1}H} spectrum (75 MHz, toluene-d_8, 298 K) of compound **9**.

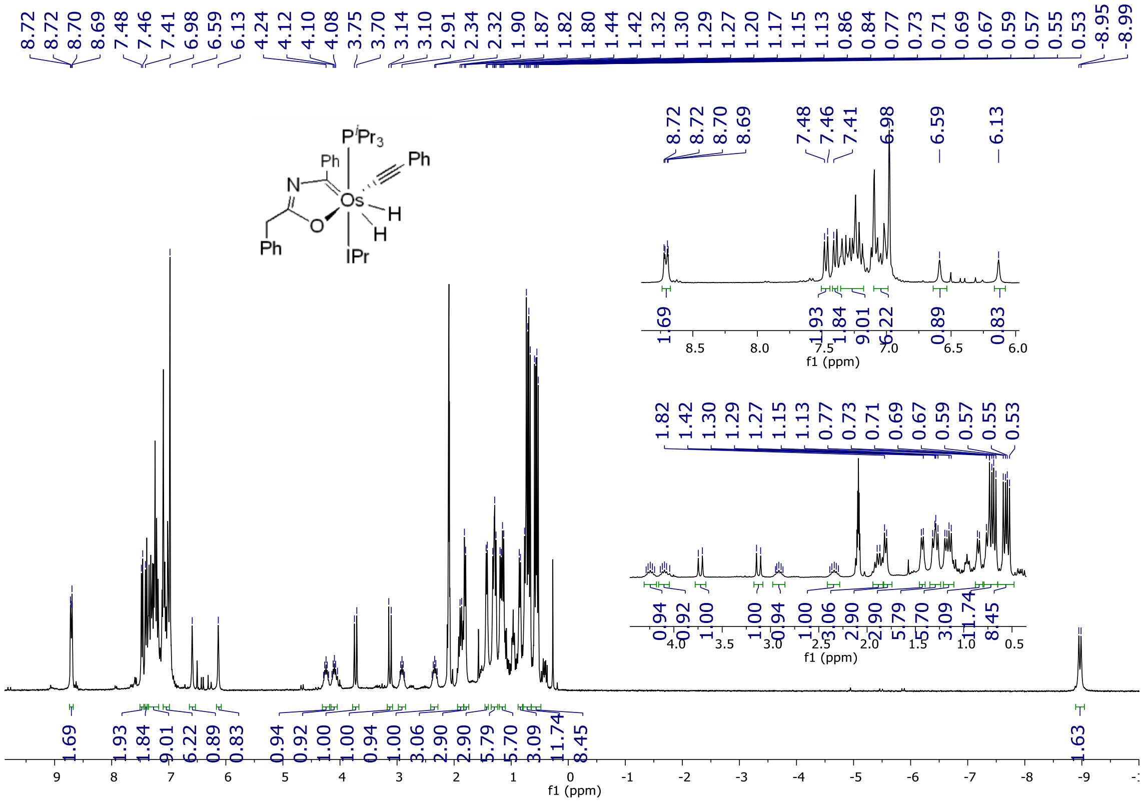

Figure S27. ^{1}H NMR spectrum (300 MHz, toluene-d_8, 298 K) of compound **10**.

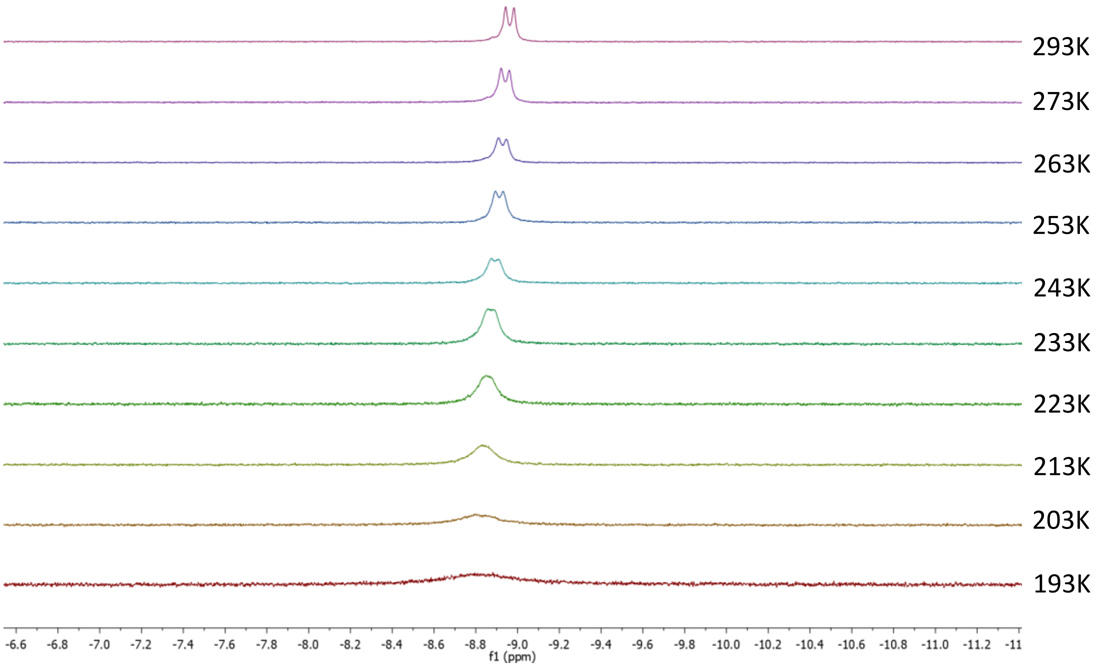

Figure S28. High field region of the ^{1}H NMR spectra (300 MHz, toluene-d_8) of compound **10** as a function of temperature.

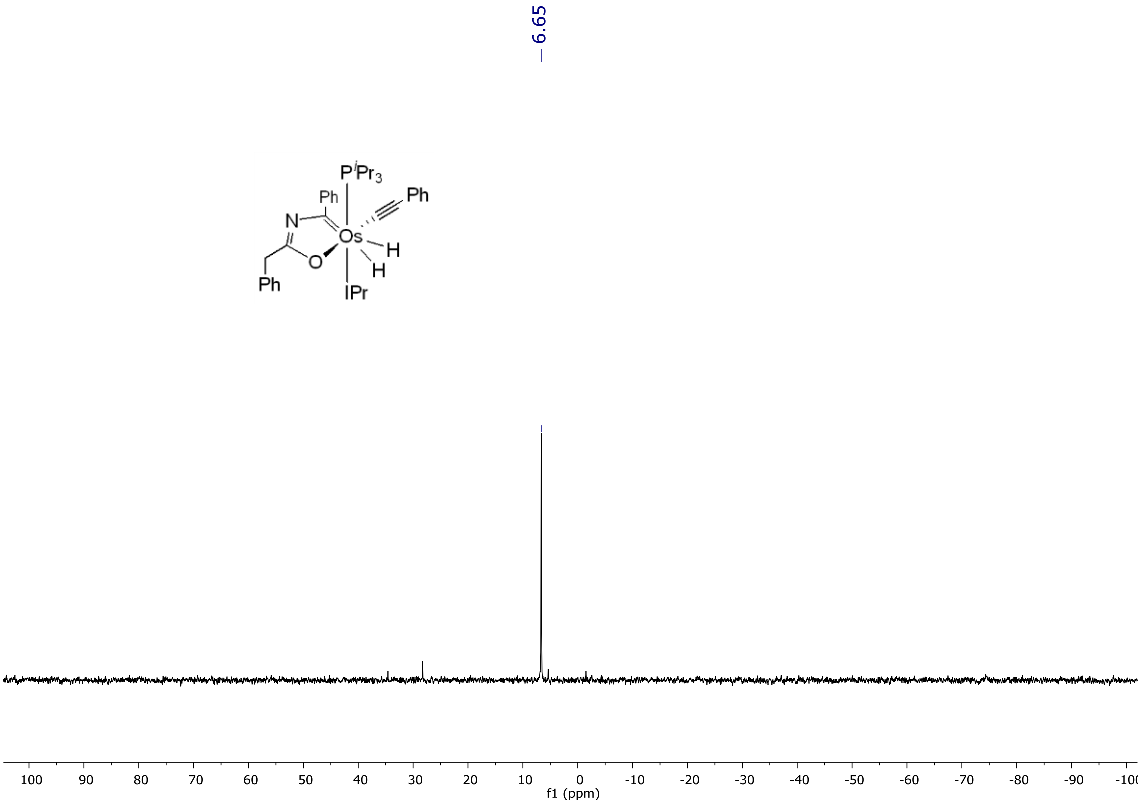

Figure S29. $^{31}P\{^1H\}$ spectrum (121 MHz, toluene-d_8, 298 K) of compound **10**.

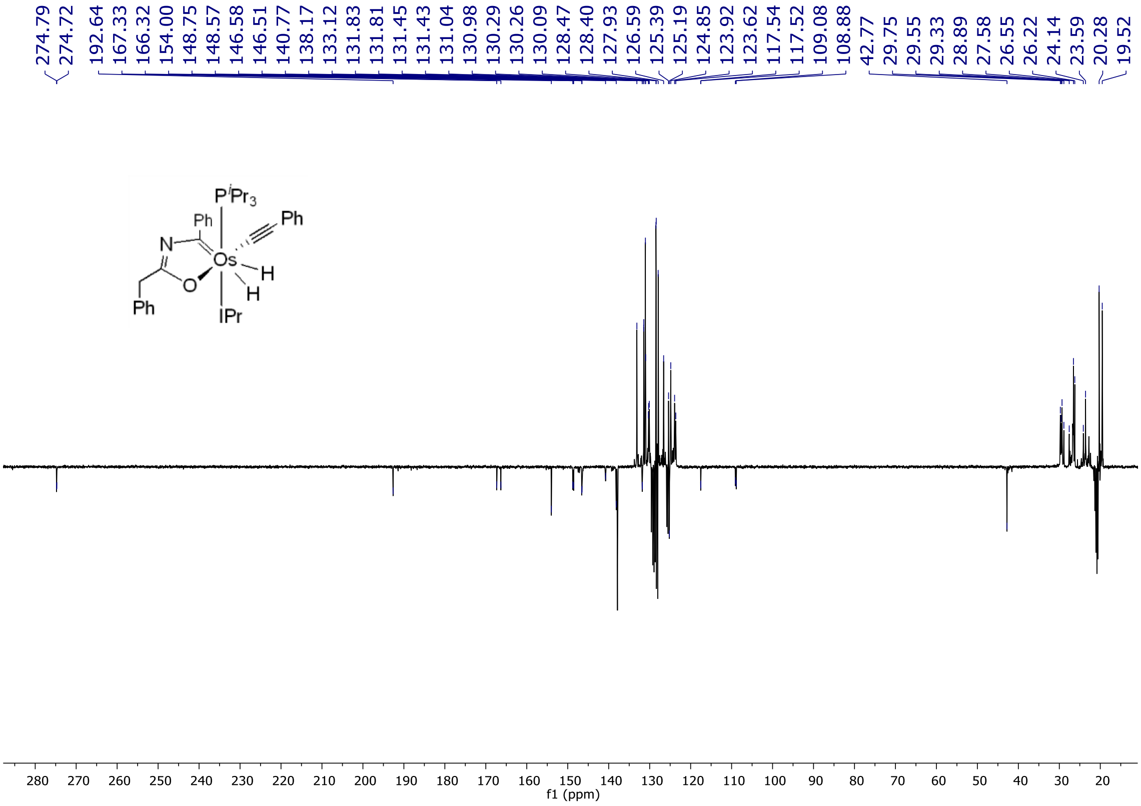

Figure S30. $^{13}C\{^1H\}$ spectrum (75 MHz, toluene-d_8, 298 K) of compound **10**.

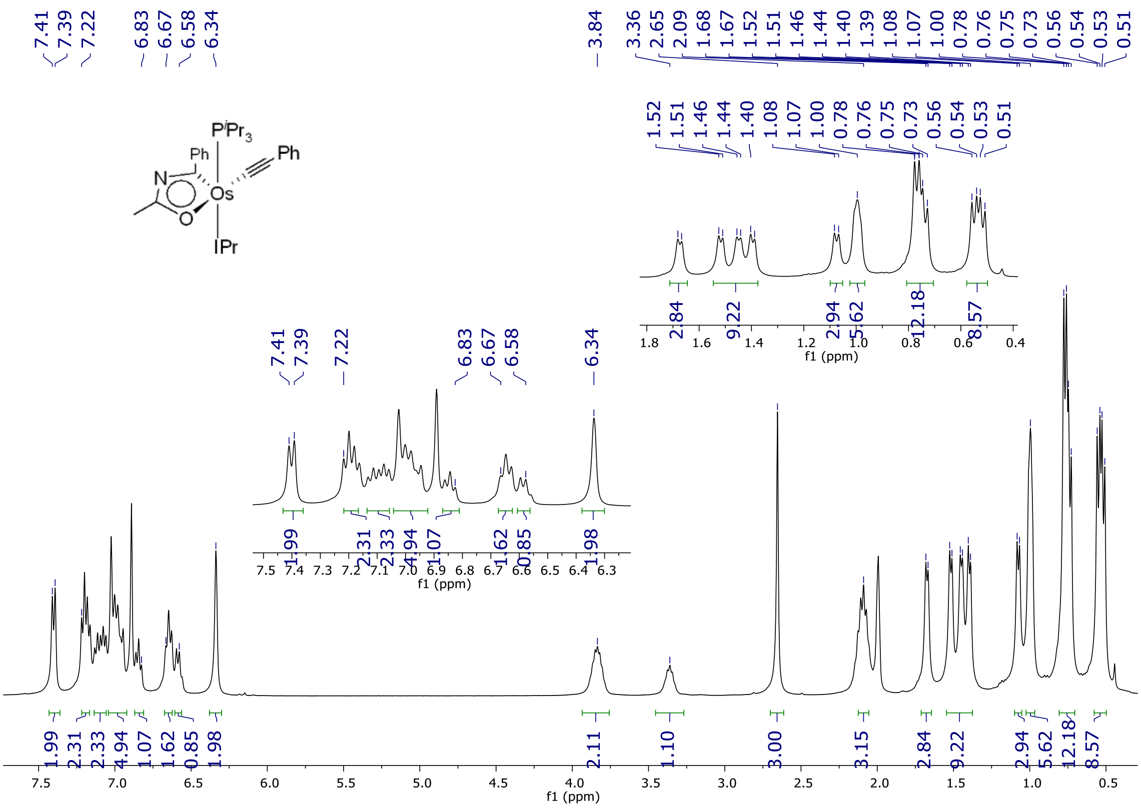

Figure S31. ^{1}H NMR spectrum (400 MHz, toluene-d_8, 263 K) of compound **11**.

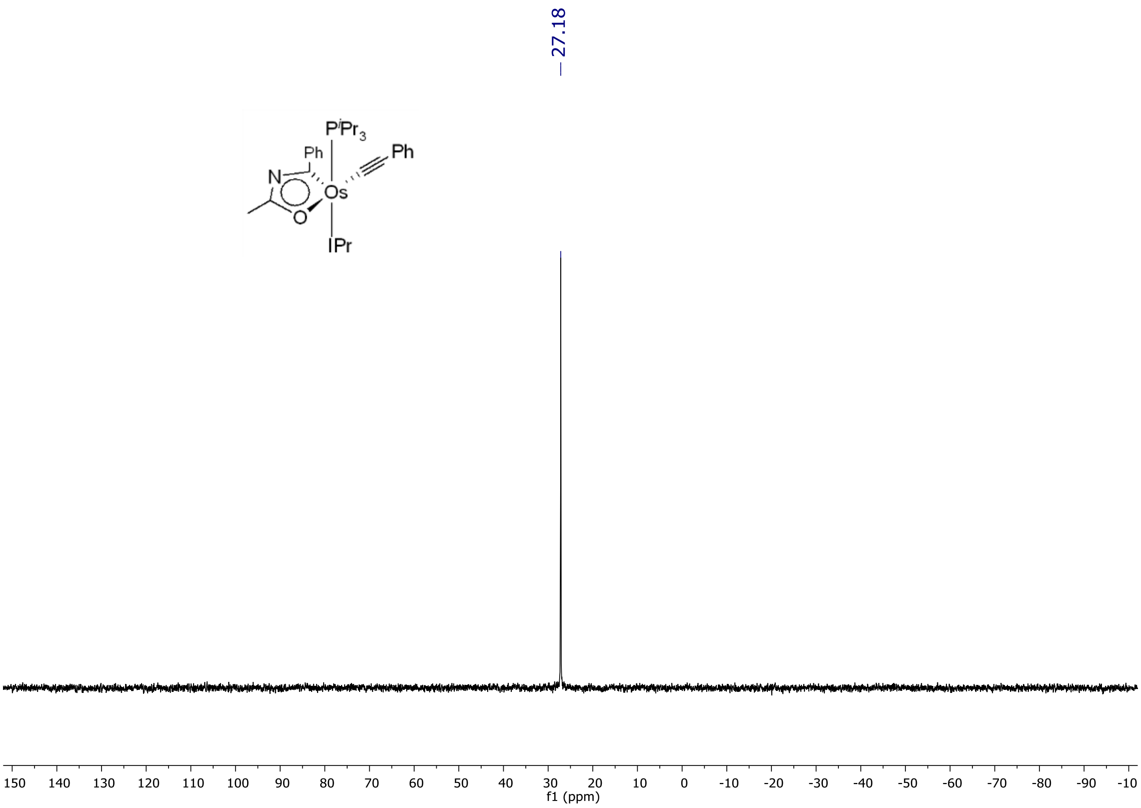

Figure S32. $^{31}P\{^{1}H\}$ spectrum (121 MHz, toluene-d_8, 298 K) of compound **11**.

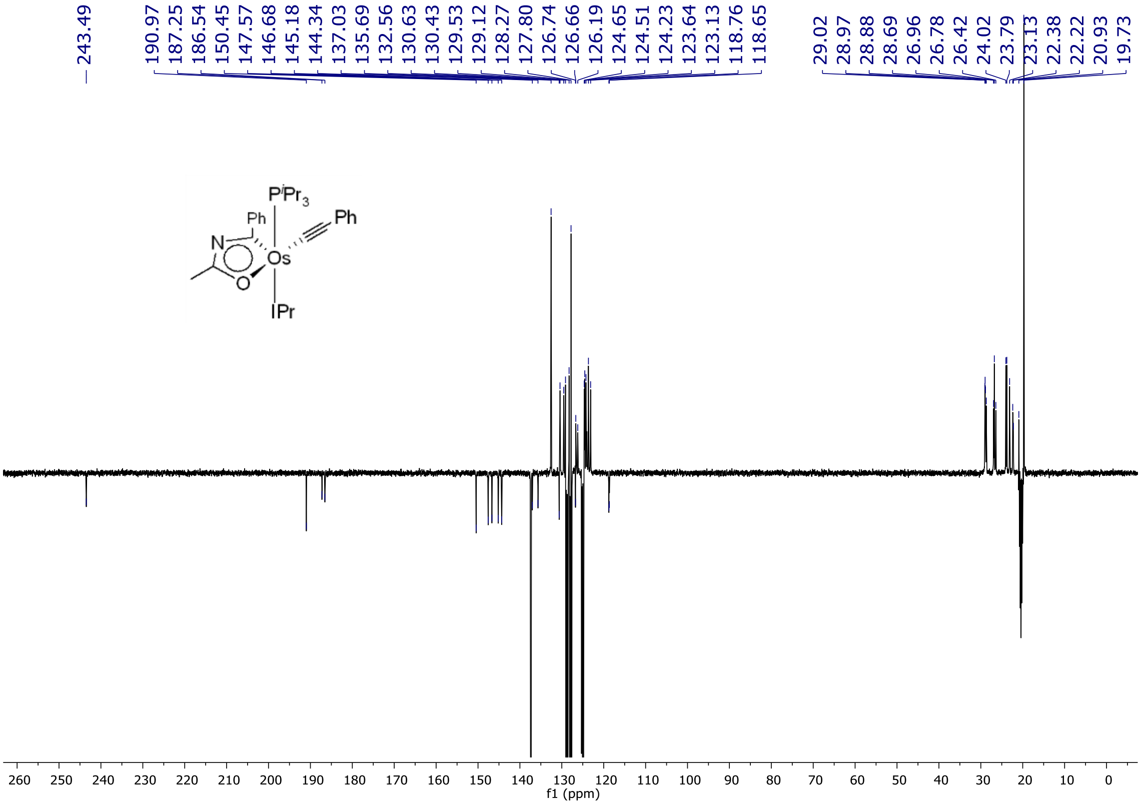

Figure S33. ^{13}C{^{1}H} spectrum (101 MHz, toluene-d_8, 263 K) of compound **11**.

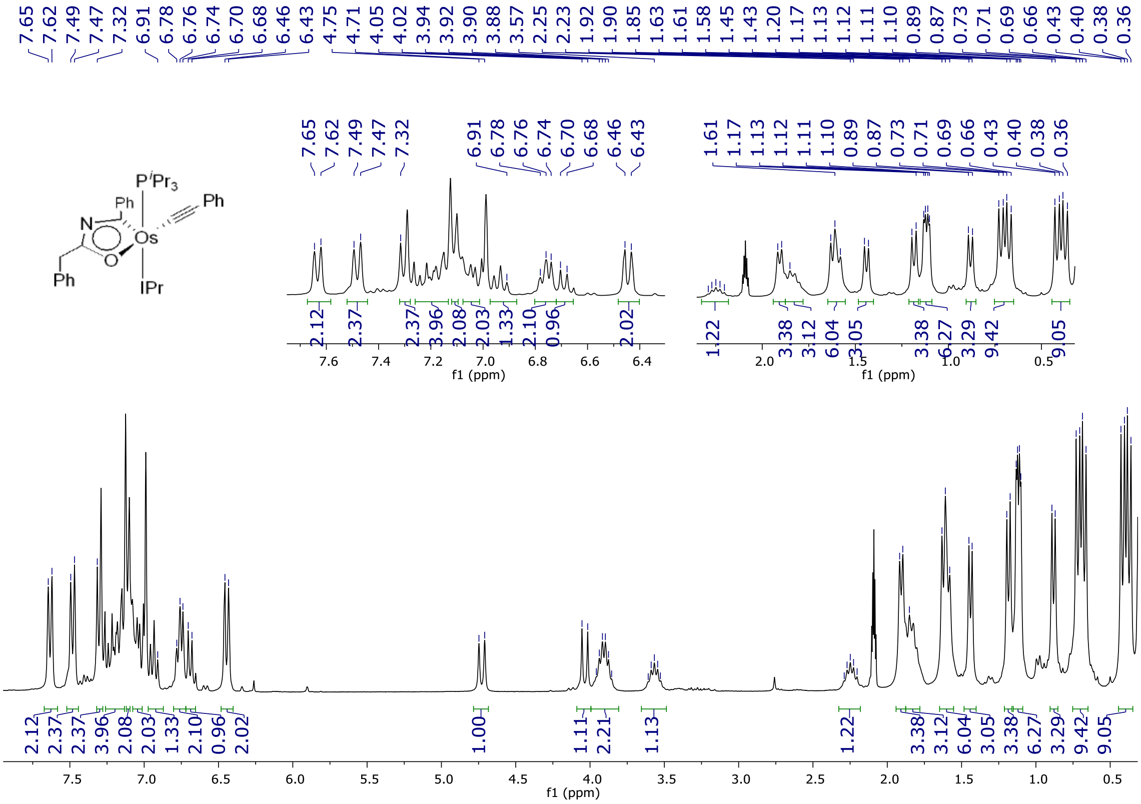

Figure S34. ^{1}H NMR spectrum (300 MHz, toluene-d_8, 263 K) of compound **12**.

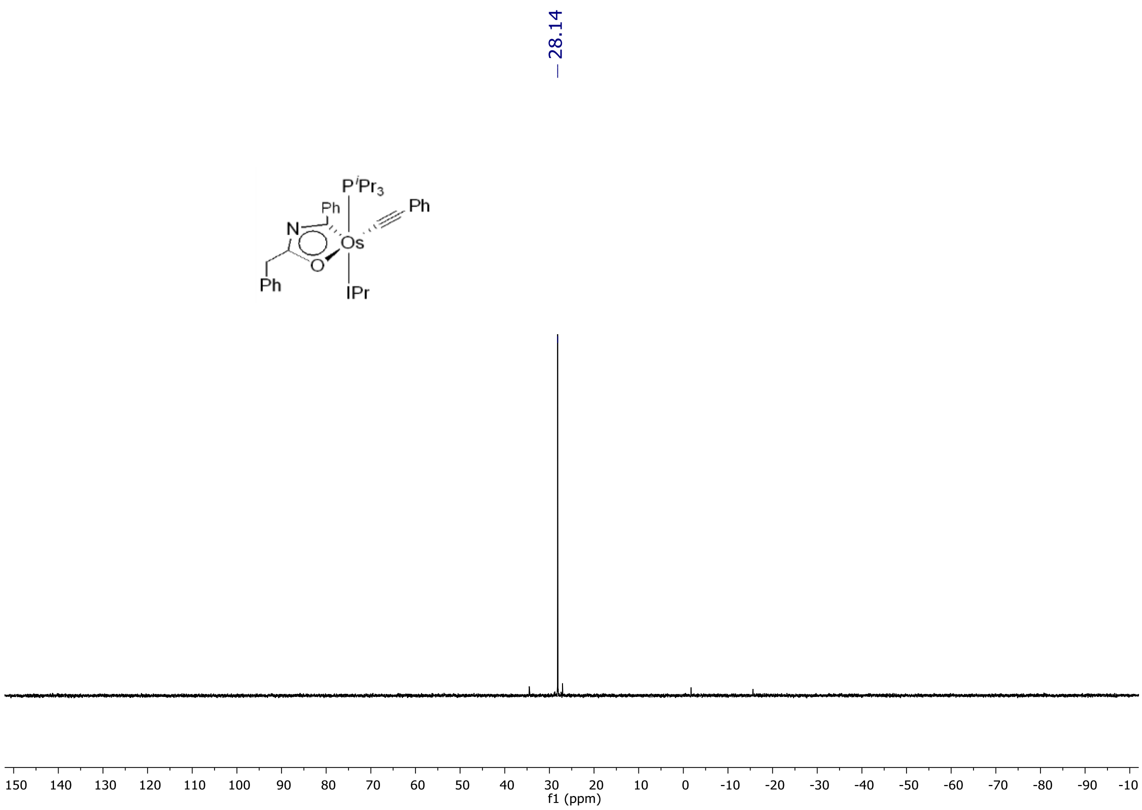

Figure S35. $^{31}P\{^{1}H\}$ spectrum (121 MHz, toluene-d_8, 298 K) of compound **12**.

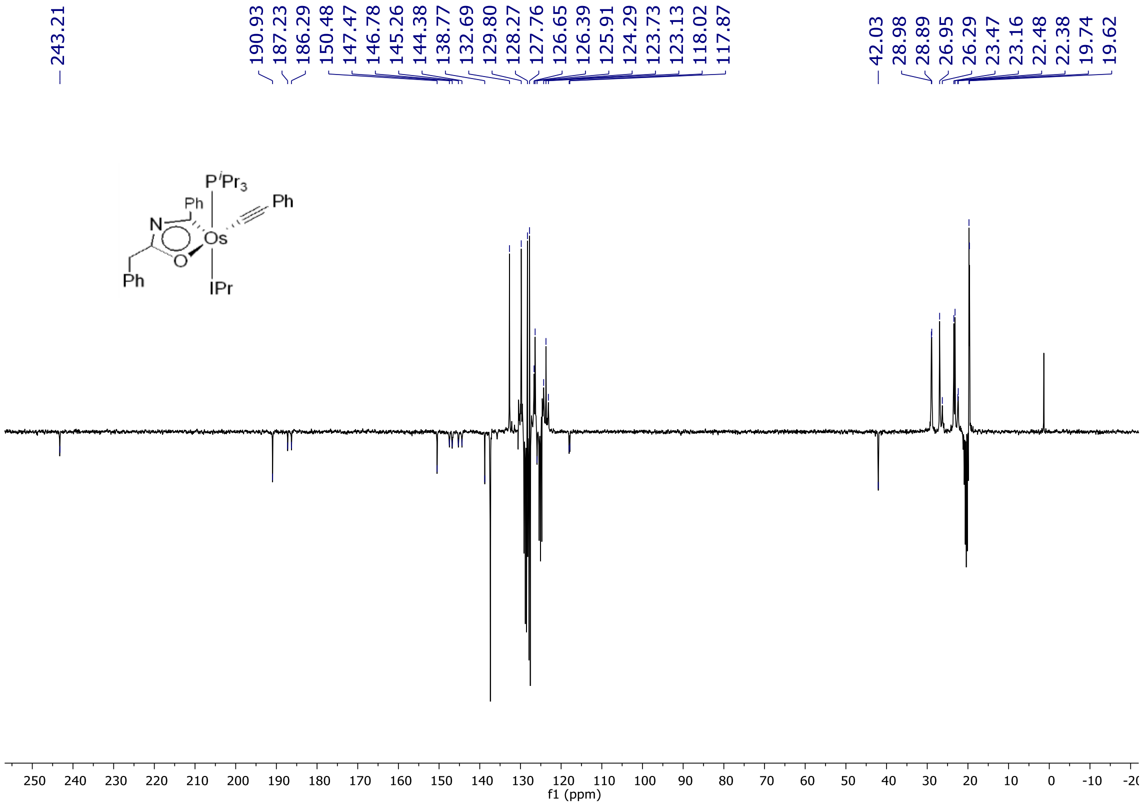

Figure S36. $^{13}C\{^{1}H\}$ spectrum (75 MHz, toluene-d_8, 263 K) of compound **12**.

- **Computational Details.**

All calculations were performed at the DFT level using the B3LYP functional[3] supplemented with the Grimme's dispersion correction D3[4] as implemented in Gaussian09.[5] Os atoms were described by means of an effective core potential SDD for the inner electron[6] and its associated double-ζ basis set for the outer ones, complemented with a set of f-polarization functions for osmium.[7] The 6-31G** basis set was used for the H, C, N, O and P.[8] All minima were verified to have no negative frequencies. The aromatic nature was further analyzed by anisotropy of the induced current density (AICD) study.[9] The AICD plots were performed with the magnetic field vector orthogonal with respect to the ring plane and points upward (clockwise currents are diatropic).

- **Energies of Optimized Structures.**

<u>Complex 2</u>

Zero-point correction= 1.085405 (Hartree/Particle)

Thermal correction to Energy= 1.146683

Thermal correction to Enthalpy= 1.147627

Thermal correction to Gibbs Free Energy= 0.992966

Sum of electronic and zero-point Energies= -2558.639060

Sum of electronic and thermal Energies= -2558.577782

Sum of electronic and thermal Enthalpies= -2558.576838

Sum of electronic and thermal Free Energies= -2558.731499

<u>Complex 6</u>

Zero-point correction= 1.104556 (Hartree/Particle)

Thermal correction to Energy= 1.165723

Thermal correction to Enthalpy= 1.166667

Thermal correction to Gibbs Free Energy= 1.009982

Sum of electronic and zero-point Energies= -2656.456791

Sum of electronic and thermal Energies= -2656.395625

Sum of electronic and thermal Enthalpies= -2656.394680

Sum of electronic and thermal Free Energies= -2656.551365

Complex 11

Zero-point correction= 1.116568 (Hartree/Particle)

Thermal correction to Energy= 1.180047

Thermal correction to Enthalpy= 1.180991

Thermal correction to Gibbs Free Energy= 1.020439

Sum of electronic and zero-point Energies= -2732.636061

Sum of electronic and thermal Energies= -2732.572583

Sum of electronic and thermal Enthalpies= -2732.571639

Sum of electronic and thermal Free Energies= -2732.732190

- **Calculated HOMO and LUMO of complexes 2 and 6.**

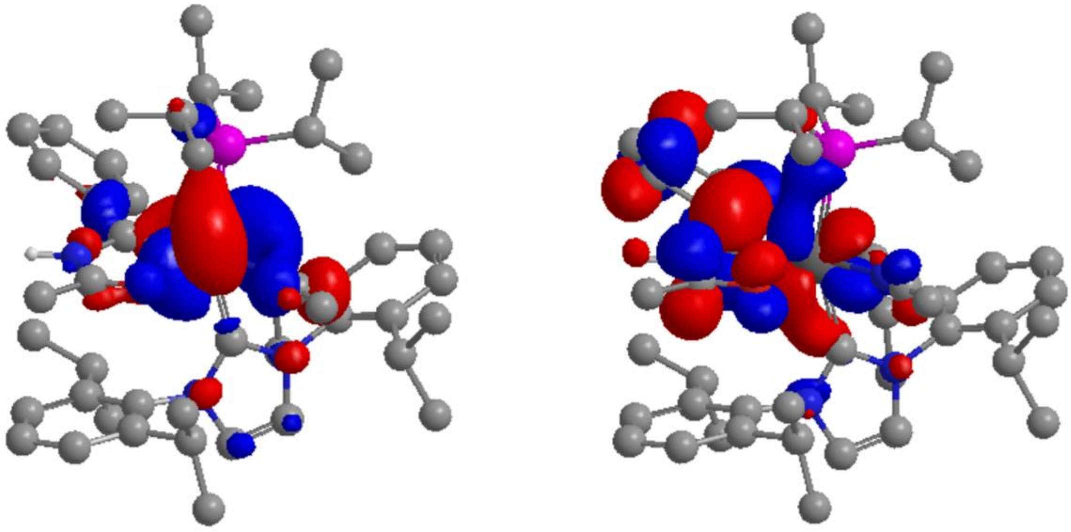

[2] HOMO **[2] LUMO**

Figure S37. HOMO and LUMO for complex **2**. Hydrogen atoms are omitted for clarity.

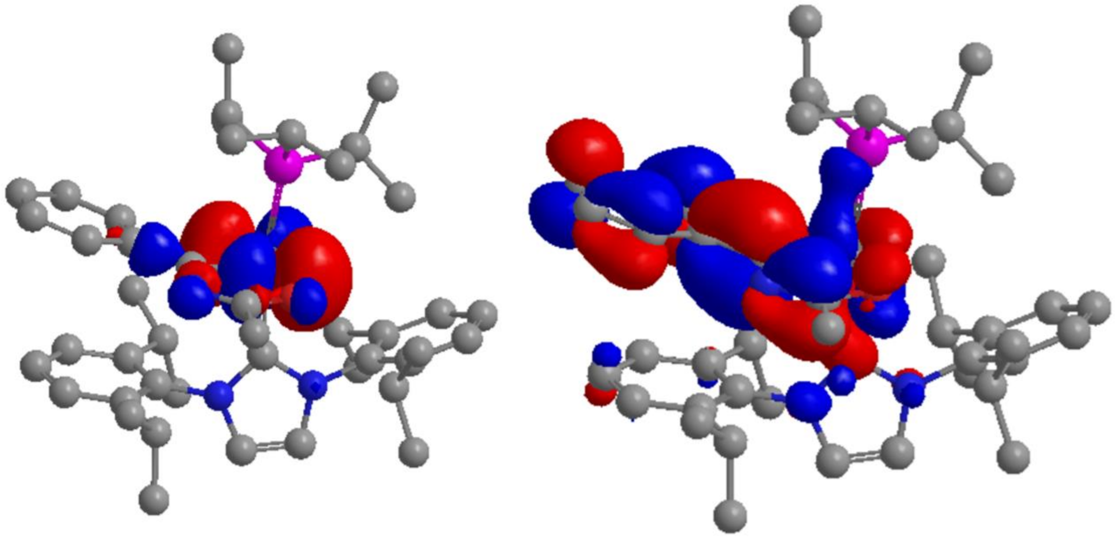

[6] HOMO **[6] LUMO**

Figure S38. HOMO and LUMO for complex **6**. Hydrogen atoms are omitted for clarity.

- **References.**

(1) Blessing, R. H. *Acta Crystallogr.* **1995**, *A51*, 33. SADABS: Area-detector absorption correction; Bruker- AXS, Madison, WI, 1996.

(2) SHELXL-2016/6. Sheldrick, G. M. *Acta Cryst.* **2008**, *A64*, 112-122.

(3) (a) Lee, C.; Yang, W.; Parr, R. G. Development of the Colle-Salvetti correlationenergy formula into a functional of the electron density. *Phys. Rev. B* **1988**, *37*, 785- 789. (b) Becke, A. D. Density-functional exchange-energy approximation with correct asymptotic behavior. *J. Chem. Phys.* **1993**, *98*, 5648-5652. (c) Stephens, P. J.; Devlin, F. J.; Chabalowski, C. F.; Frisch, M. J. Ab Initio Calculation of Vibrational Absorption and Circular Dichroism Spectra Using Density Functional Force Fields. *J. Phys. Chem.* **1994**, *98*, 11623-11627.

(4) Grimme, S.; Antony, J.; Ehrlich, S.; Krieg, H. A consistent and accurate ab initio parametrization of density functional dispersion correction (DFT-D) for the 94 elements H-Pu. *J. Chem. Phys.* **2010**, *132*, 154104.

(5) Gaussian 09, Revision D.01, M. J. Frisch, G. W. Trucks, H. B. Schlegel, G. E. Scuseria, M. A. Robb, J. R. Cheeseman, G. Scalmani, V. Barone, B. Mennucci, G. A. Petersson, H. Nakatsuji, M. Caricato, X. Li, H. P. Hratchian, A. F. Izmaylov, J. Bloino, G. Zheng, J. L. Sonnenberg, M. Hada, M. Ehara, K. Toyota, R. Fukuda, J. Hasegawa, M. Ishida, T. Nakajima, Y. Honda, O. Kitao, H. Nakai, T. Vreven, J. A. Montgomery, Jr., J. E. Peralta, F. Ogliaro, M. Bearpark, J. J. Heyd, E. Brothers, K. N. Kudin, V. N. Staroverov, T. Keith, R. Kobayashi, J. Normand, K. Raghavachari, A. Rendell, J. C. Burant, S. S. Iyengar, J. Tomasi, M. Cossi, N. Rega, J. M. Millam, M. Klene, J. E. Knox, J. B. Cross, V. Bakken, C. Adamo, J. Jaramillo, R. Gomperts, R. E. Stratmann, O. Yazyev, A. J. Austin, R. Cammi, C. Pomelli, J. W. Ochterski, R. L. Martin, K.

Morokuma, V. G. Zakrzewski, G. A. Voth, P. Salvador, J. J. Dannenberg, S. Dapprich, A. D. Daniels, O. Farkas, J. B. Foresman, J. V. Ortiz, J. Cioslowski, and D. J. Fox, Gaussian, Inc., Wallingford CT, 2013.

(6) Andrea, D.; Häußermann, U. M.; Dolg, M.; Stoll, H.; Preuss, H. Energy-adjusted ab initio pseudopotentials for the second and third row transition elements. *Theor. Chim. Acta* **1990**, *77*, 123-141.

(7) Ehlers, A. W.; Bohme, M.; Dapprich, S.; Gobbi, A.; Hollwarth, A.; Jonas, V.; Kohler, K. F.; Stegmann, R.; Veldkamp, A.; Frenking, G. A set of f-polarization functions for pseudo-potential basis sets of the transition metals SC-Cu, Y-Ag and La-Au. *Chem. Phys. Lett.* **1993**, *208*, 111-114.

(8) (a) Hehre, W. J.; Ditchfield, R.; Pople, J. A. Self-Consistent Molecular Orbital Methods. XII. Further Extensions of Gaussian-Type Basis Sets for Use in Molecular Orbital Studies of Organic Molecules. *J. Chem. Phys*. **1972**, *56*, 2257-2261. (b) Francl, M. M.; Pietro, W. J.; Hehre, W. J.; Binkley, J. S.; Gordon, M. S.; DeFrees, D. J.; Pople, J. A. Self-consistent molecular orbital methods. XXIII. A polarization-type basis set for second-row elements. *J. Chem. Phys*. **1982**, *77*, 3654-3665.

(9) (a) Geuenich, D.; Hess, K.; Köhler, F.; Herges, R. Anisotropy of the Induced Current Density (ACID), a General Method to Quantify and Visualize Electronic Delocalization. *Chem. Rev.* **2005**, *105*, 3758−3772. (b) Herges, R.; Geuenich, D. Delocalization of Electrons in Molecules. *J. Phys. Chem. A* **2001**, *105*, 3214−3220.
